# Molecular mechanisms of adaptation emerging from the physics and evolution of nucleic acids and proteins

**DOI:** 10.1093/nar/gkt1336

**Published:** 2013-12-25

**Authors:** Alexander Goncearenco, Bin-Guang Ma, Igor N. Berezovsky

**Affiliations:** ^1^CBU, University of Bergen, 5020 Bergen, Norway, ^2^Department of Informatics, University of Bergen, 5020 Bergen, Norway, ^3^Bioinformatics Institute (BII), Agency for Science, Technology and Research (A*STAR), 30 Biopolis Street, #07-01, Matrix, 138671 Singapore and ^4^Department of Biological Chemistry, Weizmann Institute of Science, Rehovot, 76100, Israel

## Abstract

DNA, RNA and proteins are major biological macromolecules that coevolve and adapt to environments as components of one highly interconnected system. We explore here sequence/structure determinants of mechanisms of adaptation of these molecules, links between them, and results of their mutual evolution. We complemented statistical analysis of genomic and proteomic sequences with folding simulations of RNA molecules, unraveling causal relations between compositional and sequence biases reflecting molecular adaptation on DNA, RNA and protein levels. We found many compositional peculiarities related to environmental adaptation and the life style. Specifically, thermal adaptation of protein-coding sequences in Archaea is characterized by a stronger codon bias than in Bacteria. Guanine and cytosine load in the third codon position is important for supporting the aerobic life style, and it is highly pronounced in Bacteria. The third codon position also provides a tradeoff between arginine and lysine, which are favorable for thermal adaptation and aerobicity, respectively. Dinucleotide composition provides stability of nucleic acids via strong base-stacking in ApG dinucleotides. In relation to coevolution of nucleic acids and proteins, thermostability-related demands on the amino acid composition affect the nucleotide content in the second codon position in Archaea.

## INTRODUCTION

More than 50 years have passed since Francis Crick in 1958 first envisioned a way of information transfer from genes to proteins, known as the central dogma of molecular biology (1). The dogma illuminates a relationship between the genotype (coding DNA sequences) and phenotype (proteins) through the mRNA that serves as an ‘interpreter’ from nucleotide to protein sequences. As a result, the phenotype secures survival, reproduction and evolution of the genotype based on the fitness and evolvability of the latter (2–4). Therefore, even though the basic information flow from genotype to phenotype has an unequivocal directionality, the ‘phenotype-to-genotype’ feedback, or in other words the epigenetic variation that facilitates genetic adaptation, is an indispensable component of molecular evolution and adaptation (5). The goal of this work is an exhaustive survey of compositional and sequence biases and their mutual influence and adjustment that underlie molecular mechanisms of adaptation of DNA, RNA and protein molecules.

Comparative analysis of genomes and proteomes is proven to be a powerful instrument in finding genes, predicting structures and functions of proteins and phylogenetic inference. Usually, orthologous sequences from the compared organisms are considered. Despite obvious importance of the comparative analysis, detection of orthologs depends strongly on the quality of sequences alignments, which is hard to control automatically for large datasets (6). Besides, overall organismal characteristics and species relatedness are not necessarily represented correctly by the resemblance of specific protein coding sequences because of ancestral gene duplication, emergence of pseudogenes and gene loss and lateral/horizontal gene transfer (7). Therefore, if the organismal level of molecular adaptation is concerned, it is important to obtain whole-genome/proteome average of every characteristic. Molecular mechanisms of adaptation in proteins are the subject of intense discussion for already several decades. The role of nucleotide content in mechanisms of adaptation of nucleic acids (8–11) as well as possible effects of nucleotide composition on the amino acid one (12–16) have been discussed in number of works (8,10,13,16–20). The (A + G) content, or so-called purine load (11,21,22) and the (G + C) content (8,10,13,16–20) were suggested as signatures of thermal adaptation in prokaryotes (21,23). It has been shown that increase of the purine load in the coding DNA is to a large extent a result of the thermal adaptation of protein sequences (22) and a signal of stabilizing stacking interactions between purine bases in rRNA (11,21,22). The origin and role of the (G + C) content is a topic of special interest. Specifically, it has been claimed that it is essentially governed by the genome replication and DNA-repair mechanisms (19), is involved into lineage- and niche-specific molecular strategies of adaptation (17), drives a codon usage (20) and even amino acid composition (12–14,16). In the case of protein structure, distinct stabilizing interactions (24–28), their structural determinants (24,29–33) and contribution from different amino acid residues (22,24,25,29,34–36) have been studied extensively (37–40). However, until recently all the studies were focused around few proteins or small set of them, individual stabilizing interactions, or considered anecdotal cases of organisms thriving under normal or extreme temperatures. First combined predictor of thermostability was proposed in Ponnuswamy *et al.* (41), and it was finally exhaustively enumerated for monomeric proteins in Zeldovich *et al.* (22) and for protein complexes in Berezovsky (42) and Ma *et al.* (43). Rapid growth of genomic data allows one to tackle topics that seemed unreachable few years ago. Here we compare the mechanisms of molecular adaptation in Archaeal and Bacterial domains of life. Profound knowledge on phylogeny, metabolism and evolutionary peculiarities of Archaea (44,45) in comparison with Bacteria was accumulated (46,47). Both Archaea and Bacteria are unicellular prokaryotic organisms, and their macromolecules are under immediate influence of the environment. It makes a comparative study of Archaeal and Bacterial compositional biases and sequence peculiarities an ideal framework for studying mechanisms of adaptation on molecular level. We analyze complete sets of their coding DNA, mRNA, tRNA, rRNA, non-coding DNA (ncDNA) and protein sequences in order to find generic trends associated with mechanisms of their adaptation as well as differences between these trends in Archaea and Bacteria. We use 244 Archaeal and Bacterial complete genomes of species with optimal growth temperatures (OGT) spanning from 8°C to 100°C and representing aerobic and anaerobic life styles.

## MATERIALS AND METHODS

### Datasets

Complete sets of coding sequences for 244 prokaryotic organisms were downloaded from NCBI Refseq and Genbank. We obtained the data on optimal OGT and aerobicity. There are many more than used organisms in the NCBI Refseq and Genbank, however the data on their OGT and aerobicity is scarce. Therefore, the size of the dataset in this study was determined by the availability of both OGT and aerobicity data for genomes, and by the demand on good coverage of the whole temperature interval. Jackknife tests performed in earlier works (22,43) showed that the number of genomes/proteomes in the dataset is sufficient for obtaining unbiased and reliable conclusions, which will persist in the future analysis with an extended set of organisms. We classified the genomes according to their domain of life: Archaea and Bacteria, temperature (psycrophiles: OGT < 24°C; mesophiles: 24°C ≤ OGT < 50°C; thermophiles: 50°C ≤ OGT < 80°C; hyperthermophiles: 80°C ≤ OGT) and oxygen tolerance (aerobic, anaeroic, facultative and microaerophilic). In calculations of correlations with OGT (and in the corresponding compositional analysis), we excluded organisms with OGT 26°C, 30°C and 37°C (116 genomes), since it has been previously shown that they are represented mainly by parasites and symbionts possessing traits unrelated to temperature adaptation (22). The compositional analysis in aerobicity was performed based on the set of 244 genomes, 146 out of them classified either as aerobic or anaerobic. Data originating from NCBI Refseq and Genbank were processed with Python programs and imported into Postgresql database with constraints for additional control of data integrity. Molecular features were calculated with Python programs and stored in the database. The *R* scripts were used for statistical analysis. The database is freely accessible for download at http://folk.uib.no/agoncear/.

#### DNA/RNA analysis

We have separated DNA and amino acid sequences of protein-coding genes, nucleic acid sequences of tRNA- and rRNA-coding genes and ncDNA sequences from the intergenic regions. We generated DNA sequences with unbiased codon usage (NCB, non-codon-bias) by uniformly choosing a codon for each amino acid from all possible codons. Dinucleotide and nucleotide compositions are not preserved in NCB sequences. We reshuffled codons in the DNA sequence (Shfld) by choosing a synonymous codon for each amino acid from the list of possible codons weighted by their genomic codon usage, hence keeping intact the amino acid sequence. This procedure preserves positional nucleotide composition and positional dinucleotide composition for positions 1-2 and 2-3, but destroys the natural frequency of 3-1 dinucleotides. Dicodon-shuffle program (48) was used to reshuffle dicodons (dShfld sequence). This procedure is applied gene-wise. It preserves amino acid sequence, positional nucleotide and dinucleotide frequencies, but destroys natural mRNA sequence. We analyzed different phases in double-stranded RNA stems. Phases I, II and III mean that respective codon positions 1, 2 and 3 in the sense and anti-sense strands are complementary ones.

We analyzed the nucleotide composition in natural, shuffled, dShfld sequences and sequences without the codon bias, calculating genomic averages for tRNA, rRNA and ncDNA regions, and for each codon position separately in protein-coding DNA. We grouped genomes based on the domain of life, oxygen tolerance and environmental temperature factors. For each group of genomes, we calculated weighted averages of the genomic compositions with the weight proportional to genome size, so that each group is represented as one meta-genome. We report the weighted averages in Supplementary File S1 and show the selected values in Table 1. The standard deviations are reported in Supplementary Files S5 and S8, along with the *P*-values for OGT correlations. *P*-values for comparison between the groups reported in the text are calculated using two-sample weighted *t*-test (Student’s *t*-test with Welch correction to the degrees of freedom, which is a standard procedure in R software for unequal variances):

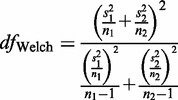

where 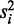 and 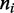 are the sample variance and the number of observations in group *i*, respectively.

**Table 1.** Compositional and sequence signals in coding and ncDNA

ARCHAEA		BACTERIA	
Characteristic	Value	Characteristic	Value
**A. Correlations between nucleotide compositions and OGT**			
*Codon position 1*			
A_Nat/NCB_	*R* = 0.69**	A_Nat/NCB_	*R* = 0.28^+^
(A+T)_Nat/NCB_	*R* = 0.68**	(A+T)_Nat/NCB_	*R* = 0.29*
(A+G)_Nat/NCB_	*R* = 0.71**	(A+G)_Nat/NCB_	*R* = 0.34*
(A+C)_Nat/NCB_	*R* = 0.71**	(A+C)_Nat/NCB_	*R* = 0.36*

*Codon position 2*			
T_Nat_	*R* = 0.55**	T_Nat_	*R* = 0.37*
T_NCB_	*R* = 0.55**	T_NCB_	*R* = 0.37*
G_Nat/NCB_	*R* = 0.60**	(T+G)_Nat_	*R* = 0.36*
(T+G)_Nat_	*R* = 0.72**	(T+G)_NCB_	*R* = 0.42**
(T+G)_NCB_	*R* = 0.68**		
(G+C)_Nat/NCB_	*R* = 0.60**		

*Codon position 3*			
A_Nat_	*R* = 0.02	A_Nat_	*R* = 0.13
A_NCB_	*R* = 0.70**	A_NCB_	*R* = 0.76**
G_Nat_	*R* = 0.22	(A+G)_Nat_	*R* = 0.60**
G_NCB_	*R* = 0.55**	(A+G)_NCB_	*R* = 0.43**
(A+G)_Nat_	*R* = 0.56**	(A+G)_Nat/NCB_	*R* = 0.11
(A+G)_NCB_	*R* = 0.67**		

**B. Position-independent dinucleotide contrasts**			

ncDNA			
ApA/TpT	1.15**	ApA/TpT	1.26**
CpC/GpG	1.24**	GpC	1.25**
GpT/ApC	0.82**		

cDNA			
		ApA	1.25**
		ApA_NCB_	1.06**
		TpT	1.20**
		TpT_NCB_	1.13**
		GpC	1.24**
		GpC_NCB_	1.07**

tRNA			
ApA	1.32**	ApG	1.28**
CpC	1.26**	TpC	1.35**
TpC	1.26**		
ApC	0.52**		
TpG	0.70**		

rRNA			
ApA	1.25**	ApA	1.15**
CpC	1.27**	CpC	1.19**

**C. Correlations of position-independent dinucleotide contrasts (DCT) with OGT**			

ncDNA			
CpT	*R* = 0.63**	GpG	*R* = 0.49
ApG	*R* = 0.64**		

cDNA			
CpT_Nat_	*R* = 0.66**	GpG_Nat_	*R* = 0.40*
CpT_NCB_	*R* = 0.57**	GpG_NCB_	*R* = 0.09
CpT_Shfld_	*R* = 0.57**	GpG_Shfld_	*R* = 0.32*
ApG_Nat_	*R* = 0.80**		
ApG_NCB_	*R* = 0.59**		
ApG_Shfld_	*R* = 0.78**		

tRNA			
ApA	*R* = 0.82*	ApA	*R* = 0.45
TpA	*R* = 0.72**		
TpT	*R* = 0.80**		
GpG	*R* = 0.44**		
CpC	*R* = 0.80**		

rRNA			
ApA	*R* = 0.92**	ApA	*R* = 0.67*
TpA	*R* = 0.88**	CpC	*R* = 0.52**
GpG	*R* = 0.72**		
CpC	*R* = 0.83**		

**D. Position-dependent dinucleotide composition ratios (Freq), dinucleotide contrasts, and their OGT correlations**			

*Codon position 1-2*			
Freq(ApG)_Nat/NCB_	1.35^n/a^	Freq(CpG)_Nat/NCB_	1.27^n/a^
Freq(CpG)_Nat/NCB_	0.62^n/a^		
ApG	1.02	TpT	1.45**
ApG_NCB_	0.79**	TpT_NCB_	1.49**
CpG	0.76**	TpA	0.69**
CpG_NCB_	1.17**	TpA_NCB_	0.65**
		GpT	0.67**
		GpT_NCB_	0.67**
ApG	*R* = 0.77**		
ApG_NCB_	*R* = 0.11		
GpC	*R* = 0.55**		
GpC_NCB_	*R* = 0.44*		
CpC	*R* = 0.37^+^		
CpC_NCB_	*R* = 0.37^+^		
ApG_Nat/NCB_	*R* = 0.63**		
GpC_Nat/NCB_	*R* = 0.60**		
CpC_Nat/NCB_	*R* = 0.37^+^		
			
RpR	*R* = 0.40*	RpR	*R* = 0.33*
RpR_NCB_	*R* = 0.15	RpR_NCB_	*R* = 0.27^+^
YpY	*R* = 0.34*	YpY	*R* = 0.39*
YpY_NCB_	*R* = −0.05^+^	YpY_NCB_	*R* = 0.33*
RpR_Nat/NCB_	*R* = 0.59**	RpR_Nat/NCB_	*R* = 0.37*
YpY_Nat/NCB_	*R* = 0.64**	YpY_Nat/NCB_	*R* = 0.45**

*Codon position 2-3*			
TpA	*R* = 0.63**	ApA	1.50**
TpA_NCB_	*R* = −0.28	ApA_NCB_	1.05**
CpT	*R* = 0.61**	GpC	1.33**
CpT_NCB_	*R* = 0.68**	GpC_NCB_	0.98
ApG	*R* = 0.69**	ApC_Nat/NCB_	*R* = 0.52**
ApG_NCB_	*R* = 0.37^+^	GpA_Nat/NCB_	*R* = 0.41*
			
RpR	*R* = 0.20	RpR	*R* = 0.51**
RpR_NCB_	*R* = 0.65**	RpR_NCB_	*R* = 0.73**
YpY	*R* = 0.28	YpY	*R* = 0.58**
YpY_NCB_	*R* = 0.66**	YpY_NCB_	*R* = 0.75**
		RpR_Nat/NCB_	*R* = 0.39*
		YpY_Nat/NCB_	*R* = 0.43**

*Codon position 3-1*			
CpT	*R* = 0.60**		
CpT_Shfld_	*R* = 0.05		
ApG	*R* = 0.53*		
ApG_Shfld_	*R* = −0.02		

RpR	*R* = 0.30^+^	RpR	*R* = 0.48**
RpR_Shfld_	*R* = 0.20	RpR_Shfld_	*R* = 0.01
YpY	*R* = 0.35^+^	YpY	*R* = 0.56**
YpY_Shfld_	*R* = −0.06	YpY_Shfld_	*R* = 0.17

Pearson correlation coefficient is denoted by *R*; DCT is calculated as the ratio of dinucleotide frequency to the product of frequencies of the corresponding independent nucleotides. Nucleotides with purine (A or G) and pyrimidine (T or C) bases are denoted with R and Y, respectively. The lower index distinguishes the values observed for natural sequences (Nat), sequences with eliminated codon bias (NCB), values observed after shuffling of amino acid sequences (Shfld). If the lower index is omitted, the value is given for the natural sequences. The *P*-values for correlations and for the dinucleotide contrast *t*-tests (*H*_0_: DCT is 1.0) are shown in superscripts as significance levels: +*P*-value < 0.05, * < 0.01, ** < 0.0001. Supplementary Files S5 and S7 list all *P*-values for position-specific correlations, SD for compositions and the *P*-values for *t*-tests. Supplementary Files S8 and S9 show the *P*-values for position-independent contrast *t*-tests and correlations.

Supplementary File S6 describes the tests for (G + C)_3_ composition in connection to oxygen tolerance. In Supplementary Files S7 and S9 one-sample weighted *t*-test *P*-values are reported for the comparisons of nucleotide compositions with 0.25 and 0.5 for nucleotides and nucleotide combinations, respectively. The significance of dinucleotide contrasts (DCT) is assessed by comparing the weighted DCT averages to 1, and it is also reported in the same supplementary files.

#### RNA folding in silico

The ‘RNAfold’ program (49) in the ‘Vienna RNA Package’ was used for performing the RNA folding. Native and randomized sequences were folded using Zuker’s energy minimization algorithm (50), which determines the folding free energy for the most favorable conformation from a vast number of possible simulated structures. Calculations were performed with default parameters and a temperature setting of 37°C for all organisms. The latter allowed us to detect the effect of the organismal nucleotide composition on the mRNA structure and stability. Sequences in a sliding window of 50 bases were folded (51), and their characteristics were calculated. We have also checked other sliding windows (100 and 150 bases), observing that they give similar qualitative outputs. The choice of 50-bases window is justified by the previous knowledge that most known functionally important secondary structures are small and local (48), and structures >50 bases would not normally be formed in actively translating mRNAs (48). We used dicodon-shuffle program by Katz and Burge (48) for obtaining control sets of reshuffled mRNA sequences. The base pairing pattern [base pair frequencies at three Phases (52) of mRNA sequence], folding energy of folded mRNAs and their correlations with OGT of corresponding organisms were examined. The comparison of these features between Archaea and Bacteria was also performed. To purify signal, i.e. to focus exclusively around an effect of the local mRNA structure, each quantity is also represented as a ratio between the natural sequence signal and the signal from dicodon-shuffled sequences (dShfld). The purine load in the loop and stem regions of the predicted mRNA secondary structures was also analyzed (21).

#### Amino acid and dipeptide composition and OGT correlation

Amino acid *Z*-scored predictors of the OGT (43) were derived for Archaeal and Bacterial proteomes. Additionally, we analyzed the groups of amino acids according to their physical chemical properties [charged (D, E, K, R), hydrophobic (C, F, I, L, M, P, V, W) and polar (A, G, H, N, Q, S, T, Y)]. The dipeptide classes for above residues types and their correlations with OGT were examined separately for Archaea and Bacteria. The dipeptide frequencies were normalized by the individual amino acid frequencies in order to exclude the compositional bias.

## RESULTS

In order to achieve our goal in understanding compositional and sequence signals of evolution and adaptation of DNA, RNA and protein macromolecules, we pursue the following strategy in the analysis. First, wherever it is possible we single out compositional biases existing in these molecules. Then, we establish possible connections between detected biases and mechanisms of adaptation. Specifically, we discuss nucleotide, dinucleotide, amino acid and dipeptide biases, their correlations with different environmental factors, and their potential role in determining and tuning molecular mechanisms of stability and adaptation in corresponding macromolecules. We also seek to understand how biases in one type of molecules can affect or can be affected by the demands on the sequences/structures of others. Finally, we explore causal relationships between them in light of their evolutionary history and phylogeny. To this end, we analyze the difference in compositional/sequence biases between Archaea and Bacteria in conjunction with their mechanisms of adaptation.

### Nucleotide compositions of DNA and RNA

ncDNA has significantly higher adenine and thymine (A + T) content in Archaea (Table 2 and Supplementary File S9), hinting to the role of nucleotide composition in discriminating coding and ncDNA of Archaea; the same mechanism exists in eukaryotes. Deviations of nucleotide contents from even fractions differ between Archaea and Bacteria: A and C is significantly deviated in Archaea, whereas in Bacteria the composition of T and G nucleotides is skewed (Table 2). There is an increased (G + C) load in t- and rRNA of Archaea and tRNA of Bacteria. Bacterial rRNA yields higher guanine load. Both guanine and cytosine loads of t- and rRNA correlate with OGT, and the correlation is stronger in Archaea than in Bacteria (Table 2, correlation coefficients and their significance levels are given in parentheses, *P*-values are in Supplementary File S8). The role of the (G + C) content in thermal adaptation of t- and rRNAs is further corroborated by folding simulations. The (G + C) content increases and energy per base pair decreases with temperature in stems of folded t- and rRNAs (Table 3). These data point to base-pairing interactions as an important contributor to thermal stabilization (53) of t- and rRNAs of prokaryotes; and it is stronger manifested in Archaea than in Bacteria.

**Table 2.** Nucleotide compositions and their OGT correlations in DNA and RNAs

Domain of life	Nucleotide	cDNA_Nat_	cDNA_NCB_	ncDNA	tRNA (*R*)	rRNA (*R*)
Archaea	A	28.45*	27.94**	30.73**	17.13** (–0.76**)	23.68 (–0.85**)
	T	23.89	24.44*	30.65**	18.17** (–0.89**)	19.24 (–0.88**)
	G	26.00	26.12*	19.30**	33.91** (0.84**)	32.08 (0.94**)
	C	21.66*	21.51**	19.31**	30.80** (0.84**)	24.99 (0.76**)
Bacteria	A	24.05	26.42**	26.57*	19.66 (–0.44**)	26.09 (–0.52**)
	T	22.68**	23.70**	26.61*	21.56 (–0.51**)	20.68 (–0.77**)
	G	27.50**	26.66**	23.41*	31.21 (0.53**)	31.10 (0.72**)
	C	25.77	23.23**	23.41*	27.58 (0.39*)	22.13 (0.60**)

The numbers represent the average frequencies of nucleotides in the corresponding parts of genomes, while the numbers in parentheses are correlation coefficients (*R*) of nucleotide frequencies with OGT. The most important biases and correlations are shown in bold font. The *P*-values for correlations (*H*_0_: correlation coefficient *R* = 0) and for the nucleotide composition *t*-tests (*H*_0_: mean frequency is 0.25) are shown in superscripts as significance levels: *P-value < 0.01, ** < 0.0001. Supplementary Files S8 and S9 list all correlations and composition tests. cDNA_NAT_, natural nucleotide composition in coding DNA; cDNA_NCB_, nucleotide composition in coding sequences with eliminated codon bias.

**Table 3.** OGT correlations in r- and t-RNA observed in folding simulations

Domain of life	RNA type	*R*((G + C), OGT) (*P*-values)	*R*(<*E*_bp_>, OGT) (*P*-values)
Archaea	rRNA	0.89 (<10^–22^)	–0.93 (1.1 × 10^–20^)
	tRNA	0.84 (1.84 × 10^–13^)	–0.71 (3.34 × 10^–8^)
Bacteria	rRNA	0.73 (1.38 × 10^–14^)	–0.66 (1 × 10^–11^)
	tRNA	0.53 (3.1 × 10^–7^)	–0.51 (9.1 × 10^–7^)

*R*((G + C), OGT), correlation coefficient between the (G + C) content and the OGT; *R*(<*E*_bp_>, OGT), correlation coefficient between the averaged per base pair free energy of RNA folding and OGT.

### Dinucleotide composition of nucleic acid sequences

Dinucleotide contrasts (DCTs) show the ratio of observed dinucleotide frequencies to the expected ones given the composition of individual nucleotides. Coupling of the same nucleotides (Table 1) is preferred in ncDNA sequences in Archaea (ApA/TpT and CpC/GpG) and Bacteria (ApA/TpT). ApA and CpC dinucleotides are prevalent in t- and rRNA of Archaea and rRNA of Bacteria. Other outstanding contrasts in Archaea are found in ncDNA (GpT/ApC) and tRNA (TpC, ApC and TpG). In Bacteria, ApG and TpC are prevalent in tRNA (Table 1), while GpC is preferred in both non-coding and coding sequences. In coding sequences of Archaea, there is no preference for coupling of similar nucleotides, while in Bacteria it is found for ApA and TpT (Table 1).

In both coding and ncDNA sequences of Archaea there is a clear excess of complementary ApG and CpT dinucleotides, which is persistent after elimination of the codon bias and reshuffling of amino acid sequence (Table 1). In Bacteria, the excess of GpG dinucleotides in non-coding and coding sequences is most pronounced (Table 1), provided by the codon bias in coding sequences. Pairing of similar nucleotides is highly correlated with OGT in tRNA (ApA, TpT, GpG and CpC) and rRNA (ApA, GpG and CpC) in Archaea. Correlation of the same nucleotide pairs is also found in Bacteria, though it is for fewer pairs and is weaker (tRNA: ApA; rRNA: ApA and CpC). In Archaeal t- and rRNA there is also strong correlation of TpA pairs with OGT (Table 1).

### Correlation between nucleotide frequencies and temperature in different codon positions

The first codon position in Archaea is characterized by high correlation of the ratio of natural to NCB frequencies of adenine with OGT [A_Nat/NCB_, *R* = 0.69, Table 1]. The second codon position reveals correlation of the thymine frequency with OGT (Table 1). The combination of thymine and guanine nucleotides is also correlated with OGT, however thymine is the major contributor (Table 1). The correlation of the guanine content with OGT is supported by the codon bias (Table 1). The combination of guanine with cytosine is also correlated with OGT in the second position of Archaeal sequences and is provided by the codon bias. The third position reveals strong selection against adenine and guanine in relation to OGT (Table 1). Bringing together (anti-)correlations with OGT in different codon positions, one can draw the optimal, from the point of view of thermal adaptation (Table 4), combined triplet as [A]_1_ [T,G]_2_ [non-(A,G)]_3_. Prevalence of thymine in the second codon position is linked to an excess of hydrophobes. Indeed, codons with thymine in the second position encode Ile, Leu, Met, Phe and Val. These are strongly hydrophobic residues and aromatic Phe, which can form many van der Waals contacts and contribute thus to the packing of the hydrophobic core. Noteworthy, elimination of the codon bias does not affect correlation of the thymine fraction with OGT (*R* = 0.55 in both native and NCB sequences). It shows that excess of thymine in the second codon position is a result of selection on the protein level. An apparent explanation for such selection is domination of the structure-based strategy in thermostabilization of Archaeal proteins. This strategy is characterized by the increased compactness of the hydrophobic core provided by the massive van der Waals contacts. The correlation of the combination of A and G with OGT is not provided by tuning of the codon bias, which points to adaptation on the other than DNA level.

**Table 4.** Generalized nucleotides and dinucleotides in different codon positions favorable for thermostability

Nucleotides correlated with OGT				
Domain of life	Codon position	1	2	3
Archaea	Nucleotide	A	T,G	Non-[A,G]
	Origin of the bias	Codon bias	T- amino acid	Against codon bias
			G-codon bias	
Bacteria	Nucleotide	Weak A	Weak T	Non-[A,G]
	Origin of the bias	Codon bias	Amino acid	Against codon bias

Dinucleotides in Purine (R) and Pyrimidine (Y) notation correlated with OGT				
Domain of life	Codon position	1-2	2-3	3-1
Archaea	Dinucleotide	RpR/YpY	Non-RpR/YpY	RpR/YpY
	Origin of the bias	Codon bias	Codon bias	Amino acid
Bacteria	Dinucleotide	RpR/YpY	Non-RpR/YpY	RpR/YpY
	Origin of the bias	Not codon bias	Codon bias	Amino acid

Part 1. Thermophilic-prone nucleotide biases: Columns 1, 2, 3 contain information on favorable nucleotides and origin of the bias in codon positions 1, 2, 3. Part 2. Thermophilic-prone dinucleotide biases: Columns 1-2, 2-3, 3-1 contain information on favorable nucleotides and origin of the bias in codon positions 1-2, 2-3, 3-1.

Bacterial coding DNA sequences yield less position-dependent correlations with OGT than Archaeal ones. The ratio of natural adenine frequency over the one for eliminated codon bias is weakly correlated with OGT (Table 1). There are also moderate correlations of thymine frequency and of the thymine and guanine combination with OGT in the second codon position (Table 1). The third codon position is characterized by selection against adenine provided by the codon bias (Table 1). However, the combination of adenine and guanine is correlated with OGT. It does not depend on the codon bias to a large extent (same as in Archaea), pointing to possible signal of adaptation on the level other than protein-coding DNA sequence. The generalized codon in Bacteria (Table 4) characterizing the thermophilic trends reads therefore: [weak A]_1_ [weak T]_2_ [non-(A,G)]_3_.

### The role of excessive (G + C) load in the third codon position in adaptation to aerobicity

In both Archaea and Bacteria, the average nucleotide composition in different codon positions is not affected by the codon bias to the large extent, except the third codon position in Bacteria. However, the compositional variance on the third position is essentially destroyed when there is no codon bias (standard deviation (SD) diminishes from >10% to <1%, see Supplementary File S5). The natural (G + C)_3_ load in Bacteria yields ∼9% excess in comparison with NCB sequences (*P*-value = 1.7*e* – 07, Supplementary File S6), while in Archaea there is no significant difference. We found that despite frequent involvement of G_3_ and C_3_ nucleotides into the G•C/C•G pairs, the (G + C)_3_ load does not play a crucial role in thermal stability of mRNA (52,54). Overall the difference in (G + C)_3_ load between Archea and Bacteria is insignificant. If we consider separate thermal groups, the only difference appears in mesophilic Bacteria (∼9.5% more in Bacteria, *P* = 0.09). However, when we take into account the oxygen tolerance factor, the difference in (G + C)_3_ load will become extremely pronounced between aerobic (70.13 in natural and 50.84 in NCB) and anaerobic (48.94 in natural and 50.46 in NCB) species (*P*-value = 2*e* – 09). Considering the lack of OGT correlation in protein-coding nucleotides (except thymine on the second codon position), one can conclude that increased (G + C)_3_ load is a result of the aerobic life style, rather than thermal adaptation (Supplementary Files S2 and S3). The comparison of aerobes and anaerobes in the group of mesophilic Bacteria corroborates this conclusion (Supplementary File S6). The difference is >20% of (G + C)_3_ content: 70.97 in aerobes and 50.51 in anaerobes (*P* = 0.000178). This adaptation is entirely driven by the codon bias, because if the codon bias is removed the (G + C)_3_ bias as well as the difference between aerobes and anaerobes will disappear (50.87% and 50.64%).

We also compared usage of codons with G or C in a third position with other codons (‘Codon Usage’ in Supplementary Files S3 and S6). There are three amino acids encoded by six codons (Leu, Arg, Ser); five, by four codons (Ala, Pro, Thr, Gly, Val); one, by three codons (Ile); nine, by two codons (Lys, Asn, Asp, Phe, Cys, Gln, Glu, His, Tyr); and two, by one codon (Met, Trp). Typically, half of the synonymous codons of each amino acid have G or C in a third codon position, others, A or T. It appeared that almost all of the codons with G and C in a third position have higher frequencies in aerobes compared to anaerobes, and in Bacteria than in Archaea (see ‘Codon Usage’ in Supplementary File S3). This observation holds for all codons of the ‘two-codon’ amino acids, and for majority of codons of the three-, four- and six-codon amino acids. The noticeable exception is significant suppression of AGA/AGG codons of the Arg (aerobes–anaerobes: −17.7/–12.5, *P*-values 4.46*e* – 8/5.8*e* – 8; Bacteria–Archaea: −17.4/–26.25, *P*-values 1.65*e* – 7/1.14*e* – 11). In lysine the codon AAA is suppressed in aerobes (difference −17.16, *P*-value 6.44*e* – 6), while the codon AAG is preferred. The AAG codon of lysine is favorable in aerobes (aerobes/anaerobes: 1.43), and another lysine’s codon AAA can be turned into AAG by only one mutation in the third codon position. Therefore, one can speculate that the demand for discriminating between Arg and Lys is the most probable cause for the suppression of AGA and AGG codons of arginine in aerobes. Lysine is thus preferred in aerobic adaptation over the arginine (Supplementary Files S3 and S6). The contribution of the amino acid in relation to thermophilic adaptation is not compromised, because of the similarity between physical–chemical characteristics of lysine and arginine from the point of view of thermostability. Overall, an excess of the G_3_ codons is always more pronounced in aerobes compared to anaerobes, which corroborates the conclusion that the (G + C)_3_ load is an indications of the aerobic style (55–58). The similar trend in the difference between the Bacteria and Archaea is a result of the domination of aerobic life style in the Bacteria in the analyzed dataset.

#### Correlation between dinucleotide frequencies in different codon positions and OGT

In order to analyze dinucleotides in different codon positions (1-2, 2-3, and 3-1) we used the DCT_Nat_, DCT_NCB_ (for positions 1-2 and 2-3), and shuffled (DCT_Shfld_, for position 3 and 1) sequences. We considered correlations of these contrasts with OGT. The most pronounced biases are found for dinucleotides in coding sequences of Archaea. The ApG dinucleotides show the strongest excess of dinucleotides in all positions of natural sequences compared to NCB and shuffled ones. In codon positions 1-2 and 2-3, this compositional peculiarity and its correlation with OGT are provided by the codon bias (Table 1). In position 3-1, high correlation of ApG frequencies with OGT is determined by the amino acid sequences, and it vanishes after amino acid reshuffling (Table 1). There is also correlation of the frequency of complementary CpT dinucleotide in position 3-1 with OGT, disappearing after reshuffling of amino acid sequences (Table 1). The most plausible role of ApG dinucleotides is a contribution to stabilization via base-stacking interactions between the purine rings (22). This conclusion is supported by the fact that excess of ApG dinucleotides is provided by the codon bias, manifesting thus adaptation on a DNA level. The complementary CpT dinucleotides indicate enrichment of anti-sense strand of double-stranded DNA (dsDNA) with the stabilizing ApG ones. The resulting mosaic of ApG stacking in sense and anti-sense strands can provide stabilization over the long distances without compromising flexibility of the dsDNA. Other overrepresented dinucleotides (regardless of the codon bias) correlated with OGT in positions 1-2 are GpC, CpC, TpA and CpT (Table 1). The frequencies of TpA and CpT dinucleotides are correlated with OGT in position 2-3, where the former is provided by the codon bias and the latter is not.

In purine (R) and pyrimidine (Y) notation, codon bias provides grouping (stacking) of similar nucleotides in position 1-2, which increases with OGT (Table 1). At the same time, codon bias works against such grouping in position 2-3 (Table 1). Amino acid sequence is responsible for the grouping of similar nucleotides and correlations with OGT in position 3-1 (Table 1). The resulting generalized thermophilic pattern of dinucleotides in Archaeal cDNA sequences reads: [RpR, YpY]_1-2_ [non-RpR, non-YpY]_2-3_ [RpR,YpY]_3-1_. Most of the compositional peculiarities found in Bacteria are not determined by the codon bias, yielding weak correlations with temperature (Table 1). The thermophilic pattern of dinucleotides in Bacteria (Table 4) is the same as Archaeal one. The difference with Archaea is that selection for RpR and YpY dinucleotides in position 1-2 is not determined by the codon bias in Bacteria (Tables 1 and 4).

#### Compositional and sequence biases observed in folding simulations of mRNA

Though RNA molecules have different overall structures, one may expect that there are some common mechanisms providing stability and function of the folded t-, r- and mRNAs. We have shown above that the (G + C) content apparently contributes to the stability of the t- and rRNA (36,59), and not to the stability of coding DNA (54,59,60). It is manifested in correlation of the former with OGT (Table 2), and the anti-correlation of the base-pairing energy in the folded structures with OGT (Table 3). Nucleotide compositions in coding DNA (hence, in mRNA as well) do not correlate with OGT. The mRNA case is of special interest, because redundancy of genetic code endues the nucleotide sequence with a potential to satisfy requirements for DNA, mRNA and protein stability. For example, it has been hypothesized (61) that the optimization of the base-pairing in the mRNA contributes to formation of stem fragments. Authors claimed that there is a corresponding periodic pattern of the mRNA secondary structure in human and mouse, which is determined chiefly by the selection that operates on the third codon position (52). An opposing opinion suggests that the secondary structure in mRNA interferes with translation and, therefore, should be avoided (62). Overall, three scenario of the relation between mRNA and proteins has been considered earlier (52): (i) the biases are determined by the demands on protein stability; (ii) mRNA stability is the major determinant of sequence biases; (iii) complete independence of the sequence biases related to mechanisms of stability on each level. We have computationally folded the mRNA sequences from 244 prokaryotic genomes and analyzed their characteristics (Table 5). All the parameters are reported as relative ones where the signal in native sequence is normalized by the value obtained for dicodon shuffled ones (48). The dicodon shuffling randomizes mRNA sequence while preserving the native protein sequence, native codon usage and native dinucleotide composition. Therefore, it allows one to select out peculiarities of mRNA caused by the demands on its stability and function from the others related to the coding DNA and proteins.

**Table 5.** Compositional and dicodon signals of mRNA adaptation purified by the dicodon shuffling

Characteristic	Archaea	Bacteria
The most and least (after the comma) frequent pairs in stems of mRNA	Mesophiles	
	Phase I: C2•3G, G2•3U	Phase I: G3•2U, U3•2G
	Phase II: C3•1G, G1•3U	Phase II: A1•3U, G1•3U
	Phase III: A3•3U, U3•3G	Phase III: G3•3C, U3•3G

	Thermophiles	
	Phase I: G3•2C, G2•3U	Phase I: C2•3G, G2•3U
	Phase II: C2•2G, G1•3U	Phase II: C3•1G, G1•3U
	Phase III: A3•3U, U3•3G	Phase III: U3•3A, U3•3G
Correlations with OGT of		
Segment energy, <*E*_sg_>	*R* = –0.71	*R* = –0.39
Energy per base pair, <*E*_bp_>	*R* = –0.73	*R* = –0.54
The most signification correlations with OGT	Phase I: U3•2G, *R* = 0.77	(U2•1G)_Nat_, PIII, *R* = 0.46
	Phase I: G2•3U, *R* = 0.71	(G1•2U)_Nat_, PIII, *R* = 0.45
	Phase I: All pairs, *R* = 0.7	(U2•1G)_dShfld_, PIII, *R* = 0.45
		(G1•2U)_dShfld_, PIII, *R* = 0.41

Phases I, II and III correspond to positioning of triplets where, respectively, first, second and third nucleotides are complementary. All signals (except OGT correlations in Bacteria) are normalized by corresponding values for control sequences after the dicodon shuffling. See explanations of abbreviations in the Materials and methods section.

Structure of stems in folded mRNA is characterized by phases, which show relative location of triplets in opposite strands of the stem. Specifically, Phases I, II and III correspond to positioning of triplets where, respectively, first, second and third nucleotides are complementary. The most and the least frequent pairs in all phases of the dsDNA (see Materials and methods section) are considered. Phases I and II are similar to each other, yielding C•G, G•C, G•U, U•G, A•U and U•A (Table 5). Overall, the Archaea and thermophilic Bacteria have similar trends in most and least frequent pairs. Mesophilic Bacteria stands out with A1•3U and G3•3C most frequent pairs in Phases II and III, respectively. The major contribution from the three pairing phases (52) to the total amount of stem pairs is provided by Phase III and nucleotides in codon position 3 are most frequent in stem pairs (Table 5 and Supplementary Table S1). OGT correlations highlight the contribution from the GU wobble pair to the thermostability of achaeal mRNA structures (Table 5 and Supplementary Table S1). We found the highest OGT correlation for the Archaeal pairs U3•2G (Table 5), confirming importance of the G•U wobble pairs. The stronger effect for Archaeal rather than for Bacterial mRNA is possibly a relic of ancient RNA world where RNA was the carrier of genetic information and harsh environmental conditions demanded its increased stability.

Table 6 shows that purine load (the contents of A + G, R/Y, ApG) is larger in the loop regions than in the stems. In Archaea this difference is slightly more pronounced than in Bacteria (Supplementary Table S2). The purine load is also in good positive correlation with OGT for the loops, not for stems. However, the amount of ApG dinucleotides is correlated with OGT in both loops and stems. For synonymous codons, the fractions of purine-rich codons (e.g. GGR versus GGY for glycine and AGR versus CGY for arginine) are correlated with OGT in both loop and stem regions (slightly stronger in loops). For non-synonymous codons, however, amount of purine-rich codons (GAR for glutamate) is correlated with OGT in both loops and stems, while fractions of purine-rich AAR codons for lysine do not correlate with OGT (Table 6). While Forsdyke *et al.* (21) reported increase of the purine content and its positive correlation with OGT in loops, we found that fraction of purine reach synonymous codons is correlated with OGT in stems as well. At the same time, the amount of non-synonymous purine-rich codons of lysine is not correlated with OGT in both loop and stem regions. Additionally, the amount of ApG dinucleotides is correlated with OGT in both loops and stems (Table 6).

**Table 6.** Purine loading in loop and stem regions of folded mRNA and its OGT correlation

Feature	Loop		Stem		L-v-S p-value
	Mean contents	OGT correlation	Mean content	OGT correlation	
A + G	0.560	0.59**	0.500	–0.26*	<2.2*E* – 16
R/Y	1.299	0.61**	1.002	–0.26*	<2.2*E* – 16
ApG	0.061	0.79**	0.051	0.50**	0.0002
GGR (glycine)	0.027	0.62**	0.045	0.42**	1.2*E* – 17
GGY (glycine)	0.017	–0.05	0.079	–0.23*	1.0*E* – 42
AGR (arginine)	0.035	0.72**	0.022	0.56**	2.4*E* – 10
CGR (arginine)	0.018	–0.22	0.040	–0.24*	7.1*E* – 09
CGY (arginine)	0.016	–0.25*	0.037	–0.26*	6.2*E* – 10
GAR (glutamate)	0.060	0.71**	0.036	0.59**	<2.2*E* – 16
AAR (lysine)	0.086	0.22	0.017	0.11	<2.2*E* – 16
GAY (aspartate)	0.037	–0.17	0.040	–0.37**	0.0002

Feature, analyzed nucleotide, dinucleotide or amino acid; Loop and stem, information on mean content and OGT correlation of the above; L-v-S, a comparison between corresponding contents in the loop region and stem regions by Wilcoxon-tests, and *P*-values are shown in this column. Correlation coefficients with OGT are shown. *P-value < 0.01, **P-value < 0.0001.

#### Signals of thermophilic adaptation in protein sequences

It has been shown earlier (22) that environmental temperature directly affects amino acid composition of prokaryotic proteins, and IVYWREL combination can serve as a predictor of the OGT in prokaryotes. We use here a ‘*Z*-score’ predictor of OGT (43), which takes into account differences in variances of the proteomic frequencies of individual amino acids (Table 7). We have shown that the *Z*-score predictor properly corrects for these differences (43), better reflecting the contribution of the amino acid combinations to thermal adaptation. Separate predictors for Archaea and Bacteria (based on 33 and 99 proteomes with OGT’s spread over 5–100 and 10–85°C, respectively) yield the same general trend in the increase of hydrophobic and charged residues at the expense of polar ones (34), showing minor differences in presence of individual residues.

**Table 7.** Signals of thermophilic adaptation in protein sequences of Archaea and Bacteria

Correlation with OGT	Archaea	Bacteria
*Z*-scored predictor	ILVW Y DKR (*R* = 0.93)	IPV Y EKR (*R* = 0.89)
The most abundant residues in >70% (>60%) of all *Z*-scored predictors	VIWL Y ER	VPI Y ER(K)
Individual amino acids	+: L, W	+: E
	–: T, Q, D	
Types of amino acids	+: h	+: h, c
	–: p	–: p
Dipeptides	+: hp, ph	+: cc
	–: cp, pc	–: pc

+, increase of the amino acid (or amino acid type) fraction with OGT; –, decrease of the amino acid (or amino acid type) fraction with OGT. Capital letters are names of amino acids; h, p, c are hydrophobic, polar, charged types of amino acids. Correlation coefficients between the *Z*-scored thermostability predictors and OGT are given in parentheses.

We have shown earlier (22) that the amino acid bias working in thermal adaptation of proteins does not depend on the overall nucleotide composition of coding-DNA sequences. However, one can still expect that nucleotide compositions of particular codon positions and/or dinucleotide compositions of positions 1-2 and 2-3 in codons may be somehow linked to amino acid biases. We indeed found a significant difference between nucleotide load in individual codon positions of Archaeal and Bacterial coding DNA sequences (Table 1). First, Archaeal protein coding sequences yield a strong correlation between (T + G)_2_ load in the second codon position and OGT (*r* = 0.72). The corresponding non-codon biased sequences show comparable correlation (*r* = 0.68) pointing that (T + G) load reflects tuning of the amino acid composition in connection to thermal adaptation. The main contributor to this correlation is thymine (Table 1) that provides increase of strongly hydrophobic residues LMFIV (Supplementary File S1). The (T + G) load is much weaker correlated with OGT in Bacterial sequences (*r* = 0.36 for natural and *r* = 0.42 for NCB sequences). The correlation of the thymine load with OGT is 0.37 for both Natural (NAT) and Non-codon-biased (NCB) sequences. This observation agrees with the presumed prevalence of structure-based strategy of thermal adaptation in Archaeal proteins (29) contrary to sequence-based one in Bacterial proteomes. Specifically, there is a clear correlation between amounts of charged residues and the OGT in the set of Bacterial proteomes (Table 7), presumably reflecting the domination of the sequence-based strategy in thermophilic adaptation of Bacteria (29).

Finally, we checked if there exists any specific connection between the dipeptide composition of proteins and those of 3-1 dinucleotides. We found (data not shown) that the strongest correlations between dinucleotides 3-1 and dipeptides exist mostly for the dinucleotides with guanine in position 3. The most frequent pair of amino acids contains methionine (Met, encoded exclusively by the AUG codon) as the first and any polar/charged amino acid as the second one. The preference of polar/charged amino acid to be the next to Met can be explained by the typically surface location of the protein N-termini and its role as a signal for ubiquitination (63).

## DISCUSSION

We will make a brief overview of the most pronounced biases and causal relationships in DNA, RNA and proteins. The major signal in nucleotide compositions, specifically (G + C) load, is related to thermal stabilization of t- and rRNA (36,54,55,59,60) and to discrimination between the coding and non-coding sequences in Archaea (Table 2). Comparative analysis of nucleotide and amino acid compositions in relation to thermophilic adaptation prompts to conclude that the (G + C) content does not contribute to thermostabilization of coding DNA (18,22,36,54,59), as well as it does not affect amino acid composition and its thermophilic trends (15,22). When separate codon positions are considered for coding DNA, the only compositional bias observed for nucleotide compositions is an excess of (G + C) load in the third codon position in Bacteria. There is a significant excess of guanine and cytosine nucleotides (29.2% and 32.82%) compared to the NCB nucleotide contents (26.09% and 24.66%). A plausible explanation for this bias is the role of the (G + C)_3_ load in the adaptation to the aerobic life style, which dominates in Bacteria. The (G + C)_3_ load can be advantageous for several complementary reasons. Indeed, additional G•C base pairing will contribute to the stability of double helix of DNA and stem regions of RNA molecules. Moreover, G_3_ bases can work as scavengers of oxidizing agents providing protection for G bases in other codon positions (56–58). At the same time, this bias would not lead to any changes in amino acid composition, leaving protein structure and stability intact (55).

In this work we considered compositional and sequence biases in proteins in relation to those in corresponding nucleic acid sequences and to the phylogeny of species (Archaea or Bacteria). OGT correlations of nucleotides in different codon positions are similar in Archaea and Bacteria, but higher in the former (Table 1). The optimal from the point of view of thermophilic adaptation (i.e. most correlated with OGT) codon reads A_1_ [T,G]_2_ non-[A,G]_3_ in Archaea, but [weak A]_1_ [weak T]_2_ non-[A,G]_3_ in Bacteria (Table 4). Codon bias supports the strong correlation between the amount of Adenine and OGT in the first codon position in Archaea, same but weaker correlation in Bacteria. While we showed earlier (22) that amino acid thermophilic trend is not determined by the overall nucleotide composition, consideration of separate codon positions reveals an interesting link between positional nucleotide frequencies and amino acid composition in relation to domain of Life. Specifically, prevalence of the nucleotide thymine in the second codon position and its strong correlation with OGT in Archaea is apparently a result of the demand on enrichement of Archaeal proteins with hydrophobic residues (Table 7). Noteworthy, a massive increase of van der Waals interactions (24,64) was found to be the cornerstone of the structure-based evolutionary strategy of protein thermostability in ancient species (29). In Bacterial proteins, however, there is an increase of the fractions of charged residues with OGT (Table 7). Thus, we observe a transitions from structure-based evolutionary strategy of protein themostability in Archaea to sequence-based one in Bacteria and corresponding nucleotide and protein compositional biases (Table 7) underlying these strategies (29). The third codon position is characterized by the selection against adenine and guanine in Archaea and against adenine in Bacteria is a result of this bias.

The most pronounced dinucleotide compositional biases are excess of homo-dinucleotides and its correlation with OGT in ncDNA, tRNA and rRNA. It is presumably a relic of ancient primitive homo(poly)nucleotides from which life started. Overrepresentation of the complementary ApG/CpT dinucleotides points to their contribution to DNA/RNA stability via strong base-stacking interactions (65,66). The consideration of dinucleotide biases in purine (R) and pyrimidine (Y) notation also reveals an interesting picture. Overall, for both Archaea and Bacteria the signature for thermophilic dinucleotides reads RpR/YpY, non-(RpR/YpY), RpR/YpY for codon positions 1-2, 2-3 and 3-1, respectively (Table 4). The major difference between Archaea and Bacteria in this case is that the dinucleotide bias in positions 1-2 is caused by demands on nucleotide sequence level (and provided by the codon bias) in Archaea, while some other factors work in Bacteria. The preference for homodinucleotides in positions 1-2 and 3-1 can be a possible reason for avoiding homodinucleotides in positions 2-3, because heterodinucleotides can contribute to DNA flexibility tuning. Indeed, it has been shown that given an average flexibility F of homodinucleotides (either RpR or YpY), the flexibilities of the heterodinucleotides YpR and RpY are 2F and 0.5F, respectively (67). The codon positions 2-3 are most versatile in terms of the relationship between the nucleotide and protein sequences. Therefore, presence of heteronucleotides in the codon position 2-3 makes it possible to adjust flexibility of the nucleotide sequence without changing physical–chemical characteristics of the encoded amino acid residue. Additionally, we found that the link between dipeptide and corresponding 3-1 dinucleotide frequencies is determined by the amino acid dipeptide. The dominating bias reads as Met followed by a polar or charged residue.

On the RNA level, simple consideration of the nucleotide composition suggests to discriminate the tRNA and rRNA molecules from the mRNA. Indeed, based on the correlation with OGT we can conclude that (G + C) content is important for providing stability of tRNA and rRNA molecules, but not for mRNA. The role of the (G + C) content was further confirmed by folding simulations (Table 3). We also found other complementary signals in mRNA sequences related to its structure, stability and function. Archaea and Bacteria yield similar trends in the most and least frequent nucleotide pairs, and the third codon position makes a major contribution to the base-pairing mechanism of stem stabilization. Specifically, in addition to Watson–Crick pairing, the G•U wobble pairs sufficiently contribute to the thermal stability of the Archaeal mRNA in agreement with earlier observations (68,69). Overall, it results in moderate correlation of the segment energy and energy per base pair of the folded mRNA (Table 5). Folding simulations performed in this work also allowed us to analyze peculiarities of the nucleotide contents and structural contacts in stem and loop regions of folded mRNA. Purine load in loop regions is higher than in stems, and this effect is slightly stronger in Archaea than in Bacteria (Table 6). Purine load in loops also correlates with OGT, not in stems. This correlation was observed earlier by Forsdyke *et al.* (21), and it was described as ‘polite purine load of the loop regions’ that prevents undesired mRNA–mRNA single-strand interactions. The authors concluded that increased purine load can affect the codon choice and, consequently the amino acid composition (21). Our data [as well as careful analysis of the original data in (21)] does not support the latter claim. There is indeed a correlation between the fraction of some purine-rich codons (GGR(Gly), AGR(Arg) and GAR(Glu)) and OGT (Table 6). However, the fraction of purine-rich non-synonymous codons AAR (Lys) is not correlated with OGT (Table 6). Moreover, these codons are synonymous, and cannot directly affect the amino acid composition. It has been shown earlier (22,70) that increase of Glu and Arg fractions is a trend in protein thermophilic adaptation. At the same time, purine load remains high after elimination of the codon bias, pointing to the amino acid composition as the most probable cause for the former (22). All the above prompts one to conclude that in mutual tuning of nucleotide and amino acid compositions, the purine load does not dominate and determine biases in amino acid composition, if not opposite. The analysis of purine load shows that it correlates with OGT in both stems and loops (Table 6) in case of purine-rich codons GGR(Gly), AGR(Arg) and GAR(Glu). Further, the amount of ApG dinucleotides strongly correlates with OGT in both loops and stems (Table 6), and ApG provides a strong base stacking important for thermal stability of nucleic acid sequences (22,65). Purine load, therefore, is apparently a determinant of the base-stacking mechanism in mRNA and/or DNA thermal stability as well as a result of the thermophilic amino acid composition trend.

Prokaryotes thrive under the temperature interval spanning over hundred degrees, and they represent two major life styles—aerobic and anaerobic. Analysis of complete Archaeal and Bacterial genomes unraveled compositional and sequence signals related to molecular mechanisms of stability and adaptation unaffected by selective sequencing or by the comparison of orthologs. Overall, codon bias works stronger in Archaea and is mostly utilized in thermophilic adaptation of nucleic acids. It apparently reflects longer evolutionary history of Archaea, which presumably started close to the origin of life in hot conditions (29). The codon bias and amino acid sequences (dipeptide composition) work in accord for supporting enrichment of the nucleotide sequences with ApG/CpT dinucleotides—determinants of the base-stacking mechanism of the nucleic acid stability (65–67). We also found that the second codon position reveals a strong link between the nucleotide and amino acid compositions. Specifically, excess of thymine in this position is a result of a demand on the enrichment of Archaeal proteins with hydrophobic amino acids. The third codon position in Bacteria is the only case where codon bias is detectable already on the level of pure composition. We found that the (G + C)_3_ load is related to aerobic life style dominating in Bacteria. From the point of view of thermophilic adaptation, codon bias works against G in the third codon position in both Archaea and Bacteria. It supports thus a specific role of the third codon position in discriminating between adaptations to temperature and aerobic life style. Finally, we obtained an interesting and complex picture of the relationship between the nucleotide composition (purine load) and amino acid composition (selection between Arg and Lys) in relation to thermal adaptation, aerobicity and phylogeny. Specifically, purine load in both Archaea and Bacteria is a result of the ‘from both end of hydrophobicity scale’ trend in thermal adaptation of proteins (34) reflected in the IVYWREL predictor of the OGT (22). According to this predictor, Arg is a preferred amino acid for thermal adaptation, though Lys is the next candidate, present in top most correlated predictors as well (22,43). In particular, selection for Lys over the Arg in some species was shown to be important for the entropic mechanism of protein thermostabilization (70). However, overall preference for Arg over the Lys in case of thermal adaptation is well manifested in the excess of the purine-rich (AGR) codons of Arg at the expense of purine-rich ones of Lys (AAR). At the same time, discrimination between Arg and Lys works in opposite direction in aerobicity where Arg codons AGA and AGG are suppressed in favor of the purine-rich AAA and AAG of Lys. It thus reflect a preference for Lys over Arg in aerobes compared to anaerobes in particular and in Bacteria versus Archaea in general, preserving at the same time high purine load necessary for providing a base-stacking in corresponding nucleic acids (22,53,65). An intricate connection between the Lys/Arg and their codons in relation to thermal adaptation and aerobicity exemplifies how selection can work on nucleic acids and protein simultaneously in response to demands of different environments. Obviously, the whole picture of molecular mechanisms of adaptation and relations between them is far from being complete. Consideration of other environmental factors such as salinity, pressure, etc. will help to unravel new mechanisms of stability, their sequence/structure determinants, and to understand tradeoffs that Nature embraced *en route* of the evolution and adaptation.

## SUPPLEMENTARY DATA

Supplementary Data are available at NAR Online.

## FUNDING

Funding for Open access charges: Functional Genomics Program (FUGE II), Norwegian Research Council.

*Conflict of interest statement*. None declared.

## Supplementary Material

Supplementary Data
