# Supplementary material for: Molecular mechanisms of adaptation emerging from the physics and evolution of nucleic acids and proteins
Source: Nucleic Acids Res. 2013 Dec 25;42(5):2879–92. doi: 10.1093/nar/gkt1336 (PMC3950714; doi:10.1093/nar/gkt1336)
Supplement: Supplementary Data [file supp_gkt1336_nar-02158-n-2013-File002.pdf]

Table S1. Comparison of pairing pattern between Archaea and Bacteria in the stem region of the predicted mRNA secondary structures

Characteristic	**Archaea**	**Bacteria**
**Natural signal (Nat)**		
The most and least frequent pairs in stem regions of mRNA secondary structure	Mesophiles:<br>Phase 1: G1:1C, U3:2G<br>Phase 2: G1:3C, U1:3G<br>Phase 3: G1:2C, U1:2G<br>Thermophiles:<br>Phase 1: G3:2C, G2:3U<br>Phase 2: G1:3C, U1:3G<br>Phase 3: G1:2C, U1:2G	Mesophiles:<br>Phase 1: G1:1C, U3:2G<br>Phase 2: G1:3C, U1:3G<br>Phase 3: G3:3C, U1:2G<br>Thermophiles:<br>Phase 1: G1:1C, U3:2G<br>Phase 2: G1:3C, U1:3G<br>Phase 3: G3:3C, U1:2G
Contributions from the three pairing Phases to the stem pairs	Mesophiles:<br>Phase 3 > Phase 2 > Phase 1<br>Thermophiles:<br>Phase 3 > Phase 2 > Phase 1	Mesophiles:<br>Phase 3 > Phase 2 > Phase 1<br>Thermophiles:<br>Phase 2 > Phase 3 > Phase 1
The most and least frequent nucleotide involved in pairing	Mesophiles: C3, U1<br>Thermophiles: C3, U1	Mesophiles: C3, U1<br>Thermophiles: C3, U1
Contributions from the three codon sites to the stem pairs	Mesophiles:<br>Site 3 > Site 1 > Site 2<br>Thermophiles:<br>Site 3 > Site 1 > Site 2	Mesophiles:<br>Site 3 > Site 1 > Site 2<br>Thermophiles:<br>Site 3 > Site 1 > Site 2
Features in signification correlation with OGT	Phase 1: U2:3G (R = +0.56)<br>Phase 2: U1:3G (R = +0.57)<br>Phase 3: C2:1G (R = -0.50)	None
**Purified signal (Nat/dShufl)**		
The most and least frequent pairs in stem regions of mRNA secondary structure	Mesophiles:<br>Phase 1: C2:3G, G2:3U<br>Phase 2: C3:1G, G1:3U<br>Phase 3: A3:3U, U3:3G<br>Thermophiles:<br>Phase 1: G3:2C, G2:3U<br>Phase 2: C2:2G, G1:3U<br>Phase 3: A3:3U, U3:3G	Mesophiles:<br>Phase 1: G3:2U, U3:2G<br>Phase 2: A1:3U, G1:3U<br>Phase 3: G3:3C, U3:3G<br>Thermophiles:<br>Phase 1: C2:3G, G2:3U<br>Phase 2: C3:1G, G1:3U<br>Phase 3: U3:3A, U3:3G
Contributions from the three pairing Phases to the stem pairs	Mesophiles:<br>Phase 3 > Phase 2 > Phase 1<br>Thermophiles:<br>Phase 3 > Phase 2 > Phase 1	Mesophiles:<br>Phase 3 > Phase 2 > Phase 1<br>Thermophiles:<br>Phase 3 > Phase 1 > Phase 2
The most and least frequent nucleotide involved in pairing	Mesophiles: C3, A2<br>Thermophiles: C3, U3	Mesophiles: A1, U3<br>Thermophiles: A3, U3
Contributions from the three codon sites to the stem pairs	Mesophiles:<br>Site 3 > Site 1 > Site 2<br>Thermophiles:<br>Site 3 > Site 1 > Site 2	Mesophiles:<br>Site 3 > Site 2 > Site 1<br>Thermophiles:<br>Site 3 > Site 2 > Site 1
Features in signification correlation with OGT	Phase 1: U3:2G (R = +0.76)<br>Phase 2: U3:1G (R = +0.60)<br>Phase 3: None	None

Table S2. Purine loading in loop and stem regions of folded mRNA of archaea and bacteria, and its OGT correlation.

Feature	Loop		Stem		Comparison
	Mean Content	OGT	Mean	OGT	p-value
**Archaea**					
A+G	0.596	0.46*	0.500	-0.29	4.9E-27
R/Y	1.494	0.44*	1.001	-0.29	4.9E-27
ApG	0.073	0.83**	0.059	0.62**	0.0007
GGR(glycine)	0.032	0.44*	0.052	0.16	1.5E-14
GGY(glycine)	0.015	0.18	0.064	0.16	3.4E-26
AGR(arginine)	0.046	0.68**	0.028	0.55**	9.2E-11
CGR(arginine)	0.016	-0.28	0.032	-0.31	0.0024
CGY(arginine)	0.012	-0.10	0.026	-0.12	0.0010
GAR(glutamate)	0.069	0.60**	0.044	0.48*	4.2E-18
AAR(lysine)	0.096	-0.03	0.018	0.01	2.2E-23
GAY(aspartic)	0.038	-0.27	0.042	-0.63**	0.0158
**Bacteria**					
A+G	0.539	0.48**	0.501	0.08	4.6E-12
R/Y	1.188	0.51**	1.002	0.08	4.6E-12
ApG	0.054	0.58**	0.046	0.22	0.0020
GGR(glycine)	0.025	0.69**	0.041	0.46**	3.4E-10
GGY(glycine)	0.018	-0.02	0.088	-0.25	6.9E-28
AGR(arginine)	0.028	0.58**	0.019	0.37*	8.5E-08
CGR(arginine)	0.020	-0.07	0.044	-0.08	2.5E-07
CGY(arginine)	0.018	-0.16	0.043	-0.18	9.8E-09
GAR(glutamic)	0.054	0.70**	0.032	0.51**	<2.2E-16
AAR(lysine)	0.081	0.30*	0.017	0.18	<2.2E-16
GAY(aspartic)	0.037	-0.17	0.040	-0.33*	0.0046
**Both Archaea and Bacteria**					
A+G	0.560	0.59**	0.500	-0.26*	<2.2E-16
R/Y	1.299	0.61**	1.002	-0.26*	<2.2E-16
ApG	0.061	0.79**	0.051	0.50**	0.0002
GGR(glycine)	0.027	0.62**	0.045	0.42**	1.2E-17
GGY(glycine)	0.017	-0.05	0.079	-0.23*	1.0E-42
AGR(arginine)	0.035	0.72**	0.022	0.56**	2.4E-10
CGR(arginine)	0.018	-0.22	0.040	-0.24*	7.1E-09
CGY(arginine)	0.016	-0.25*	0.037	-0.26*	6.2E-10
GAR(glutamic)	0.060	0.71**	0.036	0.59**	<2.2E-16
AAR(lysine)	0.086	0.22	0.017	0.11	<2.2E-16
GAY(aspartic)	0.037	-0.17	0.040	-0.37**	0.0002

‘OGT correlation’ column shows the correlation coefficient, with ‘*’ indicating p-value < 0.01 and ‘**’ indicating p-value < 0.0001. Comparison was performed between Loop region and Stem region by Wilcoxon-tests and p-values are shown in the last column.
