# Supplementary material for: Molecular mechanisms of adaptation emerging from the physics and evolution of nucleic acids and proteins
Source: Nucleic Acids Res. 2013 Dec 25;42(5):2879–92. doi: 10.1093/nar/gkt1336 (PMC3950714; doi:10.1093/nar/gkt1336)
Supplement: Supplementary Data [file supp_gkt1336_nar-02158-n-2013-File003.pdf]

Supplementary File 1

Position-specific nucleotide and dinucleotide compositions and their OGT correlations in Archaea and Bacteria

Table of Contents

Archaea: Nucleic composition ....

Archaea: Nucleic combination composition ....

Archaea: Dinucleotide composition ....

Bacteria: Nucleic composition ....

Bacteria: Nucleic combination composition ....

Bacteria: Dinucleotide composition ....

Bacteria: Dinucleotide combination composition ....

Nucleic composition comparison of DNA, RNA and ncDNA in Archaea .... [illegible]

Nucleic composition comparison of DNA, RNA and ncDNA in Bacteria .... [illegible]

Dinucleotide composition comparison of DNA, RNA and ncDNA in Archaea .... [illegible]

Dinucleotide composition comparison of DNA, RNA and ncDNA in Bacteria .... [illegible]

Correlation between OGT and nucleic acid composition of DNA, RNA and ncDNA in Archaea .... [illegible]

Correlation between OGT and nucleic acid composition of DNA, RNA and ncDNA in Bacteria .... [illegible]

Correlation between OGT and dinucleotide composition of DNA, RNA and ncDNA in Archaea .... [illegible]

Correlation between OGT and dinucleotide composition of DNA, RNA and ncDNA in Bacteria .... [illegible]

Archaea: Nucleic composition

	sk	base	codon	NatFreq	NCBFreq	NatNCBRatio	NatCorOGT	NCBCorOGT	NatNCBRatioCorOGT	aa
1	A	A	1	29.89	28.54	1.05	0.09	-0.15	0.69	RKNMSTI
3	A	T	1	16.57	17.20	0.96	0.08	0.21	-0.01	LFCSWY*
5	A	G	1	36.11	36.11	1.00	-0.03	-0.03	-0.22	ADEGV
7	A	C	1	17.43	18.15	0.96	-0.14	0.31	-0.36	LRPQH
9	A	A	2	30.66	30.67	1.00	-0.18	-0.18	0.06	KNDQEHY*
11	A	T	2	31.08	31.08	1.00	0.55	0.55	0.09	LMFIV
13	A	G	2	16.93	16.91	1.00	0.24	0.14	0.60	RCSGW*
15	A	C	2	21.33	21.34	1.00	-0.36	-0.28	-0.60	APST
17	A	A	3	24.80	24.62	1.01	0.02	0.70	-0.03	ALRKPQSETGIV*
19	A	T	3	24.04	25.03	0.96	-0.09	-0.66	-0.05	ALRNDFCPSTGHYIV
21	A	G	3	24.95	25.33	0.98	0.22	0.55	0.16	ALRKMPQSETGWV*
23	A	C	3	26.21	25.02	1.05	-0.10	-0.68	-0.05	ALRNDFCPSTGHYIV

Archaea: Nucleic combination composition

	sk	bases	codon	NatFreq	NatNCBRatio	NCBFreq	NatCorOGT	NCBCorOGT	NatNCBRatioCorOGT	aa
1	A	A+T	1	46.46	1.02	45.73	0.09	-0.08	0.68	LRKNMFCSTWYI*
3	A	A+G	1	66.00	1.02	64.65	0.20	-0.50	0.71	ARKNMDSETGIV
5	A	A+C	1	47.32	1.01	46.69	-0.05	-0.05	0.71	LRKNMPQSTHI
7	A	T+G	1	52.68	0.99	53.31	0.05	0.05	-0.02	ALDFCSEGWYV*
9	A	T+C	1	34.00	0.96	35.35	-0.20	0.50	-0.04	LRFCPQSWHY*
11	A	G+C	1	53.54	0.99	54.27	-0.09	0.08	0.00	ALRDPQEGHV
13	A	A+T	2	61.74	1.00	61.75	0.11	0.11	0.05	LKNMDFQEHYIV*
15	A	A+G	2	47.59	1.00	47.58	-0.05	-0.20	0.03	RKNDCQSEGWHY*
17	A	A+C	2	51.99	1.00	52.01	-0.72	-0.68	0.01	AKNDPQSETHY*
19	A	T+G	2	48.01	1.00	47.99	0.72	0.68	0.15	LRMFCSGWIV*
21	A	T+C	2	52.41	1.00	52.42	0.05	0.20	0.15	ALMFPSTIV
23	A	G+C	2	38.26	1.00	38.25	-0.11	-0.11	0.60	ARCPSTGW*
25	A	A+T	3	48.84	0.98	49.65	-0.04	0.04	0.03	ALRKNDFCPQSETGHYIV*
27	A	A+G	3	49.75	1.00	49.94	0.56	0.67	-0.08	ALRKMPQSETGWIV*
29	A	A+C	3	51.01	1.03	49.64	-0.32	0.02	-0.09	ALRKNDFCPQSETGHYIV*
31	A	T+G	3	48.99	0.97	50.36	0.32	-0.02	-0.09	ALRKNMDFCPQSETGWHYIV*
33	A	T+C	3	50.25	1.00	50.06	-0.56	-0.67	-0.11	ALRNDFCPSTGHYIV
35	A	G+C	3	51.16	1.02	50.35	0.04	-0.04	0.21	ALRKNMDFCPQSETGWHYIV*

Archaea: Dinucleotide composition

sk	base1	base2	positions	NatFreq	NCBFreq	ShufflNatFreq	NatNCBRatio	NatContrast	NCBContrast	ShufflNatContrast	NatNCBContrastRatio	
1	A	A	A	12	9.73	9.73	9.71	1.00	1.06	1.11	1.06	0.95
3	A	T	A	12	3.94	3.95	3.94	1.00	0.78	0.75	0.78	1.04
5	A	G	A	12	13.04	13.04	13.02	1.00	1.18	1.18	1.18	1.00
7	A	C	A	12	3.95	3.95	3.96	1.00	0.74	0.71	0.74	1.04
9	A	A	T	12	9.78	9.78	9.79	1.00	1.05	1.10	1.05	0.95
11	A	T	T	12	6.45	7.07	6.45	0.91	1.25	1.32	1.25	0.95
13	A	G	T	12	7.87	7.87	7.87	1.00	0.70	0.70	0.70	1.00
15	A	C	T	12	6.98	6.36	6.99	1.10	1.29	1.13	1.29	1.14
17	A	A	G	12	5.16	3.81	5.17	1.35	1.02	0.79	1.02	1.29
19	A	T	G	12	2.13	2.12	2.13	1.00	0.76	0.73	0.76	1.04
21	A	G	G	12	7.40	7.40	7.40	1.00	1.21	1.21	1.21	1.00
23	A	C	G	12	2.23	3.58	2.23	0.62	0.76	1.17	0.76	0.65
25	A	A	C	12	5.22	5.22	5.22	1.00	0.82	0.86	0.82	0.95
27	A	T	C	12	4.05	4.05	4.05	1.00	1.15	1.10	1.15	1.05
29	A	G	C	12	7.80	7.80	7.80	1.00	1.01	1.01	1.01	1.00
31	A	C	C	12	4.26	4.26	4.27	1.00	1.15	1.10	1.15	1.05
33	A	A	A	23	7.70	7.89	7.69	0.98	1.01	1.04	1.01	0.97
35	A	T	A	23	7.09	7.64	7.09	0.93	0.92	1.00	0.92	0.92
37	A	G	A	23	4.00	3.75	4.00	1.07	0.95	0.90	0.95	1.06
39	A	C	A	23	6.01	5.33	6.01	1.13	1.14	1.02	1.14	1.12
41	A	A	T	23	7.40	7.45	7.40	0.99	1.00	0.97	1.00	1.03
43	A	T	T	23	8.56	8.00	8.56	1.07	1.15	1.03	1.15	1.12
45	A	G	T	23	3.32	4.25	3.32	0.78	0.82	1.00	0.81	0.82
47	A	C	T	23	4.76	5.34	4.76	0.89	0.93	1.00	0.93	0.93
49	A	A	G	23	8.06	7.89	8.06	1.02	1.05	1.02	1.05	1.03
51	A	T	G	23	7.02	7.44	7.02	0.94	0.90	0.95	0.91	0.95
53	A	G	G	23	5.09	4.66	5.10	1.09	1.20	1.09	1.21	1.10
55	A	C	G	23	4.79	5.33	4.79	0.90	0.90	0.99	0.90	0.91
57	A	A	C	23	7.49	7.45	7.49	1.01	0.93	0.97	0.93	0.96
59	A	T	C	23	8.42	8.00	8.42	1.05	1.03	1.03	1.03	1.00
61	A	G	C	23	4.52	4.25	4.52	1.06	1.02	1.00	1.02	1.02
63	A	C	C	23	5.78	5.34	5.78	1.08	1.03	1.00	1.03	1.03
65	A	A	A	31	8.84	7.08	7.93	1.25	1.19	1.01	1.07	1.18
67	A	T	A	31	6.44	6.99	7.62	0.92	0.90	0.98	1.06	0.92
69	A	G	A	31	7.40	7.32	7.09	1.01	0.99	1.01	0.95	0.98
71	A	C	A	31	7.05	6.99	7.25	1.01	0.90	0.98	0.93	0.92
73	A	A	T	31	4.26	4.16	4.36	1.02	1.04	0.98	1.06	1.06
75	A	T	T	31	4.22	4.40	4.19	0.96	1.06	1.02	1.05	1.04
77	A	G	T	31	3.57	4.29	3.96	0.83	0.86	0.98	0.96	0.88
79	A	C	T	31	4.53	4.39	4.06	1.03	1.04	1.02	0.94	1.02
81	A	A	G	31	8.04	8.83	8.56	0.91	0.90	0.99	0.96	0.91
83	A	T	G	31	9.54	9.12	8.34	1.05	1.10	1.01	0.96	1.09
85	A	G	G	31	9.11	9.12	9.29	1.00	1.01	1.00	1.03	1.01
87	A	C	G	31	9.51	9.11	9.91	1.04	1.00	1.01	1.05	0.99
89	A	A	C	31	3.48	4.40	3.94	0.79	0.80	0.98	0.91	0.82
91	A	T	C	31	3.92	4.61	3.88	0.85	0.93	1.01	0.93	0.92
93	A	G	C	31	4.89	4.57	4.63	1.07	1.12	0.99	1.06	1.13
95	A	C	C	31	5.21	4.61	5.00	1.13	1.14	1.02	1.09	1.12

	NatFreqCorOGT	NCBFreqCorOGT	NatContrastCorOGT	NCBContrastCorOGT	ShufflNatContrastCorOGT	NatNCBContrastRatioCorOGT	aa
1	-0.01	-0.01	0.14	0.43	0.14	-0.66	KN
3	0.42	0.43	0.50	0.69	0.49	0.03	Y*
5	-0.31	-0.31	-0.27	-0.27	-0.26	-0.06	DE
7	-0.67	-0.67	-0.43	-0.72	-0.43	0.32	QH
9	-0.08	-0.08	-0.75	-0.44	-0.75	-0.66	MI
11	0.19	0.41	0.13	-0.25	0.14	0.20	LF
13	0.48	0.48	0.46	0.46	0.47	0.31	V
15	0.14	0.80	0.23	0.04	0.23	0.17	L
17	0.71	0.12	0.77	0.11	0.76	0.63	RS
19	-0.10	-0.10	-0.35	-0.34	-0.34	-0.09	CW*
21	-0.13	-0.13	-0.30	-0.22	-0.31	-0.59	G
23	-0.33	0.41	-0.48	0.71	-0.48	-0.56	R
25	-0.66	-0.66	-0.40	-0.34	-0.40	-0.56	T
27	-0.61	-0.45	-0.60	-0.35	-0.60	-0.40	S
29	-0.06	-0.06	0.55	0.44	0.54	0.60	A
31	0.27	0.27	0.37	0.37	0.37	0.37	P
33	-0.23	0.16	-0.51	0.43	-0.51	-0.53	KQE*
35	0.37	0.65	0.63	-0.28	0.64	0.65	LIV
37	0.16	0.26	0.02	0.08	0.01	0.00	RG*
39	-0.24	-0.28	-0.36	-0.69	-0.36	-0.28	APST
41	-0.22	-0.51	-0.49	-0.43	-0.49	-0.39	NDHY
43	-0.04	0.38	0.01	0.51	0.00	-0.12	LFIV
45	-0.12	-0.21	-0.19	-0.40	-0.19	-0.14	RCSG
47	0.15	-0.28	0.61	0.68	0.61	0.56	APST
49	0.51	0.16	0.69	0.37	0.69	0.53	KQE*
51	0.12	0.48	-0.52	-0.48	-0.52	-0.37	LMV
53	0.38	0.38	0.17	0.57	0.17	-0.01	RGW
55	-0.15	-0.28	-0.27	-0.52	-0.27	-0.22	APST
57	-0.05	-0.52	0.19	-0.46	0.19	0.35	NDHY
59	-0.13	0.39	-0.24	0.54	-0.24	-0.32	LFIV
61	-0.11	-0.20	-0.13	-0.37	-0.14	-0.06	RCSG
63	-0.08	-0.28	0.33	0.69	0.32	0.22	APST
65	-0.04	-0.08	-0.14	-0.48	0.30	-0.08	
67	0.10	-0.18	0.49	0.53	-0.02	0.47	
69	0.29	-0.12	-0.10	-0.49	-0.18	-0.02	
71	-0.11	-0.18	-0.27	0.52	-0.17	-0.36	
73	-0.09	0.40	-0.39	-0.43	0.00	-0.36	
75	-0.10	0.03	-0.09	0.32	0.04	-0.14	
77	0.22	0.54	-0.14	-0.19	0.26	-0.12	
79	0.34	0.03	0.60	0.32	0.05	0.59	
81	0.26	0.27	0.53	0.61	-0.02	0.50	
83	-0.22	-0.27	-0.37	-0.57	-0.11	-0.30	
85	0.25	0.22	0.05	0.45	0.00	-0.02	
87	-0.15	-0.28	-0.19	-0.60	-0.01	-0.14	
89	-0.14	0.45	-0.20	-0.06	0.20	-0.21	
91	-0.18	0.11	-0.01	-0.03	0.09	-0.02	
93	0.02	0.39	-0.04	0.05	-0.07	-0.06	
95	-0.12	0.12	0.09	0.01	-0.24	0.09	

Archaea: Dinucleotide combination composition

	sk	base1	base2	positions	NatFreq	NCBFreq	ShufflNatFreq	NatNCBRatio	NatContrast	NCBContrast	ShufflNatContrast	NatNCBContrastRatio
1	A	R	R	12	35.33	33.98	35.30	1.04	1.12	1.10	1.12	1.02
3	A	Y	R	12	12.25	13.60	12.26	0.90	0.76	0.81	0.76	0.94
5	A	R	Y	12	30.67	30.67	30.68	1.00	0.89	0.90	0.89	0.99
7	A	Y	Y	12	21.74	21.75	21.75	1.00	1.22	1.17	1.22	1.04
9	A	R	R	23	24.85	24.20	24.83	1.03	1.05	1.02	1.05	1.03
11	A	Y	R	23	24.90	25.75	24.92	0.97	0.95	0.98	0.96	0.97
13	A	R	Y	23	22.74	23.39	22.73	0.97	0.95	0.98	0.95	0.97
15	A	Y	Y	23	27.51	26.67	27.52	1.03	1.04	1.02	1.05	1.02
17	A	R	R	31	33.38	32.35	32.87	1.03	1.02	1.00	1.00	1.02
19	A	Y	R	31	32.54	32.21	33.11	1.01	0.98	1.00	1.00	0.98
21	A	R	Y	31	16.20	17.43	16.88	0.93	0.96	0.99	1.00	0.97
23	A	Y	Y	31	17.88	18.01	17.14	0.99	1.05	1.02	1.00	1.03

	NatFreqCorOGT	NCBFreqCorOGT	NatContrastCorOGT	NCBContrastCorOGT	ShufflNatContrastCorOGT	NatNCBContrastRatioCorOGT
1	0.22	-0.24	0.40	0.15	0.40	0.59
3	-0.39	0.26	-0.39	0.00	-0.39	-0.62
5	-0.19	-0.19	-0.36	-0.08	-0.36	-0.61
7	0.34	0.54	0.34	-0.05	0.34	0.64
9	0.47	0.51	0.20	0.65	0.20	-0.11
11	0.28	0.17	-0.21	-0.63	-0.20	0.08
13	-0.62	-0.72	-0.27	-0.67	-0.27	0.02
15	-0.21	0.20	0.28	0.66	0.27	-0.02
17	0.49	0.29	0.30	0.16	0.20	0.33
19	-0.53	-0.66	-0.33	-0.16	-0.26	-0.38
21	-0.03	0.62	-0.31	-0.16	0.11	-0.34
23	-0.18	0.14	0.35	0.17	-0.06	0.39

Bacteria: Nucleic composition

	sk	base	codon	NatFreq	NCBFreq	NatNCBRatio	NatCorOGT	NCBCorOGT	NatNCBRatioCorOGT	aa
2	B	A	1	24.45	25.23	0.97	0.13	0.06	0.28	RKNMSTI
4	B	T	1	15.50	16.46	0.94	-0.02	0.03	-0.04	LFCSWY*
6	B	G	1	36.84	36.84	1.00	0.02	0.02	0.01	ADEGV
8	B	C	1	23.21	21.48	1.08	-0.15	-0.16	-0.16	LRPQH
10	B	A	2	28.21	28.24	1.00	0.13	0.13	-0.12	KNDQEHY*
12	B	T	2	29.68	29.68	1.00	0.37	0.37	0.08	LMFIV
14	B	G	2	18.34	18.01	1.02	-0.06	-0.03	-0.24	RCSGW*
16	B	C	2	23.76	24.06	0.99	-0.32	-0.35	0.25	APST
18	B	A	3	17.04	24.60	0.69	0.13	0.76	0.10	ALRKPQSETGIV*
20	B	T	3	20.94	24.65	0.85	-0.09	-0.43	-0.07	ALRNDFCPSTGHYIV
22	B	G	3	29.20	26.09	1.12	0.04	0.14	0.03	ALRKMPQSETGWV*
24	B	C	3	32.82	24.66	1.33	-0.06	-0.43	-0.03	ALRNDFCPSTGHYIV

Bacteria: Nucleic combination composition

	sk	bases	codon	NatFreq	NatNCBRatio	NCBFreq	NatCorOGT	NCBCorOGT	NatNCBRatioCorOGT	aa
2	B	A+T	1	39.95	0.96	41.68	0.08	0.06	0.29	LRKNMFCSTWYI*
4	B	A+G	1	61.29	0.99	62.07	0.31	0.22	0.34	ARKNMDSETGIV
6	B	A+C	1	47.66	1.02	46.71	-0.01	-0.04	0.36	LRKNMPQSTHI
8	B	T+G	1	52.34	0.98	53.29	0.01	0.04	-0.05	ALDFCSEGWYV*
10	B	T+C	1	38.71	1.02	37.93	-0.31	-0.22	-0.04	LRFCPQSWHY*
12	B	G+C	1	60.05	1.03	58.32	-0.08	-0.06	0.10	ALRDPQEGHV
14	B	A+T	2	57.90	1.00	57.92	0.21	0.21	-0.13	LKNMDFQEHYIV*
16	B	A+G	2	46.56	1.01	46.25	0.22	0.28	-0.15	RKNDCQSEGWHY*
18	B	A+C	2	51.97	0.99	52.30	-0.36	-0.42	-0.16	AKNDPQSETHY*
20	B	T+G	2	48.03	1.01	47.70	0.36	0.42	0.07	LRMFCSGWIV*
22	B	T+C	2	53.44	0.99	53.75	-0.22	-0.28	0.07	ALMFPSTIV
24	B	G+C	2	42.10	1.00	42.08	-0.21	-0.21	-0.23	ARCPSTGW*
26	B	A+T	3	37.98	0.77	49.25	0.02	0.29	0.07	ALRKNDFCPQSETGHYIV*
28	B	A+G	3	46.23	0.91	50.69	0.60	0.43	0.11	ALRKMPQSETGWIV*
30	B	A+C	3	49.86	1.01	49.26	0.21	0.30	0.11	ALRKNDFCPQSETGHYIV*
32	B	T+G	3	50.14	0.99	50.74	-0.21	-0.30	-0.08	ALRKNMDFCPQSETGWHYIV*
34	B	T+C	3	53.77	1.09	49.31	-0.60	-0.43	-0.08	ALRNDFCPSTGHYIV
36	B	G+C	3	62.02	1.22	50.75	-0.02	-0.29	0.03	ALRKNMDFCPQSETGWHYIV*

Bacteria: Dinucleotide composition

	sk	base1	base2	positions	NatFreq	NCBFreq	ShufflNatFreq	NatNCBRatio	NatContrast	NCBContrast	ShufflNatContrast	NatNCBContrastRatio
2	B	A	A	12	7.93	7.93	7.93	1.00	1.15	1.11	1.15	1.04
4	B	T	A	12	3.00	3.03	3.00	0.99	0.69	0.65	0.69	1.06
6	B	G	A	12	11.52	11.52	11.52	1.00	1.11	1.11	1.11	1.00
8	B	C	A	12	5.76	5.76	5.76	1.00	0.88	0.95	0.88	0.93
10	B	A	T	12	8.07	8.07	8.07	1.00	1.11	1.08	1.11	1.03
12	B	T	T	12	6.65	7.30	6.66	0.91	1.45	1.49	1.45	0.97
14	B	G	T	12	7.36	7.36	7.36	1.00	0.67	0.67	0.67	1.00
16	B	C	T	12	7.61	6.96	7.60	1.09	1.10	1.09	1.10	1.01
18	B	A	G	12	3.16	3.94	3.16	0.80	0.70	0.87	0.71	0.80
20	B	T	G	12	2.31	2.29	2.31	1.01	0.81	0.77	0.81	1.05
22	B	G	G	12	7.74	7.74	7.73	1.00	1.15	1.17	1.14	0.98
24	B	C	G	12	5.13	4.05	5.13	1.27	1.21	1.05	1.21	1.15
26	B	A	C	12	5.29	5.29	5.29	1.00	0.91	0.87	0.91	1.05
28	B	T	C	12	3.53	3.84	3.53	0.92	0.96	0.97	0.96	0.99
30	B	G	C	12	10.22	10.22	10.22	1.00	1.17	1.15	1.17	1.02
32	B	C	C	12	4.71	4.71	4.71	1.00	0.85	0.91	0.85	0.93
34	B	A	A	23	7.19	7.27	7.19	0.99	1.50	1.05	1.50	1.43
36	B	T	A	23	3.86	7.26	3.86	0.53	0.76	0.99	0.76	0.77
38	B	G	A	23	2.13	4.06	2.13	0.52	0.68	0.92	0.68	0.74
40	B	C	A	23	3.85	6.01	3.85	0.64	0.95	1.02	0.95	0.93
42	B	A	T	23	6.61	6.85	6.61	0.96	1.12	0.98	1.12	1.14
44	B	T	T	23	6.69	7.43	6.69	0.90	1.08	1.01	1.08	1.07
46	B	G	T	23	3.83	4.36	3.82	0.88	1.00	0.98	1.00	1.02
48	B	C	T	23	3.81	6.02	3.82	0.63	0.77	1.01	0.77	0.76
50	B	A	G	23	7.33	7.28	7.33	1.01	0.89	0.99	0.89	0.90
52	B	T	G	23	10.19	7.57	10.19	1.35	1.18	0.98	1.18	1.20
54	B	G	G	23	4.36	5.22	4.36	0.84	0.81	1.11	0.81	0.73
56	B	C	G	23	7.31	6.02	7.31	1.21	1.05	0.96	1.05	1.09
58	B	A	C	23	7.08	6.84	7.08	1.04	0.76	0.98	0.76	0.78
60	B	T	C	23	8.94	7.43	8.94	1.20	0.92	1.01	0.92	0.91
62	B	G	C	23	8.02	4.37	8.03	1.84	1.33	0.98	1.33	1.36
64	B	C	C	23	8.78	6.02	8.78	1.46	1.13	1.01	1.13	1.12
66	B	A	A	31	5.07	6.20	4.75	0.82	1.22	1.00	1.14	1.22
68	B	T	A	31	4.79	6.11	5.74	0.78	0.93	0.98	1.12	0.95
70	B	G	A	31	6.29	6.63	6.65	0.95	0.88	1.01	0.93	0.87
72	B	C	A	31	8.13	6.12	7.32	1.33	1.01	0.98	0.91	1.03
74	B	A	T	31	2.80	3.94	2.98	0.71	1.06	0.97	1.13	1.09
76	B	T	T	31	4.19	4.18	3.62	1.00	1.29	1.03	1.11	1.25
78	B	G	T	31	3.57	4.20	4.23	0.85	0.79	0.98	0.94	0.81
80	B	C	T	31	4.97	4.18	4.67	1.19	0.98	1.03	0.92	0.95
82	B	A	G	31	5.66	9.06	5.90	0.62	0.90	1.00	0.94	0.90
84	B	T	G	31	7.88	9.17	7.28	0.86	1.02	1.01	0.94	1.01
86	B	G	G	31	10.59	9.50	11.08	1.11	0.98	0.99	1.03	0.99
88	B	C	G	31	12.78	9.18	12.57	1.39	1.06	1.01	1.04	1.05
90	B	A	C	31	3.31	5.27	3.41	0.63	0.84	1.00	0.86	0.84
92	B	T	C	31	4.16	5.26	4.30	0.79	0.86	0.99	0.88	0.87
94	B	G	C	31	8.77	5.74	7.23	1.53	1.29	1.02	1.07	1.26
96	B	C	C	31	7.05	5.26	8.27	1.34	0.92	0.99	1.09	0.93

	NatFreqCorOGT	NCBFreqCorOGT	NatContrastCorOGT	NCBContrastCorOGT	ShufflNatContrastCorOGT	NatNCBContrastRatioCorOGT	aa
2	0.14	0.14	-0.07	0.14	-0.07	-0.24	KN
4	0.34	0.38	0.35	0.67	0.35	0.04	Y*
6	0.54	0.54	0.38	0.38	0.38	0.12	DE
8	-0.57	-0.57	-0.50	-0.68	-0.50	0.18	QH
10	0.08	0.08	-0.34	-0.28	-0.34	-0.25	MI
12	-0.01	0.25	-0.13	-0.03	-0.12	-0.09	LF
14	0.40	0.40	0.30	0.30	0.30	-0.10	V
16	0.10	0.07	0.32	0.11	0.32	0.28	L
18	0.36	-0.23	0.38	-0.18	0.38	0.39	RS
20	-0.25	-0.30	-0.15	-0.38	-0.15	0.09	CW*
22	-0.01	-0.01	0.07	0.02	0.07	0.24	G
24	-0.20	0.11	-0.45	0.65	-0.44	-0.57	R
26	-0.44	-0.44	-0.13	0.00	-0.13	-0.34	T
28	-0.32	-0.55	0.00	-0.13	0.00	0.11	S
30	-0.27	-0.27	-0.46	-0.48	-0.46	-0.25	A
32	0.03	0.03	0.49	0.79	0.49	0.14	P
34	0.12	0.31	-0.12	0.42	-0.12	-0.17	KQE*
36	0.10	0.43	0.05	-0.49	0.05	0.08	LIV
38	0.51	0.07	0.38	0.09	0.38	0.41	RG*
40	-0.06	-0.35	-0.26	-0.74	-0.26	-0.20	APST
42	-0.16	-0.15	-0.32	-0.48	-0.32	-0.28	NDHY
44	0.08	0.33	0.44	0.60	0.44	0.36	LFIV
46	-0.30	-0.16	-0.34	-0.05	-0.34	-0.30	RCSG
48	-0.10	-0.35	0.27	0.46	0.27	0.21	APST
50	0.14	0.31	0.06	0.45	0.06	-0.13	KQE*
52	-0.04	0.00	-0.40	-0.48	-0.40	-0.19	LMV
54	0.32	0.01	0.52	0.02	0.52	0.48	RGW
56	-0.11	-0.35	-0.12	-0.16	-0.13	-0.10	APST
58	0.15	-0.15	0.42	-0.48	0.41	0.52	NDHY
60	0.03	0.32	0.08	0.59	0.08	-0.03	LFIV
62	-0.24	-0.15	-0.51	-0.04	-0.51	-0.53	RCSG
64	-0.07	-0.35	0.08	0.45	0.07	0.02	APST
66	0.23	0.19	0.34	0.46	-0.17	0.31	
68	-0.11	-0.03	-0.15	-0.38	-0.02	-0.13	
70	0.27	0.17	0.19	0.37	-0.01	0.13	
72	-0.08	-0.03	-0.13	-0.41	-0.13	-0.04	
74	-0.02	0.15	-0.36	-0.36	0.10	-0.35	
76	0.03	0.00	0.35	0.42	0.04	0.31	
78	-0.04	-0.04	-0.14	-0.37	0.03	-0.10	
80	-0.08	0.00	-0.02	0.41	0.11	-0.08	
82	0.13	0.18	0.08	0.16	0.10	0.06	
84	-0.14	-0.13	-0.23	-0.31	0.08	-0.20	
86	0.16	0.12	0.35	0.31	-0.02	0.33	
88	-0.09	-0.13	-0.11	-0.30	-0.20	-0.09	
90	-0.08	-0.13	-0.22	-0.50	-0.12	-0.18	
92	-0.09	-0.14	0.27	0.60	0.20	0.20	
94	-0.18	-0.23	-0.41	-0.62	-0.08	-0.34	
96	0.04	-0.13	0.32	0.60	0.06	0.24	

Bacteria: Dinucleotide combination composition

	sk	base1	base2	positions	NatFreq	NCBFreq	ShufflNatFreq	NatNCBRatio	NatContrast	NCBContrast	ShufflNatContrast	NatNCBContrastRatio
2	B	R	R	12	30.35	31.12	30.34	0.98	1.06	1.08	1.06	0.98
4	B	Y	R	12	16.21	15.13	16.21	1.07	0.90	0.86	0.90	1.05
6	B	R	Y	12	30.94	30.94	30.94	1.00	0.94	0.93	0.94	1.01
8	B	Y	Y	12	22.50	22.81	22.50	0.99	1.09	1.12	1.09	0.97
10	B	R	R	23	21.02	23.83	21.01	0.88	0.98	1.02	0.98	0.96
12	B	Y	R	23	25.22	26.86	25.22	0.94	1.02	0.99	1.02	1.03
14	B	R	Y	23	25.54	22.43	25.54	1.14	1.02	0.98	1.02	1.04
16	B	Y	Y	23	28.23	26.89	28.22	1.05	0.98	1.01	0.98	0.97
18	B	R	R	31	27.62	31.39	28.38	0.88	0.97	1.00	1.00	0.97
20	B	Y	R	31	33.57	30.58	32.90	1.10	1.02	1.00	1.00	1.02
22	B	R	Y	31	18.45	19.14	17.85	0.96	1.03	1.00	1.00	1.03
24	B	Y	Y	31	20.36	18.88	20.86	1.08	0.98	1.01	1.00	0.97

	NatFreqCorOGT	NCBFreqCorOGT	NatContrastCorOGT	NCBContrastCorOGT	ShufflNatContrastCorOGT	NatNCBContrastRatioCorOGT
2	0.34	0.31	0.33	0.27	0.33	0.37
4	-0.37	-0.27	-0.39	-0.31	-0.39	-0.48
6	-0.26	-0.26	-0.35	-0.30	-0.35	-0.41
8	0.05	-0.05	0.39	0.33	0.40	0.45
10	0.67	0.66	0.51	0.73	0.51	0.39
12	-0.13	-0.28	-0.53	-0.73	-0.53	-0.41
14	-0.76	-0.38	-0.56	-0.74	-0.56	-0.43
16	-0.03	-0.15	0.58	0.75	0.58	0.43
18	0.63	0.64	0.48	0.53	0.01	0.46
20	-0.71	-0.24	-0.52	-0.54	-0.17	-0.51
22	-0.34	-0.19	-0.52	-0.53	0.02	-0.51
24	0.00	-0.20	0.56	0.56	0.17	0.55

Nucleic composition comparison of DNA, RNA and ncDNA in Archaea

	sk	base	DNANatFreq	DNANCBFreq	tRNAFreq	rRNAFreq	ncDNAFreq
1	A	A	28.45	27.94	17.13	23.68	30.73
3	A	T	23.89	24.44	18.17	19.24	30.65
5	A	G	26.00	26.12	33.91	32.08	19.30
7	A	C	21.66	21.51	30.80	24.99	19.31

Nucleic composition comparison of DNA, RNA and ncDNA in Bacteria

	sk	base	DNANatFreq	DNANCBFreq	tRNAFreq	rRNAFreq	ncDNAFreq
2	B	A	24.05	26.42	19.66	26.09	26.57
4	B	T	22.68	23.70	21.56	20.68	26.61
6	B	G	27.50	26.66	31.21	31.10	23.41
8	B	C	25.77	23.23	27.58	22.13	23.41

Dinucleotide composition comparison of DNA, RNA and ncDNA in Archaea

	sk	base1	base2	DNANatFreq	DNANCBFreq	DNAShuffledCodonsFreq	DNAShuffledCodonsNCBFreq
1	A	A	A	8.76	8.23	8.44	8.21
3	A	T	A	5.82	6.19	6.22	6.25
5	A	G	A	8.15	8.04	8.04	8.00
7	A	C	A	5.67	5.42	5.74	5.48
9	A	A	T	7.15	7.13	7.18	7.16
11	A	T	T	6.41	6.49	6.40	6.47
13	A	G	T	4.92	5.47	5.05	5.49
15	A	C	T	5.42	5.36	5.27	5.34
17	A	A	G	7.08	6.84	7.26	6.86
19	A	T	G	6.22	6.22	5.83	6.19
21	A	G	G	7.20	7.06	7.26	7.07
23	A	C	G	5.50	6.00	5.64	5.98
25	A	A	C	5.40	5.69	5.55	5.71
27	A	T	C	5.46	5.55	5.45	5.53
29	A	G	C	5.74	5.54	5.65	5.56
31	A	C	C	5.08	4.74	5.01	4.72

	DNANatContrast	DNANCBContrast	DNA31ShuffledContrast	DNA31ShuffledNCBContrast	tRNAFreq	rRNAFreq
1	1.08	1.05	1.04	1.05	3.87	7.02
3	0.86	0.91	0.91	0.92	3.43	4.64
5	1.10	1.10	1.09	1.10	4.91	7.30
7	0.92	0.90	0.93	0.91	5.03	4.72
9	1.05	1.04	1.06	1.05	3.29	3.90
11	1.12	1.09	1.12	1.08	3.51	3.76
13	0.79	0.86	0.81	0.86	6.53	6.44
15	1.05	1.02	1.02	1.02	5.03	5.13
17	0.96	0.94	0.98	0.94	6.58	7.57
19	1.00	0.98	0.94	0.97	4.30	6.12
21	1.06	1.04	1.07	1.04	13.27	11.21
23	0.98	1.07	1.00	1.07	9.09	7.19
25	0.88	0.95	0.90	0.95	2.74	5.19
27	1.06	1.06	1.05	1.05	7.06	4.71
29	1.02	0.99	1.00	0.99	9.38	7.15
31	1.08	1.02	1.07	1.02	11.97	7.95

	ncDNAFreq	tRNAContrast	rRNAContrast	ncDNAContrast
1	10.90	1.32	1.25	1.15
3	8.60	1.10	1.02	0.91
5	5.84	0.84	0.96	0.98
7	5.38	0.95	0.80	0.91
9	9.42	1.06	0.86	1.00
11	10.84	1.06	1.02	1.15
13	4.86	1.06	1.04	0.82
15	5.56	0.90	1.07	0.94
17	5.56	1.13	1.00	0.94
19	5.37	0.70	0.99	0.91
21	4.62	1.15	1.09	1.24
23	3.74	0.87	0.90	1.00
25	4.87	0.52	0.88	0.82
27	5.84	1.26	0.98	0.99
29	3.99	0.90	0.89	1.07
31	4.61	1.26	1.27	1.24

Dinucleotide composition comparison of DNA, RNA and ncDNA in Bacteria

	sk	base1	base2	DNANatFreq	DNANCBFreq	DNAShuffledCodonsFreq	DNAShuffledCodonsNCBFreq
2	B	A	A	7.25	7.38	7.14	7.38
4	B	T	A	4.08	5.53	4.43	5.58
6	B	G	A	6.68	7.42	6.78	7.39
8	B	C	A	5.98	6.03	5.71	6.08
10	B	A	T	6.20	6.42	6.26	6.46
12	B	T	T	6.19	6.35	5.97	6.31
14	B	G	T	4.94	5.26	5.16	5.29
16	B	C	T	5.36	5.69	5.29	5.64
18	B	A	G	5.34	6.73	5.42	6.74
20	B	T	G	6.87	6.31	6.70	6.27
22	B	G	G	7.10	7.32	7.35	7.37
24	B	C	G	8.20	6.31	8.02	6.26
26	B	A	C	5.21	5.84	5.24	5.85
28	B	T	C	5.55	5.52	5.58	5.54
30	B	G	C	8.78	6.65	8.21	6.61
32	B	C	C	6.26	5.23	6.75	5.24

	DNANatContrast	DNANCBContrast	DNA31ShuffledContrast	DNA31ShuffledNCBContrast
2	1.25	1.06	1.23	1.06
4	0.75	0.88	0.81	0.89
6	1.01	1.05	1.02	1.05
8	0.97	0.98	0.92	0.99
10	1.14	1.03	1.15	1.03
12	1.20	1.13	1.16	1.12
14	0.79	0.83	0.83	0.84
16	0.92	1.03	0.91	1.03
18	0.81	0.96	0.82	0.96
20	1.10	1.00	1.07	0.99
22	0.94	1.03	0.97	1.04
24	1.16	1.02	1.13	1.01
26	0.84	0.95	0.85	0.95
28	0.95	1.00	0.95	1.01
30	1.24	1.07	1.16	1.07
32	0.94	0.97	1.02	0.97

	tRNAFreq	rRNAFreq	ncDNAFreq	tRNAContrast	rRNAContrast	ncDNAContrast
2	3.93	7.80	8.92	1.02	1.15	1.26
4	3.89	5.26	6.07	0.92	0.98	0.86
6	5.54	8.20	5.70	0.90	1.01	0.92
8	6.50	4.84	5.88	1.20	0.84	0.95
10	3.36	4.55	7.28	0.79	0.84	1.03
12	4.96	4.15	8.95	1.07	0.97	1.26
14	7.82	6.93	5.01	1.16	1.08	0.80
16	5.59	5.04	5.39	0.94	1.10	0.86
18	7.83	8.17	5.38	1.28	1.01	0.87
20	4.89	6.98	5.88	0.73	1.09	0.94
22	10.31	9.52	5.88	1.06	0.98	1.07
24	7.56	6.42	6.23	0.88	0.93	1.14
26	3.73	5.57	5.00	0.69	0.96	0.80
28	8.02	4.27	5.71	1.35	0.93	0.92
30	7.84	6.46	6.84	0.91	0.94	1.25
32	8.23	5.84	5.87	1.08	1.19	1.07

Correlation between OGT and nucleic acid composition of DNA, RNA and ncDNA in Archaea

	sk	base	DNANat	DNANCB	tRNA	rRNA	ncDNA
1	A	A	0.00	-0.10	-0.76	-0.85	-0.09
3	A	T	0.03	0.27	-0.89	-0.88	-0.08
5	A	G	0.16	0.10	0.84	0.94	0.09
7	A	C	-0.16	-0.17	0.84	0.76	0.08

Correlation between OGT and nucleic acid composition of DNA, RNA and ncDNA in Bacteria

	sk	base	DNANat	DNANCB	tRNA	rRNA	ncDNA
2	B	A	0.13	0.14	-0.44	-0.52	0.03
4	B	T	-0.03	0.14	-0.51	-0.77	0.02
6	B	G	0.02	0.02	0.53	0.72	-0.02
8	B	C	-0.12	-0.34	0.39	0.60	-0.02

Correlation between OGT and dinucleotide composition of DNA, RNA and ncDNA in Archaea

	sk	base1	base2	DNANat	DNANCB	DNAShuffledCodons	DNAShuffledCodonsNCB	DNANatContrast
1	A	A	A	-0.10	0.01	-0.08	0.02	-0.27
3	A	T	A	0.27	0.20	0.20	0.16	0.65
5	A	G	A	0.16	-0.32	0.28	-0.27	-0.23
7	A	C	A	-0.51	-0.76	-0.54	-0.81	-0.38
9	A	A	T	-0.15	-0.18	-0.12	-0.17	-0.54
11	A	T	T	0.01	0.32	0.02	0.32	0.05
13	A	G	T	0.34	0.43	0.48	0.46	-0.01
15	A	C	T	0.37	0.41	0.20	0.36	0.66
17	A	A	G	0.79	0.50	0.75	0.41	0.80
19	A	T	G	-0.23	-0.11	-0.04	-0.03	-0.58
21	A	G	G	0.25	0.18	0.19	0.13	0.07
23	A	C	G	-0.19	-0.07	-0.16	-0.02	-0.29
25	A	A	C	-0.29	-0.67	-0.30	-0.68	-0.18
27	A	T	C	-0.31	0.05	-0.33	0.07	-0.29
29	A	G	C	-0.05	0.01	-0.05	0.01	0.01
31	A	C	C	-0.06	0.00	-0.09	0.01	0.32

	DNANCBContrast	DNAShufflContrast	DNAShufflNCBContrast	tRNA	rRNA	ncDNA	tRNAContrast	rRNAContrast	ncDNAContrast
1	0.43	-0.28	0.46	-0.44	-0.59	-0.09	0.82	0.92	0.13
3	0.56	0.63	0.51	-0.63	-0.83	-0.03	0.72	0.88	0.36
5	-0.45	-0.24	-0.39	-0.69	-0.53	0.05	-0.68	-0.77	-0.15
7	-0.74	-0.38	-0.80	-0.50	-0.84	-0.16	-0.52	-0.90	-0.52
9	-0.61	-0.57	-0.60	-0.74	-0.90	-0.24	0.26	0.12	-0.48
11	0.12	0.11	0.07	-0.86	-0.76	-0.08	0.80	0.29	0.16
13	0.42	0.08	0.48	-0.68	-0.73	0.09	0.52	-0.04	-0.11
15	0.57	0.57	0.58	-0.86	-0.80	0.60	-0.81	-0.48	0.63
17	0.59	0.78	0.49	-0.60	-0.37	0.62	-0.44	-0.63	0.64
19	-0.67	-0.56	-0.67	-0.75	-0.79	-0.14	-0.41	-0.72	-0.53
21	0.08	0.02	-0.01	0.89	0.95	0.14	0.44	0.72	0.00
23	-0.20	-0.29	0.07	0.74	0.80	-0.09	-0.06	-0.26	-0.31
25	-0.45	-0.16	-0.45	-0.48	-0.74	0.08	-0.54	-0.86	-0.11
27	0.03	-0.35	0.04	-0.92	-0.85	0.03	-0.81	-0.55	-0.17
29	0.17	0.02	0.17	0.81	0.86	0.04	-0.16	-0.70	0.00
31	0.60	0.37	0.65	0.90	0.89	0.14	0.80	0.83	0.01

Correlation between OGT and dinucleotide composition of DNA, RNA and ncDNA in Bacteria

	sk	base1	base2	DNANat	DNANCB	DNAShuffledCodons	DNAShuffledCodonsNCB	DNANatContrast
2	B	A	A	0.17	0.19	0.14	0.18	0.05
4	B	T	A	0.01	0.17	0.06	0.19	-0.01
6	B	G	A	0.59	0.58	0.65	0.57	0.27
8	B	C	A	-0.43	-0.68	-0.44	-0.68	-0.45
10	B	A	T	-0.06	0.02	-0.03	0.03	-0.34
12	B	T	T	0.04	0.23	0.01	0.23	0.28
14	B	G	T	-0.18	0.24	-0.15	0.29	-0.18
16	B	C	T	-0.06	-0.32	-0.08	-0.35	0.34
18	B	A	G	0.37	0.64	0.42	0.65	0.24
20	B	T	G	-0.30	-0.17	-0.27	-0.14	-0.37
22	B	G	G	0.18	0.04	0.11	0.02	0.40
24	B	C	G	-0.12	-0.11	-0.10	-0.09	-0.20
26	B	A	C	0.00	-0.53	0.06	-0.50	0.10
28	B	T	C	-0.06	-0.16	-0.18	-0.25	0.18
30	B	G	C	-0.22	-0.25	-0.17	-0.23	-0.47
32	B	C	C	-0.01	-0.15	-0.06	-0.19	0.36

	DNANCBContrast	DNAShufflContrast	DNAShufflNCBContrast	tRNA	rRNA	ncDNA	tRNAContrast	rRNAContrast	ncDNAContrast
2	0.28	-0.13	0.24	-0.10	-0.36	0.08	0.45	0.67	0.15
4	0.05	0.08	0.13	-0.56	-0.57	-0.03	-0.39	0.27	-0.03
6	0.48	0.27	0.46	-0.22	-0.07	0.15	-0.17	-0.30	0.17
8	-0.74	-0.45	-0.74	-0.09	-0.34	-0.22	-0.02	-0.54	-0.36
10	-0.48	-0.32	-0.48	-0.45	-0.74	-0.04	-0.22	-0.54	-0.25
12	0.39	0.21	0.37	-0.45	-0.70	0.07	-0.05	-0.06	0.16
14	0.25	-0.16	0.38	-0.18	-0.50	-0.17	0.11	0.22	-0.17
16	0.25	0.39	0.18	-0.37	-0.58	0.11	-0.05	-0.08	0.14
18	0.26	0.32	0.23	-0.11	0.17	0.13	-0.05	0.17	0.13
20	-0.55	-0.34	-0.55	-0.19	-0.57	-0.27	0.02	-0.13	-0.38
22	0.09	0.32	0.00	0.57	0.77	0.12	0.04	0.30	0.49
24	0.30	-0.22	0.40	0.42	0.58	-0.10	-0.01	-0.30	-0.28
26	-0.30	0.20	-0.24	-0.31	-0.51	-0.13	-0.34	-0.63	-0.17
28	0.23	0.09	0.17	-0.17	-0.46	0.15	0.17	-0.15	0.21
30	-0.58	-0.45	-0.53	0.47	0.66	-0.16	0.04	-0.19	-0.49
32	0.83	0.36	0.85	0.40	0.74	0.11	0.12	0.52	0.47
