# Supplementary material for: Molecular mechanisms of adaptation emerging from the physics and evolution of nucleic acids and proteins
Source: Nucleic Acids Res. 2013 Dec 25;42(5):2879–92. doi: 10.1093/nar/gkt1336 (PMC3950714; doi:10.1093/nar/gkt1336)
Supplement: Supplementary Data [file supp_gkt1336_nar-02158-n-2013-File004.pdf]

# Supplementary File 2

**Position-specific nucleotide composition in selected mesophilic and thermophilic genomes for unbiased comparison (mesophile vs thermophile, A vs B)**

| | mesophile | thermophile |
|---|---|---|
| A | 11 | 7 |
| B | 15 | 10 |

**Nucleic composition comparison**

| | base | codon | ThermoNatArchaeaFreq | ThermoNCBArchaeaFreq | ThermoNatBacteriaFreq |
|---|---|---|---|---|---|
| 1 | A | 1 | 30.16 | 28.44 | 23.74 |
| 2 | T | 1 | 16.26 | 17.48 | 14.89 |
| 3 | G | 1 | 36.08 | 36.08 | 37.46 |
| 4 | C | 1 | 17.51 | 18.01 | 23.92 |
| 5 | A | 2 | 29.55 | 29.60 | 27.43 |
| 6 | T | 2 | 31.31 | 31.31 | 30.20 |
| 7 | G | 2 | 17.32 | 17.51 | 18.75 |
| 8 | C | 2 | 21.81 | 21.58 | 23.62 |
| 9 | A | 3 | 21.73 | 23.93 | 16.89 |
| 10 | T | 3 | 20.30 | 25.57 | 19.34 |
| 11 | G | 3 | 28.18 | 24.92 | 31.06 |
| 12 | C | 3 | 29.79 | 25.59 | 32.72 |

| | ThermoNCBBacteriaFreq | ThermoArchNatNCBFreqRatio | ThermoBacNatNCBFreqRatio |
|---|---|---|---|
| 1 | 24.44 | 1.06 | 0.97 |
| 2 | 16.18 | 0.93 | 0.92 |
| 3 | 37.46 | 1.00 | 1.00 |
| 4 | 21.92 | 0.97 | 1.09 |
| 5 | 27.47 | 1.00 | 1.00 |
| 6 | 30.20 | 1.00 | 1.00 |
| 7 | 18.49 | 0.99 | 1.01 |
| 8 | 23.84 | 1.01 | 0.99 |
| 9 | 24.95 | 0.91 | 0.68 |
| 10 | 24.46 | 0.79 | 0.79 |
| 11 | 26.14 | 1.13 | 1.19 |
| 12 | 24.45 | 1.16 | 1.34 |

| | ThermoNatBacArchFreqRatio | ThermoNCBBacArchFreqRatio | MesoNatArchaeaFreq |
|---|---|---|---|
| 1 | 0.79 | 0.86 | 30.23 |
| 2 | 0.92 | 0.93 | 16.87 |
| 3 | 1.04 | 1.04 | 35.34 |
| 4 | 1.37 | 1.22 | 17.56 |
| 5 | 0.93 | 0.93 | 32.04 |
| 6 | 0.96 | 0.96 | 30.47 |
| 7 | 1.08 | 1.06 | 16.12 |
| 8 | 1.08 | 1.10 | 21.36 |
| 9 | 0.78 | 1.04 | 26.66 |
| 10 | 0.95 | 0.96 | 27.27 |
| 11 | 1.10 | 1.05 | 21.17 |
| 12 | 1.10 | 0.96 | 24.90 |

| | MesoNCBArchaeaFreq | MesoNatBacteriaFreq | MesoNCBBacteriaFreq | MesoArchNatNCBFreqRatio |
|---|---|---|---|---|
| 1 | 29.80 | 25.47 | 26.01 | 1.01 |
| 2 | 17.37 | 15.99 | 16.84 | 0.97 |
| 3 | 35.34 | 35.62 | 35.62 | 1.00 |
| 4 | 17.49 | 22.92 | 21.53 | 1.00 |
| 5 | 32.06 | 29.01 | 29.03 | 1.00 |
| 6 | 30.47 | 29.99 | 29.99 | 1.00 |
| 7 | 16.32 | 18.00 | 17.52 | 0.99 |
| 8 | 21.15 | 23.00 | 23.46 | 1.01 |
| 9 | 24.46 | 18.24 | 24.61 | 1.09 |
| 10 | 25.23 | 22.42 | 24.75 | 1.08 |
| 11 | 25.09 | 27.78 | 25.87 | 0.84 |
| 12 | 25.22 | 31.55 | 24.77 | 0.99 |

| | MesoBacNatNCBFreqRatio | MesoNatBacArchFreqRatio | MesoNCBBacArchFreqRatio |
|---|---|---|---|
| 1 | 0.98 | 0.84 | 0.87 |
| 2 | 0.95 | 0.95 | 0.97 |
| 3 | 1.00 | 1.01 | 1.01 |
| 4 | 1.06 | 1.31 | 1.23 |
| 5 | 1.00 | 0.91 | 0.91 |
| 6 | 1.00 | 0.98 | 0.98 |
| 7 | 1.03 | 1.12 | 1.07 |
| 8 | 0.98 | 1.08 | 1.11 |
| 9 | 0.74 | 0.68 | 1.01 |
| 10 | 0.91 | 0.82 | 0.98 |
| 11 | 1.07 | 1.31 | 1.03 |
| 12 | 1.27 | 1.27 | 0.98 |

Nucleic combination composition comparison

| | bases | codon | ThermoNatArchFreq | ThermoNCBArchFreq | ThermoNatBacFreq | ThermoNCBBacFreq |
|---|---|---|---|---|---|---|
| 1 | A+T | 1 | 46.41 | 45.91 | 38.62 | 40.62 |
| 2 | A+G | 1 | 66.24 | 64.52 | 61.19 | 61.89 |
| 3 | A+C | 1 | 47.66 | 46.44 | 47.66 | 46.36 |
| 4 | T+G | 1 | 52.34 | 53.56 | 52.34 | 53.64 |
| 5 | T+C | 1 | 33.76 | 35.48 | 38.81 | 38.11 |
| 6 | G+C | 1 | 53.59 | 54.09 | 61.38 | 59.38 |
| 7 | A+T | 2 | 60.87 | 60.92 | 57.63 | 57.66 |
| 8 | A+G | 2 | 46.87 | 47.11 | 46.18 | 45.96 |
| 9 | A+C | 2 | 51.36 | 51.18 | 51.06 | 51.31 |
| 10 | T+G | 2 | 48.64 | 48.82 | 48.94 | 48.69 |
| 11 | T+C | 2 | 53.13 | 52.89 | 53.82 | 54.04 |
| 12 | G+C | 2 | 39.13 | 39.08 | 42.37 | 42.34 |
| 13 | A+T | 3 | 42.03 | 49.50 | 36.23 | 49.40 |
| 14 | A+G | 3 | 49.91 | 48.85 | 47.95 | 51.09 |
| 15 | A+C | 3 | 51.52 | 49.52 | 49.61 | 49.40 |
| 16 | T+G | 3 | 48.48 | 50.48 | 50.39 | 50.60 |
| 17 | T+C | 3 | 50.09 | 51.15 | 52.05 | 48.91 |
| 18 | G+C | 3 | 57.97 | 50.50 | 63.77 | 50.60 |

| | ThermoArchNatNCBFreqRatio | ThermoBacNatNCBFreqRatio | ThermoNatBacArchFreqRatio |
|---|---|---|---|
| 1 | 1.01 | 0.95 | 0.83 |
| 2 | 1.03 | 0.99 | 0.92 |
| 3 | 1.03 | 1.03 | 1.00 |
| 4 | 0.98 | 0.98 | 1.00 |
| 5 | 0.95 | 1.02 | 1.15 |
| 6 | 0.99 | 1.03 | 1.15 |
| 7 | 1.00 | 1.00 | 0.95 |
| 8 | 0.99 | 1.00 | 0.99 |
| 9 | 1.00 | 1.00 | 0.99 |
| 10 | 1.00 | 1.01 | 1.01 |
| 11 | 1.00 | 1.00 | 1.01 |
| 12 | 1.00 | 1.00 | 1.08 |
| 13 | 0.85 | 0.73 | 0.86 |
| 14 | 1.02 | 0.94 | 0.96 |
| 15 | 1.04 | 1.00 | 0.96 |
| 16 | 0.96 | 1.00 | 1.04 |
| 17 | 0.98 | 1.06 | 1.04 |
| 18 | 1.15 | 1.26 | 1.10 |

| | ThermoNCBBacArchFreqRatio | MesoNatArchFreq | MesoNCBArchFreq | MesoNatBacFreq |
|---|---|---|---|---|
| 1 | 0.88 | 47.10 | 47.17 | 41.46 |
| 2 | 0.96 | 65.57 | 65.14 | 61.09 |
| 3 | 1.00 | 47.79 | 47.29 | 48.39 |
| 4 | 1.00 | 52.21 | 52.71 | 51.61 |
| 5 | 1.07 | 34.43 | 34.86 | 38.91 |
| 6 | 1.10 | 52.90 | 52.83 | 58.54 |
| 7 | 0.95 | 62.52 | 62.53 | 59.00 |
| 8 | 0.98 | 48.17 | 48.38 | 47.01 |
| 9 | 1.00 | 53.40 | 53.20 | 52.00 |
| 10 | 1.00 | 46.60 | 46.80 | 48.00 |
| 11 | 1.02 | 51.83 | 51.62 | 52.99 |
| 12 | 1.08 | 37.48 | 37.47 | 41.00 |
| 13 | 1.00 | 53.93 | 49.69 | 40.66 |
| 14 | 1.05 | 47.83 | 49.55 | 46.02 |
| 15 | 1.00 | 51.56 | 49.68 | 49.79 |
| 16 | 1.00 | 48.44 | 50.32 | 50.21 |
| 17 | 0.96 | 52.17 | 50.45 | 53.98 |
| 18 | 1.00 | 46.07 | 50.31 | 59.34 |

| | MesoNCBBacFreq | MesoArchNatNCBFreqRatio | MesoBacNatNCBFreqRatio | MesoNatBacArchFreqRatio |
|---|---|---|---|---|
| 1 | 42.85 | 1.00 | 0.97 | 0.88 |
| 2 | 61.63 | 1.01 | 0.99 | 0.93 |
| 3 | 47.54 | 1.01 | 1.02 | 1.01 |
| 4 | 52.46 | 0.99 | 0.98 | 0.99 |
| 5 | 38.37 | 0.99 | 1.01 | 1.13 |
| 6 | 57.15 | 1.00 | 1.02 | 1.11 |
| 7 | 59.03 | 1.00 | 1.00 | 0.94 |
| 8 | 46.55 | 1.00 | 1.01 | 0.98 |
| 9 | 52.49 | 1.00 | 0.99 | 0.97 |
| 10 | 47.51 | 1.00 | 1.01 | 1.03 |
| 11 | 53.45 | 1.00 | 0.99 | 1.02 |
| 12 | 40.97 | 1.00 | 1.00 | 1.09 |
| 13 | 49.36 | 1.09 | 0.82 | 0.75 |
| 14 | 50.48 | 0.97 | 0.91 | 0.96 |
| 15 | 49.38 | 1.04 | 1.01 | 0.97 |
| 16 | 50.62 | 0.96 | 0.99 | 1.04 |
| 17 | 49.52 | 1.03 | 1.09 | 1.03 |
| 18 | 50.64 | 0.92 | 1.17 | 1.29 |

| | MesoNCBBacArchFreqRatio |
|---|---|
| 1 | 0.91 |
| 2 | 0.95 |
| 3 | 1.01 |
| 4 | 1.00 |
| 5 | 1.10 |
| 6 | 1.08 |
| 7 | 0.94 |
| 8 | 0.96 |
| 9 | 0.99 |
| 10 | 1.02 |
| 11 | 1.04 |
| 12 | 1.09 |
| 13 | 0.99 |
| 14 | 1.02 |
| 15 | 0.99 |
| 16 | 1.01 |
| 17 | 0.98 |
| 18 | 1.01 |

Codon usage (selected thermophiles and mesophiles)

| | aa | codon | ThermoArchaeaFreq | ThermoBacteriaFreq | ThermoBacArchFreqRatio | MesoArchaeaFreq |
|---|---|---|---|---|---|---|
| 1 | A | GCT | 15.43 | 13.33 | 0.86 | 21.77 |
| 2 | A | GCC | 32.21 | 39.29 | 1.22 | 26.20 |
| 3 | A | GCA | 26.98 | 15.74 | 0.58 | 35.37 |
| 4 | A | GCG | 25.38 | 31.65 | 1.25 | 16.66 |
| 5 | L | TTA | 9.32 | 9.08 | 0.97 | 16.81 |
| 6 | L | TTG | 8.14 | 14.17 | 1.74 | 8.76 |
| 7 | L | CTT | 21.52 | 14.56 | 0.68 | 27.04 |
| 8 | L | CTC | 26.98 | 23.91 | 0.89 | 22.36 |
| 9 | L | CTA | 8.92 | 3.44 | 0.39 | 4.54 |
| 10 | L | CTG | 25.13 | 34.84 | 1.39 | 20.49 |
| 11 | R | CGT | 5.34 | 10.34 | 1.94 | 10.31 |
| 12 | R | CGC | 14.66 | 34.33 | 2.34 | 13.67 |
| 13 | R | CGA | 3.41 | 5.83 | 1.71 | 7.86 |
| 14 | R | CGG | 9.90 | 29.78 | 3.01 | 20.98 |
| 15 | R | AGA | 23.60 | 10.77 | 0.46 | 26.53 |
| 16 | R | AGG | 43.09 | 8.95 | 0.21 | 20.64 |
| 17 | K | AAA | 38.06 | 56.04 | 1.47 | 70.68 |
| 18 | K | AAG | 61.94 | 43.96 | 0.71 | 29.32 |
| 19 | N | AAT | 42.76 | 47.23 | 1.10 | 57.12 |
| 20 | N | AAC | 57.24 | 52.77 | 0.92 | 42.88 |
| 21 | M | ATG | 100.00 | 100.00 | 1.00 | 100.00 |
| 22 | D | GAT | 49.96 | 45.45 | 0.91 | 55.40 |
| 23 | D | GAC | 50.04 | 54.55 | 1.09 | 44.60 |
| 24 | F | TTT | 37.17 | 48.89 | 1.32 | 56.97 |
| 25 | F | TTC | 62.83 | 51.11 | 0.81 | 43.03 |
| 26 | C | TGT | 34.08 | 36.09 | 1.06 | 52.22 |
| 27 | C | TGC | 65.92 | 63.91 | 0.97 | 47.78 |
| 28 | P | CCT | 20.29 | 14.04 | 0.69 | 28.16 |
| 29 | P | CCC | 25.69 | 32.91 | 1.28 | 20.90 |
| 30 | P | CCA | 26.46 | 13.20 | 0.50 | 21.45 |
| 31 | P | CCG | 27.57 | 39.85 | 1.45 | 29.49 |
| 32 | Q | CAA | 19.79 | 32.81 | 1.66 | 29.31 |
| 33 | Q | CAG | 80.21 | 67.19 | 0.84 | 70.69 |
| 34 | S | TCT | 12.88 | 10.55 | 0.82 | 17.49 |
| 35 | S | TCC | 19.35 | 21.58 | 1.12 | 17.88 |
| 36 | S | TCA | 23.38 | 11.62 | 0.50 | 21.99 |
| 37 | S | TCG | 14.60 | 18.59 | 1.27 | 12.67 |
| 38 | S | AGT | 8.67 | 13.44 | 1.55 | 14.46 |
| 39 | S | AGC | 21.11 | 24.22 | 1.15 | 15.50 |
| 40 | E | GAA | 36.16 | 49.75 | 1.38 | 65.26 |
| 41 | E | GAG | 63.84 | 50.25 | 0.79 | 34.74 |
| 42 | T | ACT | 14.34 | 12.82 | 0.89 | 23.35 |
| 43 | T | ACC | 31.88 | 43.79 | 1.37 | 30.75 |
| 44 | T | ACA | 28.84 | 16.83 | 0.58 | 27.52 |
| 45 | T | ACG | 24.94 | 26.55 | 1.06 | 18.38 |
| 46 | G | GGT | 21.10 | 18.99 | 0.90 | 20.22 |
| 47 | G | GGC | 33.10 | 38.47 | 1.16 | 23.57 |
| 48 | G | GGA | 25.74 | 19.16 | 0.74 | 35.41 |
| 49 | G | GGG | 20.05 | 23.39 | 1.17 | 20.81 |
| 50 | W | TGG | 100.00 | 100.00 | 1.00 | 100.00 |
| 51 | H | CAT | 42.32 | 37.42 | 0.88 | 52.08 |
| 52 | H | CAC | 57.68 | 62.58 | 1.08 | 47.92 |
| 53 | Y | TAT | 40.31 | 44.23 | 1.10 | 55.94 |
| 54 | Y | TAC | 59.69 | 55.77 | 0.93 | 44.06 |
| 55 | I | ATT | 18.53 | 35.81 | 1.93 | 38.41 |
| 56 | I | ATC | 29.41 | 42.40 | 1.44 | 34.54 |
| 57 | I | ATA | 52.06 | 21.79 | 0.42 | 27.04 |
| 58 | V | GTT | 25.87 | 19.95 | 0.77 | 31.16 |
| 59 | V | GTC | 27.69 | 29.47 | 1.06 | 28.38 |
| 60 | V | GTA | 16.33 | 11.79 | 0.72 | 23.04 |
| 61 | V | GTG | 30.11 | 38.79 | 1.29 | 17.42 |
| 62 | * | TAG | 20.59 | 24.63 | 1.20 | 10.55 |
| 63 | * | TGA | 48.07 | 44.03 | 0.92 | 37.16 |
| 64 | * | TAA | 31.34 | 31.34 | 1.00 | 52.29 |

| | MesoBacteriaFreq | MesoBacArchFreqRatio |
|---|---|---|
| 1 | 17.29 | 0.79 |
| 2 | 41.33 | 1.58 |
| 3 | 13.93 | 0.39 |
| 4 | 27.45 | 1.65 |
| 5 | 16.99 | 1.01 |
| 6 | 12.78 | 1.46 |
| 7 | 10.88 | 0.40 |
| 8 | 14.14 | 0.63 |
| 9 | 5.45 | 1.20 |
| 10 | 39.75 | 1.94 |
| 11 | 14.43 | 1.40 |
| 12 | 43.60 | 3.19 |
| 13 | 5.71 | 0.73 |
| 14 | 20.14 | 0.96 |
| 15 | 10.63 | 0.40 |
| 16 | 5.49 | 0.27 |
| 17 | 56.52 | 0.80 |
| 18 | 43.48 | 1.48 |
| 19 | 55.63 | 0.97 |
| 20 | 44.37 | 1.03 |
| 21 | 100.00 | 1.00 |
| 22 | 49.35 | 0.89 |
| 23 | 50.65 | 1.14 |
| 24 | 50.60 | 0.89 |
| 25 | 49.40 | 1.15 |
| 26 | 39.04 | 0.75 |
| 27 | 60.96 | 1.28 |
| 28 | 17.78 | 0.63 |
| 29 | 27.52 | 1.32 |
| 30 | 15.26 | 0.71 |
| 31 | 39.44 | 1.34 |
| 32 | 43.68 | 1.49 |
| 33 | 56.32 | 0.80 |
| 34 | 13.86 | 0.79 |
| 35 | 15.58 | 0.87 |
| 36 | 12.10 | 0.55 |
| 37 | 17.25 | 1.36 |
| 38 | 15.45 | 1.07 |
| 39 | 25.76 | 1.66 |
| 40 | 55.34 | 0.85 |
| 41 | 44.66 | 1.29 |
| 42 | 20.90 | 0.90 |
| 43 | 42.99 | 1.40 |
| 44 | 17.44 | 0.63 |
| 45 | 18.67 | 1.02 |
| 46 | 22.31 | 1.10 |
| 47 | 47.23 | 2.00 |
| 48 | 15.08 | 0.43 |
| 49 | 15.38 | 0.74 |
| 50 | 100.00 | 1.00 |
| 51 | 47.85 | 0.92 |
| 52 | 52.15 | 1.09 |
| 53 | 54.41 | 0.97 |
| 54 | 45.59 | 1.03 |
| 55 | 38.44 | 1.00 |
| 56 | 44.86 | 1.30 |
| 57 | 16.70 | 0.62 |
| 58 | 19.82 | 0.64 |
| 59 | 27.62 | 0.97 |
| 60 | 15.08 | 0.65 |
| 61 | 37.48 | 2.15 |
| 62 | 20.38 | 1.93 |
| 63 | 42.35 | 1.14 |
| 64 | 37.27 | 0.71 |
