# Supplementary material for: Molecular mechanisms of adaptation emerging from the physics and evolution of nucleic acids and proteins
Source: Nucleic Acids Res. 2013 Dec 25;42(5):2879–92. doi: 10.1093/nar/gkt1336 (PMC3950714; doi:10.1093/nar/gkt1336)
Supplement: Supplementary Data [file supp_gkt1336_nar-02158-n-2013-File005.pdf]

Supplementary File 3

Position-specific nucleotide composition (aerobes vs anaerobes, A vs B)

		Aerobic	Anaerobic	Facultative	Microaerophilic
A	2	8	29	5	1
B	11	71	38	71	6

Nucleic composition comparison

	base	codon	NatAnaerobicFreq	NCBAnaerobicFreq	NatAerobicFreq	NCBAerobicFreq	NatArchaeaFreq
1	A	1	29.57	29.00	22.67	23.82	29.97
2	T	1	16.96	17.31	14.55	16.10	16.60
3	G	1	34.94	34.94	38.06	38.06	36.06
4	C	1	18.53	18.74	24.72	22.03	17.37
5	A	2	31.19	31.20	26.85	26.91	30.72
6	T	2	30.98	30.98	28.99	28.99	31.15
7	G	2	16.83	16.73	19.16	18.87	16.86
8	C	2	21.00	21.09	25.00	25.23	21.26
9	A	3	24.64	24.71	13.51	24.47	24.97
10	T	3	26.42	24.83	16.35	24.70	24.27
11	G	3	23.81	25.61	32.46	26.14	24.74
12	C	3	25.13	24.85	37.67	24.70	26.02

	NCBArchaeaFreq	NatBacteriaFreq	NCBBacteriaFreq	NatAeroAnaeroFreqRatio	NCBAeroAnaeroFreqRatio
1	28.60	25.06	25.90	0.77	0.82
2	17.21	15.97	16.65	0.86	0.93
3	36.06	36.25	36.25	1.09	1.09
4	18.13	22.71	21.20	1.33	1.18
5	30.74	28.81	28.83	0.86	0.86
6	31.15	29.73	29.73	0.94	0.94
7	16.83	17.95	17.68	1.14	1.13
8	21.27	23.52	23.76	1.19	1.20
9	24.64	18.29	24.53	0.55	0.99
10	25.02	22.32	24.71	0.62	0.99
11	25.33	28.29	26.04	1.36	1.02
12	25.02	31.09	24.72	1.50	0.99

	NatBacArchFreqRatio	NCBBacArchFreqRatio
1	0.84	0.91
2	0.96	0.97
3	1.01	1.01
4	1.31	1.17
5	0.94	0.94
6	0.95	0.95
7	1.06	1.05
8	1.11	1.12
9	0.73	1.00
10	0.92	0.99
11	1.14	1.03
12	1.19	0.99

Nucleic combination composition comparison

	bases	codon	NatAnaerobicFreq	NCBAnaerobicFreq	NatAerobicFreq	NCBAerobicFreq	NatArchaeaFreq
1	A+T	1	46.53	46.32	37.22	39.92	46.57
2	A+G	1	64.51	63.94	60.72	61.88	66.03
3	A+C	1	48.10	47.75	47.39	45.85	47.34
4	T+G	1	51.90	52.25	52.61	54.15	52.66
5	T+C	1	35.49	36.06	39.28	38.12	33.97
6	G+C	1	53.47	53.68	62.78	60.08	53.43
7	A+T	2	62.17	62.18	55.84	55.90	61.88
8	A+G	2	48.02	47.93	46.02	45.78	47.59
9	A+C	2	52.19	52.28	51.85	52.15	51.98
10	T+G	2	47.81	47.72	48.15	47.85	48.02
11	T+C	2	51.98	52.07	53.98	54.22	52.41
12	G+C	2	37.83	37.82	44.16	44.10	38.12
13	A+T	3	51.06	49.54	29.87	49.16	49.24
14	A+G	3	48.45	50.32	45.97	50.61	49.71
15	A+C	3	49.77	49.56	51.19	49.16	50.99
16	T+G	3	50.23	50.44	48.81	50.84	49.01
17	T+C	3	51.55	49.68	54.03	49.39	50.29
18	G+C	3	48.94	50.46	70.13	50.84	50.76

	NCBArchaeaFreq	NatBacteriaFreq	NCBBacteriaFreq	NatAeroAnaeroFreqRatio	NCBAeroAnaeroFreqRatio
1	45.81	41.03	42.55	0.80	0.86
2	64.66	61.31	62.15	0.94	0.97
3	46.73	47.77	47.09	0.99	0.96
4	53.27	52.23	52.91	1.01	1.04
5	35.34	38.69	37.85	1.11	1.06
6	54.19	58.97	57.45	1.17	1.12
7	61.90	58.53	58.56	0.90	0.90
8	47.58	46.76	46.51	0.96	0.96
9	52.01	52.32	52.60	0.99	1.00
10	47.99	47.68	47.40	1.01	1.00
11	52.42	53.24	53.49	1.04	1.04
12	38.10	41.47	41.44	1.17	1.17
13	49.66	40.62	49.24	0.58	0.99
14	49.96	46.59	50.57	0.95	1.01
15	49.65	49.38	49.25	1.03	0.99
16	50.35	50.62	50.75	0.97	1.01
17	50.04	53.41	49.43	1.05	0.99
18	50.34	59.38	50.76	1.43	1.01

	NatBacArchFreqRatio	NCBBacArchFreqRatio
1	0.88	0.93
2	0.93	0.96
3	1.01	1.01
4	0.99	0.99
5	1.14	1.07
6	1.10	1.06
7	0.95	0.95
8	0.98	0.98
9	1.01	1.01
10	0.99	0.99
11	1.02	1.02
12	1.09	1.09
13	0.82	0.99
14	0.94	1.01
15	0.97	0.99
16	1.03	1.01
17	1.06	0.99
18	1.17	1.01

Codon usage (complete dataset)

	aa	codon	AnaerobicFreq	AerobicFreq	ArchaeaFreq	BacteriaFreq	AeroAnaeroFreqRatio	BacArchFreqRatio
1	A	GCT	22.64	10.68	21.69	15.72	0.47	0.72
2	A	GCC	32.78	42.90	28.58	37.26	1.31	1.30
3	A	GCA	26.22	12.60	28.72	16.87	0.48	0.59
4	A	GCG	18.36	33.82	21.01	30.15	1.84	1.44
5	L	TTA	17.27	8.80	17.24	15.05	0.51	0.87
6	L	TTG	13.41	11.94	9.83	14.14	0.89	1.44
7	L	CTT	20.73	9.54	21.08	12.68	0.46	0.60
8	L	CTC	18.35	20.70	22.63	16.85	1.13	0.74
9	L	CTA	6.29	4.17	9.69	5.39	0.66	0.56
10	L	CTG	23.94	44.85	19.52	35.89	1.87	1.84
11	R	CGT	14.55	13.56	7.85	18.91	0.93	2.41
12	R	CGC	20.13	46.87	13.27	40.24	2.33	3.03
13	R	CGA	6.32	5.70	5.97	7.19	0.90	1.20
14	R	CGG	16.63	21.67	14.27	18.84	1.30	1.32
15	R	AGA	23.43	5.75	26.61	8.86	0.25	0.33
16	R	AGG	18.94	6.45	32.03	5.96	0.34	0.19
17	K	AAA	60.45	43.28	54.25	56.99	0.72	1.05
18	K	AAG	39.55	56.72	45.75	43.01	1.43	0.94
19	N	AAT	56.47	40.46	51.47	50.74	0.72	0.99
20	N	AAC	43.53	59.54	48.53	49.26	1.37	1.02
21	M	ATG	100.00	100.00	100.00	100.00	1.00	1.00
22	D	GAT	58.51	38.52	51.66	51.32	0.66	0.99
23	D	GAC	41.49	61.48	48.34	48.68	1.48	1.01
24	F	TTT	56.77	36.06	48.38	49.53	0.64	1.02
25	F	TTC	43.23	63.94	51.62	50.47	1.48	0.98
26	C	TGT	47.74	28.73	48.76	39.41	0.60	0.81
27	C	TGC	52.26	71.27	51.24	60.59	1.36	1.18
28	P	CCT	24.67	12.23	23.11	17.48	0.50	0.76
29	P	CCC	25.26	28.88	23.88	24.83	1.14	1.04
30	P	CCA	20.79	11.85	25.96	17.03	0.57	0.66
31	P	CCG	29.28	47.04	27.05	40.66	1.61	1.50
32	Q	CAA	39.18	31.67	33.60	42.59	0.81	1.27
33	Q	CAG	60.82	68.33	66.40	57.41	1.12	0.86
34	S	TCT	16.98	9.37	15.79	13.56	0.55	0.86
35	S	TCC	17.65	19.67	16.71	16.89	1.11	1.01
36	S	TCA	17.58	8.74	20.58	12.91	0.50	0.63
37	S	TCG	12.94	24.75	13.36	19.15	1.91	1.43
38	S	AGT	15.74	9.90	14.39	13.70	0.63	0.95
39	S	AGC	19.11	27.57	19.16	23.79	1.44	1.24
40	E	GAA	59.33	47.21	49.53	56.36	0.80	1.14
41	E	GAG	40.67	52.79	50.47	43.64	1.30	0.86
42	T	ACT	21.65	11.49	22.64	16.41	0.53	0.72
43	T	ACC	33.14	47.42	28.05	40.28	1.43	1.44
44	T	ACA	25.92	12.55	27.68	17.90	0.48	0.65
45	T	ACG	19.29	28.54	21.64	25.41	1.48	1.17
46	G	GGT	24.11	17.52	21.63	22.99	0.73	1.06
47	G	GGC	27.88	54.67	28.00	45.46	1.96	1.62
48	G	GGA	29.63	11.51	29.45	15.70	0.39	0.53
49	G	GGG	18.38	16.30	20.92	15.85	0.89	0.76
50	W	TGG	100.00	100.00	100.00	100.00	1.00	1.00
51	H	CAT	53.52	41.34	45.29	50.10	0.77	1.11
52	H	CAC	46.48	58.66	54.71	49.90	1.26	0.91
53	Y	TAT	56.46	44.07	48.17	53.89	0.78	1.12
54	Y	TAC	43.54	55.93	51.83	46.11	1.28	0.89
55	I	ATT	35.78	28.44	32.22	38.95	0.79	1.21
56	I	ATC	32.47	58.11	29.66	46.54	1.79	1.57
57	I	ATA	31.76	13.45	38.12	14.50	0.42	0.38
58	V	GTT	29.39	14.06	29.46	21.00	0.48	0.71
59	V	GTC	22.43	34.77	25.65	28.58	1.55	1.11
60	V	GTA	21.78	10.51	21.16	14.35	0.48	0.68
61	V	GTG	26.40	40.66	23.73	36.07	1.54	1.52
62	*	TAG	18.72	19.54	18.95	18.74	1.04	0.99
63	*	TGA	35.10	52.08	38.21	41.00	1.48	1.07
64	*	TAA	46.18	28.37	42.84	40.26	0.61	0.94

>
