# Supplementary material for: Molecular mechanisms of adaptation emerging from the physics and evolution of nucleic acids and proteins
Source: Nucleic Acids Res. 2013 Dec 25;42(5):2879–92. doi: 10.1093/nar/gkt1336 (PMC3950714; doi:10.1093/nar/gkt1336)
Supplement: Supplementary Data [file supp_gkt1336_nar-02158-n-2013-File006.pdf]

# Supplementary File 4

## Position-specific nucleotide composition in mesophilic genomes (aerobes vs anaerobes, A vs B)

| | | Aerobic | Anaerobic | Facultative | Microaerophilic |
|---|---|---|---|---|---|
| mesophile | 9 | 62 | 24 | 60 | 5 |

### Nucleic composition comparison

| | base | codon | NatAnaerobicFreq | NCBAnaerobicFreq | NatAerobicFreq | NCBAerobicFreq | NatBacteriaFreq |
|---|---|---|---|---|---|---|---|
| 1 | A | 1 | 28.46 | 28.76 | 22.29 | 23.65 | 24.88 |
| 2 | T | 1 | 17.34 | 17.49 | 14.44 | 16.03 | 15.92 |
| 3 | G | 1 | 34.53 | 34.53 | 38.07 | 38.07 | 36.28 |
| 4 | C | 1 | 19.67 | 19.22 | 25.21 | 22.25 | 22.92 |
| 5 | A | 2 | 31.09 | 31.09 | 26.64 | 26.70 | 28.74 |
| 6 | T | 2 | 30.32 | 30.32 | 28.79 | 28.79 | 29.58 |
| 7 | G | 2 | 16.98 | 16.90 | 19.29 | 18.99 | 17.99 |
| 8 | C | 2 | 21.61 | 21.69 | 25.28 | 25.52 | 23.69 |
| 9 | A | 3 | 22.75 | 24.42 | 12.87 | 24.42 | 17.89 |
| 10 | T | 3 | 26.74 | 24.95 | 16.15 | 24.71 | 21.91 |
| 11 | G | 3 | 24.00 | 25.66 | 32.72 | 26.15 | 28.55 |
| 12 | C | 3 | 26.52 | 24.98 | 38.26 | 24.72 | 31.65 |

| | NCBBacteriaFreq | NatAeroAnaeroFreqRatio | NCBAeroAnaeroFreqRatio |
|---|---|---|---|
| 1 | 25.84 | 0.78 | 0.82 |
| 2 | 16.63 | 0.83 | 0.92 |
| 3 | 36.28 | 1.10 | 1.10 |
| 4 | 21.24 | 1.28 | 1.16 |
| 5 | 28.77 | 0.86 | 0.86 |
| 6 | 29.58 | 0.95 | 0.95 |
| 7 | 17.74 | 1.14 | 1.12 |
| 8 | 23.91 | 1.17 | 1.18 |
| 9 | 24.48 | 0.57 | 1.00 |
| 10 | 24.74 | 0.60 | 0.99 |
| 11 | 26.03 | 1.36 | 1.02 |
| 12 | 24.74 | 1.44 | 0.99 |

### Nucleic combination composition comparison

| | bases | codon | NatAnaerobicFreq | NCBAnaerobicFreq | NatAerobicFreq | NCBAerobicFreq | NatBacteriaFreq |
|---|---|---|---|---|---|---|---|
| 1 | A+T | 1 | 45.80 | 46.24 | 36.73 | 39.68 | 40.79 |
| 2 | A+G | 1 | 62.99 | 63.29 | 60.36 | 61.72 | 61.16 |
| 3 | A+C | 1 | 48.13 | 47.98 | 47.50 | 45.91 | 47.80 |
| 4 | T+G | 1 | 51.87 | 52.02 | 52.50 | 54.09 | 52.20 |
| 5 | T+C | 1 | 37.01 | 36.71 | 39.64 | 38.28 | 38.84 |
| 6 | G+C | 1 | 54.20 | 53.76 | 63.27 | 60.32 | 59.21 |
| 7 | A+T | 2 | 61.41 | 61.41 | 55.43 | 55.49 | 58.32 |
| 8 | A+G | 2 | 48.07 | 47.99 | 45.93 | 45.69 | 46.73 |
| 9 | A+C | 2 | 52.70 | 52.78 | 51.92 | 52.23 | 52.43 |
| 10 | T+G | 2 | 47.30 | 47.22 | 48.08 | 47.77 | 47.57 |
| 11 | T+C | 2 | 51.93 | 52.01 | 54.07 | 54.31 | 53.27 |
| 12 | G+C | 2 | 38.59 | 38.59 | 44.57 | 44.51 | 41.68 |
| 13 | A+T | 3 | 49.48 | 49.36 | 29.03 | 49.13 | 39.81 |
| 14 | A+G | 3 | 46.74 | 50.07 | 45.59 | 50.57 | 46.44 |
| 15 | A+C | 3 | 49.27 | 49.39 | 51.13 | 49.13 | 49.54 |
| 16 | T+G | 3 | 50.73 | 50.61 | 48.87 | 50.87 | 50.46 |
| 17 | T+C | 3 | 53.26 | 49.93 | 54.41 | 49.43 | 53.56 |
| 18 | G+C | 3 | 50.52 | 50.64 | 70.97 | 50.87 | 60.19 |

| | NCBBacteriaFreq | NatAeroAnaeroFreqRatio | NCBAeroAnaeroFreqRatio |
|---|---|---|---|
| 1 | 42.47 | 0.80 | 0.86 |
| 2 | 62.13 | 0.96 | 0.98 |
| 3 | 47.09 | 0.99 | 0.96 |
| 4 | 52.91 | 1.01 | 1.04 |
| 5 | 37.87 | 1.07 | 1.04 |
| 6 | 57.53 | 1.17 | 1.12 |
| 7 | 58.35 | 0.90 | 0.90 |
| 8 | 46.51 | 0.96 | 0.95 |
| 9 | 52.68 | 0.99 | 0.99 |
| 10 | 47.32 | 1.02 | 1.01 |
| 11 | 53.49 | 1.04 | 1.04 |
| 12 | 41.65 | 1.15 | 1.15 |
| 13 | 49.23 | 0.59 | 1.00 |
| 14 | 50.51 | 0.98 | 1.01 |
| 15 | 49.23 | 1.04 | 0.99 |
| 16 | 50.77 | 0.96 | 1.01 |
| 17 | 49.49 | 1.02 | 0.99 |
| 18 | 50.77 | 1.40 | 1.00 |
