# Supplementary material for: Molecular mechanisms of adaptation emerging from the physics and evolution of nucleic acids and proteins
Source: Nucleic Acids Res. 2013 Dec 25;42(5):2879–92. doi: 10.1093/nar/gkt1336 (PMC3950714; doi:10.1093/nar/gkt1336)
Supplement: Supplementary Data [file supp_gkt1336_nar-02158-n-2013-File007.pdf]

Supplementary File 5

Standard deviations for position-specific compositions and p-values for correlations

Archaea: Nucleic composition

	sk	base	codon	NatFreq	NCBFreq	NatNCBRatio	NatCorOGT	NCBCorOGT	NatNCBRatioCorOGT	aa
1	A	A	1	5.83	5.00	1.17	0.5540426393	3.227342e-01	1.076180e-07	RKNMSTI
3	A	T	1	2.94	1.34	2.19	0.5982491534	1.675607e-01	9.282496e-01	LFCSWY*
5	A	G	1	4.52	4.52	1.00	0.8581888734	8.581963e-01	1.388364e-01	ADEGV
7	A	C	1	4.38	1.91	2.29	0.3368514009	3.679106e-02	1.365802e-02	LRPQH
9	A	A	2	3.52	3.49	1.01	0.2360530033	2.316670e-01	6.951974e-01	KNDQEHY*
11	A	T	2	2.07	2.07	1.00	0.0000701883	7.018946e-05	5.496480e-01	LMFIV
13	A	G	2	2.29	2.15	1.07	0.1128901782	3.468385e-01	1.128663e-05	RCSGW*
15	A	C	2	2.96	2.93	1.01	0.0147566667	5.966160e-02	9.820862e-06	APST
17	A	A	3	10.90	0.79	13.80	0.9082673385	7.500232e-08	8.536611e-01	ALRKPQSETGIV*
19	A	T	3	9.59	0.80	11.99	0.5320520778	4.696406e-07	7.528143e-01	ALRNDFCPSTGHYIV
21	A	G	3	8.84	0.96	9.21	0.1505421082	7.654519e-05	2.746460e-01	ALRKMPQSETGWV*
23	A	C	3	11.81	0.80	14.76	0.5063547363	2.440266e-07	7.434094e-01	ALRNDFCPSTGHYIV

Archaea: Nucleic combination composition

	sk	bases	codon	NatFreq	NatNCBRatio	NCBFreq	NatCorOGT	NCBCorOGT	NatNCBRatioCorOGT	aa
1	A	A+T	1	8.51	1.42	6.00	5.567479e-01	6.062032e-01	1.655362e-07	LRKNMFCSTWYI*
3	A	A+G	1	2.02	1.16	1.74	1.882245e-01	4.311487e-04	3.636442e-08	ARKNMDSETGIV
5	A	A+C	1	2.39	0.69	3.44	7.568547e-01	7.655706e-01	2.806892e-08	LRKNMPQSTHI
7	A	T+G	1	2.39	0.69	3.44	7.568547e-01	7.655706e-01	8.797131e-01	ALDFCSEGWYV*
9	A	T+C	1	2.02	1.16	1.74	1.882245e-01	4.311487e-04	8.138919e-01	LRFCPQSWHY*
11	A	G+C	1	8.51	1.42	6.00	5.567479e-01	6.062032e-01	9.748712e-01	ALRDPQEGHV
13	A	A+T	2	4.78	1.01	4.75	4.759449e-01	4.736823e-01	7.591447e-01	LKNMDFQEHYIV*
15	A	A+G	2	1.55	0.98	1.58	7.220388e-01	1.751330e-01	8.629882e-01	RKNDCQSEGWHY*
17	A	A+C	2	2.33	1.09	2.14	1.345195e-08	2.398609e-07	9.409442e-01	AKNDPQSETHY*
19	A	T+G	2	2.33	1.09	2.14	1.345195e-08	2.398609e-07	3.358919e-01	LRMFCSGWIV*
21	A	T+C	2	1.55	0.98	1.58	7.220388e-01	1.751330e-01	3.173579e-01	ALMFPSTIV
23	A	G+C	2	4.78	1.01	4.75	4.759449e-01	4.736823e-01	1.018348e-05	ARCPSTGW*
25	A	A+T	3	20.11	57.46	0.35	8.142277e-01	7.690548e-01	8.413642e-01	ALRKNDFCPQSETGHYIV*
27	A	A+G	3	3.77	2.36	1.60	6.076379e-05	3.142142e-07	5.783769e-01	ALRKMPQSETGWIV*
29	A	A+C	3	3.10	8.38	0.37	2.917491e-02	9.073925e-01	5.642311e-01	ALRKNDFCPQSETGHYIV*
31	A	T+G	3	3.10	8.38	0.37	2.917491e-02	9.073925e-01	5.367956e-01	ALRKNMDFCPQSETGWHYIV*
33	A	T+C	3	3.77	2.36	1.60	6.076379e-05	3.142142e-07	4.806092e-01	ALRNDFCPSTGHYIV
35	A	G+C	3	20.11	57.46	0.35	8.142277e-01	7.690548e-01	1.697057e-01	ALRKNMDFCPQSETGWHYIV*

Archaea: Dinucleotide composition

	sk	base1	base2	positions	NatFreq	NCBFreq	ShufflNatFreq	NatNCBRatio
1	A	A	A	12	3.51	3.51	3.51	1.00
3	A	T	A	12	0.78	0.75	0.78	1.04
5	A	G	A	12	1.53	1.53	1.53	1.00
7	A	C	A	12	0.65	0.65	0.65	1.00
9	A	A	T	12	2.25	2.25	2.26	1.00
11	A	T	T	12	2.30	0.61	2.31	3.77
13	A	G	T	12	1.24	1.24	1.23	1.00
15	A	C	T	12	2.09	0.59	2.08	3.54
17	A	A	G	12	1.87	0.47	1.88	3.98
19	A	T	G	12	0.30	0.29	0.30	1.03
21	A	G	G	12	0.82	0.82	0.82	1.00
23	A	C	G	12	1.93	0.94	1.94	2.05
25	A	A	C	12	0.88	0.88	0.88	1.00
27	A	T	C	12	0.83	0.59	0.83	1.41
29	A	G	C	12	2.11	2.11	2.10	1.00
31	A	C	C	12	0.60	0.60	0.61	1.00
33	A	A	A	23	4.52	1.20	4.52	3.77
35	A	T	A	23	4.24	0.60	4.25	7.07
37	A	G	A	23	1.43	0.64	1.43	2.23
39	A	C	A	23	2.45	0.73	2.45	3.36
41	A	A	T	23	3.92	0.98	3.91	4.00
43	A	T	T	23	3.63	0.69	3.63	5.26
45	A	G	T	23	1.29	0.42	1.28	3.07
47	A	C	T	23	1.82	0.74	1.82	2.46
49	A	A	G	23	2.82	1.21	2.82	2.33
51	A	T	G	23	1.95	0.46	1.95	4.24
53	A	G	G	23	2.00	0.81	2.00	2.47
55	A	C	G	23	3.42	0.73	3.42	4.68
57	A	A	C	23	2.71	0.99	2.72	2.74
59	A	T	C	23	4.22	0.69	4.22	6.12
61	A	G	C	23	2.60	0.41	2.61	6.34
63	A	C	C	23	2.92	0.73	2.92	4.00
65	A	A	A	31	5.44	1.29	4.67	4.22
67	A	T	A	31	3.85	1.35	4.00	2.85
69	A	G	A	31	2.28	1.12	1.87	2.04
71	A	C	A	31	2.28	1.36	2.17	1.68
73	A	A	T	31	2.59	0.29	2.57	8.93
75	A	T	T	31	2.49	0.44	2.21	5.66
77	A	G	T	31	1.21	0.25	1.04	4.84
79	A	C	T	31	1.33	0.44	1.27	3.02
81	A	A	G	31	3.09	1.06	3.03	2.92
83	A	T	G	31	3.59	1.29	2.76	2.78
85	A	G	G	31	3.65	1.22	4.17	2.99
87	A	C	G	31	7.00	1.28	5.64	5.47
89	A	A	C	31	1.04	0.53	0.99	1.96
91	A	T	C	31	1.27	0.41	1.14	3.10
93	A	G	C	31	2.61	0.65	2.42	4.02
95	A	C	C	31	2.70	0.41	3.18	6.59

	NatContrast	NCBContrast	ShufflNatContrast	NatNCBContrastRatio	NatFreqCorOGT
1	1.06	1.11	1.06	0.95	9.435181e-01
3	0.78	0.75	0.78	1.04	3.884014e-03
5	1.18	1.18	1.18	1.00	3.405378e-02
7	0.74	0.71	0.74	1.04	3.268931e-07
9	1.05	1.10	1.05	0.95	5.969143e-01
11	1.25	1.32	1.25	0.95	2.008041e-01
13	0.70	0.70	0.70	1.00	6.769810e-04
15	1.29	1.13	1.29	1.14	3.710160e-01
17	1.02	0.79	1.02	1.29	4.253023e-08
19	0.76	0.73	0.76	1.04	5.286079e-01
21	1.21	1.21	1.21	1.00	3.978519e-01
23	0.76	1.17	0.76	0.65	2.290942e-02
25	0.82	0.86	0.82	0.95	5.319835e-07
27	1.15	1.10	1.15	1.05	6.858259e-06
29	1.01	1.01	1.01	1.00	6.694791e-01
31	1.15	1.10	1.15	1.05	6.458895e-02
33	1.01	1.04	1.01	0.97	1.236052e-01
35	0.92	1.00	0.92	0.92	1.044397e-02
37	0.95	0.90	0.95	1.06	2.825781e-01
39	1.14	1.02	1.14	1.12	1.092439e-01
41	1.00	0.97	1.00	1.03	1.334255e-01
43	1.15	1.03	1.15	1.12	8.060381e-01
45	0.82	1.00	0.81	0.82	4.091216e-01
47	0.93	1.00	0.93	0.93	3.287665e-01
49	1.05	1.02	1.05	1.03	3.157355e-04
51	0.90	0.95	0.91	0.95	4.376431e-01
53	1.20	1.09	1.21	1.10	9.843193e-03
55	0.90	0.99	0.90	0.91	3.234643e-01
57	0.93	0.97	0.93	0.96	7.388727e-01
59	1.03	1.03	1.03	1.00	3.979810e-01
61	1.02	1.00	1.02	1.02	4.725588e-01
63	1.03	1.00	1.03	1.03	6.037106e-01
65	1.19	1.01	1.07	1.18	7.824776e-01
67	0.90	0.98	1.06	0.92	5.181163e-01
69	0.99	1.01	0.95	0.98	5.347918e-02
71	0.90	0.98	0.93	0.92	4.713330e-01
73	1.04	0.98	1.06	1.06	5.405643e-01
75	1.06	1.02	1.05	1.04	4.924951e-01
77	0.86	0.98	0.96	0.88	1.363082e-01
79	1.04	1.02	0.94	1.02	2.030422e-02
81	0.90	0.99	0.96	0.91	7.596671e-02
83	1.10	1.01	0.96	1.09	1.405156e-01
85	1.01	1.00	1.03	1.01	9.338882e-02
87	1.00	1.01	1.05	0.99	3.043412e-01
89	0.80	0.98	0.91	0.82	3.560901e-01
91	0.93	1.01	0.93	0.92	2.220652e-01
93	1.12	0.99	1.06	1.13	8.885833e-01
95	1.14	1.02	1.09	1.12	4.429799e-01

	NCBFreqCorOGT	NatContrastCorOGT	NCBContrastCorOGT	ShufflNatContrastCorOGT
1	9.435169e-01	3.549925e-01	2.920780e-03	3.446953e-01
3	2.596314e-03	4.472441e-04	8.694792e-08	5.019518e-04
5	3.405103e-02	6.958123e-02	6.531160e-02	7.847738e-02
7	3.268754e-07	2.875012e-03	1.802704e-08	2.699242e-03
9	5.969117e-01	2.498612e-09	2.491658e-03	2.731210e-09
11	5.002444e-03	4.054737e-01	9.379440e-02	3.709749e-01
13	6.769947e-04	1.139045e-03	1.139124e-03	1.072616e-03
15	1.812306e-11	1.170560e-01	8.072495e-01	1.256086e-01
17	4.400681e-01	5.918266e-10	4.483568e-01	6.771308e-10
19	5.225477e-01	1.805134e-02	2.235552e-02	1.974617e-02
21	3.978969e-01	4.353506e-02	1.461112e-01	3.804244e-02
23	5.012829e-03	8.280257e-04	2.666955e-08	8.397560e-04
25	5.319511e-07	5.610511e-03	2.035826e-02	5.892856e-03
27	1.604712e-03	1.279199e-05	1.685182e-02	1.212315e-05
29	6.694754e-01	7.555624e-05	2.260220e-03	1.009264e-04
31	6.459085e-02	1.207206e-02	1.181293e-02	1.131565e-02
33	2.815727e-01	2.524064e-04	2.998028e-03	2.511132e-04
35	1.023373e-06	2.204252e-06	5.686611e-02	1.979335e-06
37	8.229282e-02	8.801485e-01	5.881032e-01	9.304391e-01
39	6.257420e-02	1.275804e-02	1.097056e-07	1.430483e-02
41	2.782281e-04	6.311939e-04	3.002172e-03	6.183269e-04
43	8.865667e-03	9.732209e-01	2.795982e-04	9.937641e-01
45	1.637880e-01	1.970337e-01	5.995087e-03	2.027859e-01
47	5.793092e-02	6.225661e-06	1.525095e-07	5.884920e-06
49	2.847105e-01	1.220005e-07	1.049901e-02	1.253988e-07
51	7.387630e-04	2.242769e-04	7.841352e-04	2.139581e-04
53	8.625973e-03	2.470212e-01	3.368820e-05	2.481100e-01
55	6.087119e-02	6.708664e-02	2.463275e-04	6.690843e-02
57	1.825559e-04	1.998467e-01	1.482798e-03	2.165352e-01
59	8.001516e-03	1.048696e-01	1.186210e-04	1.055308e-01
61	1.720717e-01	3.892213e-01	1.104630e-02	3.551846e-01
63	5.793239e-02	2.729032e-02	1.306316e-07	2.775555e-02
65	6.118995e-01	3.442456e-01	8.212230e-04	4.028195e-02
67	2.356273e-01	4.812486e-04	1.390134e-04	8.887922e-01
69	4.354448e-01	4.933623e-01	5.485842e-04	2.190510e-01
71	2.258196e-01	6.864803e-02	2.455363e-04	2.488975e-01
73	6.067246e-03	7.679903e-03	2.629487e-03	9.961035e-01
75	8.367062e-01	5.427012e-01	3.070588e-02	7.695548e-01
77	1.104041e-04	3.482570e-01	1.994550e-01	7.871713e-02
79	8.531762e-01	1.121278e-05	2.830366e-02	7.193111e-01
81	6.888301e-02	1.705936e-04	7.757936e-06	8.800450e-01
83	6.853431e-02	1.101334e-02	3.888230e-05	4.822087e-01
85	1.382449e-01	7.251819e-01	1.492975e-03	9.816256e-01
87	6.378522e-02	2.003939e-01	1.033107e-05	9.627470e-01
89	1.592942e-03	1.745307e-01	6.963494e-01	1.761379e-01
91	4.718129e-01	9.495398e-01	8.379505e-01	5.689812e-01
93	7.572522e-03	7.697627e-01	7.210547e-01	6.228407e-01
95	4.348427e-01	5.696695e-01	9.514624e-01	1.123493e-01

	NatNCBContrastRatioCorOGT	aa
1	6.018608e-07	KN
3	8.395528e-01	Y*
5	6.966561e-01	DE
7	3.115816e-02	QH
9	4.777845e-07	MI
11	1.874464e-01	LF
13	3.526369e-02	V
15	2.505372e-01	L
17	2.680590e-06	RS
19	5.346556e-01	CW*
21	1.339400e-05	G
23	5.766574e-05	R
25	4.824920e-05	T
27	5.982140e-03	S
29	8.749332e-06	A
31	1.105544e-02	P
33	1.305087e-04	KQE*
35	7.909213e-07	LIV
37	9.738137e-01	RG*
39	6.009548e-02	APST
41	6.999796e-03	NDHY
43	4.079177e-01	LFIV
45	3.478487e-01	RCSG
47	5.758868e-05	APST
49	1.353315e-04	KQE*
51	1.109976e-02	LMV
53	9.639199e-01	RGW
55	1.499449e-01	APST
57	1.877018e-02	NDHY
59	3.045821e-02	LFIV
61	7.027379e-01	RCSG
63	1.468648e-01	APST
65	6.008007e-01	
67	9.707087e-04	
69	8.750995e-01	
71	1.359979e-02	
73	1.512844e-02	
75	3.544806e-01	
77	4.339869e-01	
79	1.858842e-05	
81	4.248930e-04	
83	4.527972e-02	
85	9.091185e-01	
87	3.485706e-01	
89	1.694426e-01	
91	9.200534e-01	
93	6.875425e-01	
95	5.429196e-01	

Archaea: Dinucleotide combination composition

	sk	base1	base2	positions	NatFreq	NCBFreq	ShufflNatFreq	NatNCBRatio
1	A	R	R	12	3.13	2.37	3.13	1.32
3	A	Y	R	12	1.99	0.96	2.01	2.07
5	A	R	Y	12	1.56	1.56	1.56	1.00
7	A	Y	Y	12	1.13	1.16	1.14	0.97
9	A	R	R	23	2.91	1.71	2.93	1.70
11	A	Y	R	23	2.56	1.20	2.58	2.13
13	A	R	Y	23	2.34	1.64	2.35	1.43
15	A	Y	Y	23	3.02	0.58	3.03	5.21
17	A	R	R	31	4.39	1.11	3.21	3.95
19	A	Y	R	31	3.29	1.82	2.08	1.81
21	A	R	Y	31	1.87	1.21	1.12	1.55
23	A	Y	Y	31	1.97	0.86	2.04	2.29

	NatContrast	NCBContrast	ShufflNatContrast	NatNCBContrastRatio	NatFreqCorOGT
1	1.12	1.10	1.12	1.02	1.342258e-01
3	0.76	0.81	0.76	0.94	6.781202e-03
5	0.89	0.90	0.89	0.99	1.993954e-01
7	1.22	1.17	1.22	1.04	2.025185e-02
9	1.05	1.02	1.05	1.03	9.118437e-04
11	0.95	0.98	0.96	0.97	5.872277e-02
13	0.95	0.98	0.95	0.97	3.632089e-06
15	1.04	1.02	1.05	1.02	1.611469e-01
17	1.02	1.00	1.00	1.02	5.354815e-04
19	0.98	1.00	1.00	0.98	1.453220e-04
21	0.96	0.99	1.00	0.97	8.354718e-01
23	1.05	1.02	1.00	1.03	2.384783e-01

	NCBFreqCorOGT	NatContrastCorOGT	NCBContrastCorOGT	ShufflNatContrastCorOGT
1	1.096431e-01	0.005536433	3.215310e-01	0.005560377
3	8.695974e-02	0.008148541	9.983719e-01	0.008023834
5	1.993871e-01	0.015429331	6.133363e-01	0.014789947
7	1.081877e-04	0.022439775	7.558200e-01	0.020967845
9	3.317001e-04	0.186689600	1.279445e-06	0.185778159
11	2.482973e-01	0.168113088	2.390311e-06	0.189511637
13	1.325002e-08	0.068669174	3.890583e-07	0.064460041
15	1.898107e-01	0.062600548	7.820447e-07	0.067408227
17	4.789799e-02	0.044704901	2.978045e-01	0.187042996
19	7.465255e-07	0.023237795	2.888851e-01	0.077057535
21	5.081739e-06	0.034960156	2.980229e-01	0.480632671
23	3.607844e-01	0.016012565	2.607313e-01	0.688518200

	NatNCBContrastRatioCorOGT
1	1.902525e-05
3	5.395359e-06
5	6.430116e-06
7	1.459751e-06
9	4.671664e-01
11	6.047991e-01
13	9.036781e-01
15	8.890137e-01
17	2.585130e-02
19	9.977088e-03
21	1.950084e-02
23	6.700231e-03

Archaea: Purine-Pyrimidine dinucleotide predictor

```
Natural R(ogt ~ fRR + fYY + fRY + fRY) =
Call:
lm(formula = ogt ~ fRR + fYY + fRY + fYR, data = data)

Residuals:
    Min      1Q  Median      3Q     Max 
-42.743 -17.239   1.431  18.246  43.952 

Coefficients: (1 not defined because of singularities)
            Estimate Std. Error t value Pr(>|t|)
(Intercept)    51380      34180   1.503    0.140
fRR           -51127      34207  -1.495    0.142
fYY           -51368      34165  -1.504    0.140
fRY          -102877      68355  -1.505    0.140
fYR               NA         NA      NA       NA

Residual standard error: 24.42 on 42 degrees of freedom
Multiple R-squared:  0.2253,  Adjusted R-squared:  0.1699 
F-statistic: 4.071 on 3 and 42 DF,  p-value: 0.0126

[1] 0.47
NCB R(ogt ~ fRR + fYY + fRY + fRY) =
Call:
lm(formula = ogt ~ fRR + fYY + fRY + fYR, data = data)

Residuals:
    Min      1Q  Median      3Q     Max 
-41.261 -20.032   0.536  20.583  38.023 

Coefficients: (1 not defined because of singularities)
            Estimate Std. Error t value Pr(>|t|)
(Intercept)    49019      32712   1.498    0.141
fRR           -48959      32738  -1.495    0.142
fYY           -47812      32784  -1.458    0.152
fRY           -98998      65343  -1.515    0.137
fYR               NA         NA      NA       NA

Residual standard error: 23.44 on 42 degrees of freedom
Multiple R-squared:  0.2861,  Adjusted R-squared:  0.2351 
F-statistic:  5.61 on 3 and 42 DF,  p-value: 0.002506

[1] 0.53
```

Bacteria: Nucleic composition

	sk	base	codon	NatFreq	NCBFreq	NatNCBRatio	NatCorOGT	NCBCorOGT
2	B	A	1	6.35	5.10	1.25	0.2449783517	5.739717e-01
4	B	T	1	3.41	1.45	2.35	0.8488175383	8.112778e-01
6	B	G	1	4.14	4.14	1.00	0.8580418204	8.580441e-01
8	B	C	1	5.77	2.71	2.13	0.1948128394	1.418659e-01
10	B	A	2	4.43	4.38	1.01	0.2488612542	2.344568e-01
12	B	T	2	1.96	1.96	1.00	0.0005989265	5.990051e-04
14	B	G	2	2.84	2.81	1.01	0.5987977367	7.972073e-01
16	B	C	2	3.56	3.53	1.01	0.0036551497	1.376396e-03
18	B	A	3	11.63	0.51	22.80	0.2452005274	4.440892e-16
20	B	T	3	12.54	0.60	20.90	0.4178565820	5.119857e-05
22	B	G	3	9.99	0.90	11.10	0.7168150743	2.090934e-01
24	B	C	3	14.04	0.59	23.80	0.6206649334	5.854260e-05

	NatNCBRatioCorOGT	aa
2	0.01032339	RKNMSTI
4	0.69051860	LFCSWY*
6	0.95767042	ADEGV
8	0.16170180	LRPQH
10	0.29869059	KNDQEHY*
12	0.47960958	LMFIV
14	0.03139426	RCSGW*
16	0.02554313	APST
18	0.36783585	ALRKPQSETGIV*
20	0.50589109	ALRNDFCPSTGHYIV
22	0.80198795	ALRKMPQSETGWV*
24	0.77559355	ALRNDFCPSTGHYIV

Bacteria: Nucleic combination composition

	sk	bases	codon	NatFreq	NatNCBRatio	NCBFreq	NatCorOGT	NCBCorOGT
2	B	A+T	1	9.59	1.49	6.42	4.835560e-01	6.165804e-01
4	B	A+G	1	2.90	1.56	1.86	4.184818e-03	4.991838e-02
6	B	A+C	1	1.49	0.53	2.82	9.515066e-01	6.993875e-01
8	B	T+G	1	1.49	0.53	2.82	9.515066e-01	6.993875e-01
10	B	T+C	1	2.90	1.56	1.86	4.184818e-03	4.991838e-02
12	B	G+C	1	9.59	1.49	6.42	4.835560e-01	6.165804e-01
14	B	A+T	2	6.20	1.01	6.16	5.939498e-02	5.532036e-02
16	B	A+G	2	1.82	1.03	1.77	4.583123e-02	9.972362e-03
18	B	A+C	2	1.55	1.01	1.54	8.404570e-04	8.792811e-05
20	B	T+G	2	1.55	1.01	1.54	8.404570e-04	8.792811e-05
22	B	T+C	2	1.82	1.03	1.77	4.583123e-02	9.972362e-03
24	B	G+C	2	6.20	1.01	6.16	5.939498e-02	5.532036e-02
26	B	A+T	3	23.80	54.09	0.44	8.889917e-01	9.671073e-03
28	B	A+G	3	3.24	2.75	1.18	4.625540e-09	5.259961e-05
30	B	A+C	3	3.54	8.23	0.43	6.303334e-02	5.667023e-03
32	B	T+G	3	3.54	8.23	0.43	6.303334e-02	5.667023e-03
34	B	T+C	3	3.24	2.75	1.18	4.625540e-09	5.259961e-05
36	B	G+C	3	23.80	54.09	0.44	8.889917e-01	9.671073e-03

	NatNCBRatioCorOGT	aa
2	0.0087493090	LRKNMFCSTWYI*
4	0.0021466826	ARKNMDSETGIV
6	0.0009738807	LRKNMPQSTHI
8	0.6421595644	ALDFCSEGWYV*
10	0.7164504862	LRFCPQSWHY*
12	0.3833511494	ALRDPQEGHV
14	0.2572372479	LKNMDFQEHYIV*
16	0.1802422170	RKNDCQSEGWHY*
18	0.1491528643	AKNDPQSETHY*
20	0.5439051988	LRMFCSGWIV*
22	0.5478132790	ALMFPSTIV
24	0.0404574338	ARCPSTGW*
26	0.5098608354	ALRKNDFCPQSETGHYIV*
28	0.3388894341	ALRKMPQSETGWIV*
30	0.3317162013	ALRKNDFCPQSETGHYIV*
32	0.4565690642	ALRKNMDFCPQSETGWHYIV*
34	0.4732437210	ALRNDFCPSTGHYIV
36	0.7965539972	ALRKNMDFCPQSETGWHYIV*

Bacteria: Dinucleotide composition

	sk	base1	base2	positions	NatFreq	NCBFreq	ShufflNatFreq	NatNCBRatio
2	B	A	A	12	3.60	3.60	3.60	1.00
4	B	T	A	12	0.73	0.68	0.73	1.07
6	B	G	A	12	0.87	0.87	0.88	1.00
8	B	C	A	12	1.13	1.13	1.13	1.00
10	B	A	T	12	2.09	2.09	2.08	1.00
12	B	T	T	12	2.71	0.74	2.71	3.66
14	B	G	T	12	0.84	0.84	0.84	1.00
16	B	C	T	12	2.50	0.56	2.50	4.46
18	B	A	G	12	1.15	0.42	1.15	2.74
20	B	T	G	12	0.36	0.34	0.37	1.06
22	B	G	G	12	1.16	1.16	1.16	1.00
24	B	C	G	12	2.41	1.14	2.41	2.11
26	B	A	C	12	0.56	0.56	0.57	1.00
28	B	T	C	12	0.64	0.56	0.64	1.14
30	B	G	C	12	2.66	2.66	2.66	1.00
32	B	C	C	12	0.99	0.99	0.99	1.00
34	B	A	A	23	4.52	1.39	4.52	3.25
36	B	T	A	23	4.10	0.46	4.12	8.91
38	B	G	A	23	1.43	0.84	1.43	1.70
40	B	C	A	23	2.42	0.88	2.42	2.75
42	B	A	T	23	4.07	0.95	4.08	4.28
44	B	T	T	23	4.59	0.82	4.58	5.60
46	B	G	T	23	2.01	0.48	2.01	4.19
48	B	C	T	23	2.55	0.88	2.55	2.90
50	B	A	G	23	2.25	1.39	2.25	1.62
52	B	T	G	23	3.21	0.35	3.21	9.17
54	B	G	G	23	2.03	1.05	2.03	1.93
56	B	C	G	23	3.91	0.89	3.91	4.39
58	B	A	C	23	2.41	0.95	2.41	2.54
60	B	T	C	23	4.19	0.82	4.19	5.11
62	B	G	C	23	3.95	0.48	3.96	8.23
64	B	C	C	23	4.50	0.88	4.50	5.11
66	B	A	A	31	5.00	1.30	4.43	3.85
68	B	T	A	31	4.83	1.38	4.75	3.50
70	B	G	A	31	1.64	1.12	1.31	1.46
72	B	C	A	31	2.59	1.38	2.16	1.88
74	B	A	T	31	2.17	0.32	2.61	6.78
76	B	T	T	31	3.20	0.49	2.85	6.53
78	B	G	T	31	0.83	0.23	0.84	3.61
80	B	C	T	31	1.53	0.48	1.37	3.19
82	B	A	G	31	3.77	1.04	3.44	3.62
84	B	T	G	31	4.07	1.01	3.65	4.03
86	B	G	G	31	4.83	1.28	4.64	3.77
88	B	C	G	31	7.10	1.00	6.23	7.10
90	B	A	C	31	1.39	0.70	1.47	1.99
92	B	T	C	31	1.53	0.58	1.79	2.64
94	B	G	C	31	4.24	0.90	3.64	4.71
96	B	C	C	31	4.02	0.58	4.65	6.93

	NatContrast	NCBContrast	ShufflNatContrast	NatNCBContrastRatio	NatFreqCorOGT
2	1.15	1.11	1.15	1.04	2.216785e-01
4	0.69	0.65	0.69	1.06	1.638520e-03
6	1.11	1.11	1.11	1.00	2.522664e-07
8	0.88	0.95	0.88	0.93	3.400023e-08
10	1.11	1.08	1.11	1.03	4.677832e-01
12	1.45	1.49	1.45	0.97	9.301709e-01
14	0.67	0.67	0.67	1.00	2.086104e-04
16	1.10	1.09	1.10	1.01	3.767597e-01
18	0.70	0.87	0.71	0.80	1.039821e-03
20	0.81	0.77	0.81	1.05	2.542355e-02
22	1.15	1.17	1.14	0.98	9.636896e-01
24	1.21	1.05	1.21	1.15	7.175602e-02
26	0.91	0.87	0.91	1.05	4.267662e-05
28	0.96	0.97	0.96	0.99	3.073546e-03
30	1.17	1.15	1.17	1.02	1.508403e-02
32	0.85	0.91	0.85	0.93	7.568004e-01
34	1.50	1.05	1.50	1.43	2.872761e-01
36	0.76	0.99	0.76	0.77	3.969401e-01
38	0.68	0.92	0.68	0.74	1.441535e-06
40	0.95	1.02	0.95	0.93	6.104440e-01
42	1.12	0.98	1.12	1.14	1.634011e-01
44	1.08	1.01	1.08	1.07	4.922018e-01
46	1.00	0.98	1.00	1.02	5.750342e-03
48	0.77	1.01	0.77	0.76	3.847503e-01
50	0.89	0.99	0.89	0.90	2.078082e-01
52	1.18	0.98	1.18	1.20	6.975260e-01
54	0.81	1.11	0.81	0.73	3.284915e-03
56	1.05	0.96	1.05	1.09	3.346682e-01
58	0.76	0.98	0.76	0.78	1.926893e-01
60	0.92	1.01	0.92	0.91	7.911859e-01
62	1.33	0.98	1.33	1.36	3.298578e-02
64	1.13	1.01	1.13	1.12	5.237713e-01
66	1.22	1.00	1.14	1.22	3.726814e-02
68	0.93	0.98	1.12	0.95	3.153407e-01
70	0.88	1.01	0.93	0.87	1.576145e-02
72	1.01	0.98	0.91	1.03	4.753938e-01
74	1.06	0.97	1.13	1.09	8.832872e-01
76	1.29	1.03	1.11	1.25	7.592129e-01
78	0.79	0.98	0.94	0.81	7.197994e-01
80	0.98	1.03	0.92	0.95	4.922213e-01
82	0.90	1.00	0.94	0.90	2.322646e-01
84	1.02	1.01	0.94	1.01	2.080343e-01
86	0.98	0.99	1.03	0.99	1.643421e-01
88	1.06	1.01	1.04	1.05	4.469057e-01
90	0.84	1.00	0.86	0.84	4.820261e-01
92	0.86	0.99	0.88	0.87	4.290688e-01
94	1.29	1.02	1.07	1.26	1.161476e-01
96	0.92	0.99	1.09	0.93	7.401897e-01

	NCBFreqCorOGT	NatContrastCorOGT	NCBContrastCorOGT	ShufflNatContrastCorOGT
2	2.216851e-01	5.601284e-01	2.067778e-01	5.124302e-01
4	4.102490e-04	1.198097e-03	7.849055e-12	1.217857e-03
6	2.522150e-07	5.174384e-04	4.496170e-04	4.296813e-04
8	3.400868e-08	1.821056e-06	2.148251e-12	1.993551e-06
10	4.677763e-01	1.913138e-03	1.212633e-02	1.979445e-03
12	2.513635e-02	2.489938e-01	7.805143e-01	2.771490e-01
14	2.086139e-04	6.206782e-03	6.205942e-03	6.332226e-03
16	5.444067e-01	3.083828e-03	3.342126e-01	3.289966e-03
18	4.159605e-02	4.480105e-04	1.149592e-01	4.418444e-04
20	6.266472e-03	1.939040e-01	4.031091e-04	1.818510e-01
22	9.636709e-01	5.433994e-01	8.345065e-01	5.612733e-01
24	3.489338e-01	3.121245e-05	6.497647e-11	3.794182e-05
26	4.268458e-05	2.510744e-01	9.846456e-01	2.398396e-01
28	1.097520e-07	9.709413e-01	2.462084e-01	9.970356e-01
30	1.508495e-02	1.298624e-05	6.101015e-06	1.278901e-05
32	7.568061e-01	3.710608e-06	0.000000e+00	4.268755e-06
34	4.518938e-03	2.904529e-01	1.156208e-04	2.912515e-01
36	6.182346e-05	6.677612e-01	3.603756e-06	6.883386e-01
38	5.296444e-01	4.935896e-04	4.423368e-01	5.199542e-04
40	1.461453e-03	1.941817e-02	4.973226e-15	2.135854e-02
42	1.756933e-01	3.597710e-03	5.417279e-06	3.638922e-03
44	2.854359e-03	4.748518e-05	3.963451e-09	4.228835e-05
46	1.643502e-01	1.941178e-03	6.537839e-01	2.015773e-03
48	1.373994e-03	1.612895e-02	1.883149e-05	1.594334e-02
50	4.517918e-03	5.798503e-01	2.864894e-05	5.671879e-01

52	9.857486e-01	2.482277e-04	5.707000e-06	2.580312e-04
54	9.500539e-01	8.473468e-07	8.288619e-01	7.480079e-07
56	1.153170e-03	2.702367e-01	1.438280e-01	2.583906e-01
58	1.861768e-01	1.042299e-04	6.950806e-06	1.198680e-04
60	3.153869e-03	4.914323e-01	9.632273e-09	4.689238e-01
62	1.757894e-01	1.421698e-06	7.106789e-01	1.200758e-06
64	1.567660e-03	4.856827e-01	2.972694e-05	5.067302e-01
66	9.646544e-02	1.838603e-03	1.754465e-05	1.342075e-01
68	7.565883e-01	1.879474e-01	3.887770e-04	8.559278e-01
70	1.285830e-01	9.563905e-02	6.127116e-04	9.319135e-01
72	7.584536e-01	2.582999e-01	1.370102e-04	2.457810e-01
74	1.806080e-01	1.087817e-03	9.465031e-04	3.617807e-01
76	9.814856e-01	1.480293e-03	1.042145e-04	7.048315e-01
78	7.154515e-01	2.172308e-01	8.045582e-04	7.596147e-01
80	9.819071e-01	8.756142e-01	1.345126e-04	3.187902e-01
82	1.065546e-01	4.857554e-01	1.486659e-01	3.812843e-01
84	2.371111e-01	3.965708e-02	5.496577e-03	4.663745e-01
86	2.987868e-01	1.389197e-03	4.326949e-03	8.467556e-01
88	2.549612e-01	3.244783e-01	6.218839e-03	7.096088e-02
90	2.404245e-01	4.901059e-02	2.631685e-06	2.752246e-01
92	2.254019e-01	1.499745e-02	4.094826e-09	8.069414e-02
94	4.266987e-02	1.703956e-04	4.933313e-10	4.923968e-01
96	2.496749e-01	4.164198e-03	2.537958e-09	5.708054e-01

	NatNCBContrastRatioCorOGT	aa
2	2.868233e-02	KN
4	7.262128e-01	Y*
6	3.017452e-01	DE
8	1.111437e-01	QH
10	2.212236e-02	MI
12	4.231513e-01	LF
14	3.573839e-01	V
16	1.044241e-02	L
18	2.969730e-04	RS
20	4.023658e-01	CW*
22	3.114530e-02	G
24	3.437097e-08	R
26	2.152354e-03	T
28	3.411614e-01	S
30	2.651059e-02	A
32	2.041543e-01	P
34	1.388700e-01	KQE*
36	4.555881e-01	LIV
38	1.404338e-04	RG*
40	6.950762e-02	APST
42	1.254004e-02	NDHY
44	9.043146e-04	LFIV
46	6.016935e-03	RCSG
48	5.516977e-02	APST
50	2.647737e-01	KQE*
52	8.264819e-02	LMV
54	7.287579e-06	RGW
56	3.778430e-01	APST
58	5.366021e-07	NDHY
60	8.133978e-01	LFIV
62	2.882543e-07	RCSG
64	8.401550e-01	APST
66	4.504244e-03	
68	2.587223e-01	
70	2.434926e-01	
72	7.114238e-01	
74	1.254757e-03	
76	4.686508e-03	
78	3.943751e-01	
80	5.009004e-01	
82	5.711825e-01	
84	7.836682e-02	
86	2.326289e-03	
88	4.381121e-01	
90	1.081899e-01	
92	6.790402e-02	
94	1.621921e-03	
96	3.343268e-02	

Bacteria: Dinucleotide combination composition

	sk	base1	base2	positions	NatFreq	NCBFreq	ShufflNatFreq	NatNCBRatio
2	B	R	R	12	4.06	2.74	4.05	1.48
4	B	Y	R	12	2.61	1.35	2.62	1.93
6	B	R	Y	12	1.74	1.74	1.75	1.00
8	B	Y	Y	12	0.92	0.98	0.92	0.94
10	B	R	R	23	3.36	1.43	3.36	2.35
12	B	Y	R	23	2.46	1.51	2.46	1.63
14	B	R	Y	23	2.42	1.14	2.42	2.12
16	B	Y	Y	23	2.68	0.55	2.68	4.87
18	B	R	R	31	4.53	1.20	3.13	3.78
20	B	Y	R	31	2.75	1.47	1.64	1.87
22	B	R	Y	31	2.76	1.33	1.14	2.08
24	B	Y	Y	31	2.52	0.76	2.42	3.32

	NatContrast	NCBContrast	ShufflNatContrast	NatNCBContrastRatio	NatFreqCorOGT
2	1.06	1.08	1.06	0.98	2.093992e-03
4	0.90	0.86	0.90	1.05	7.119487e-04
6	0.94	0.93	0.94	1.01	1.839295e-02
8	1.09	1.12	1.09	0.97	6.395951e-01
10	0.98	1.02	0.98	0.96	1.047651e-11
12	1.02	0.99	1.02	1.03	2.586308e-01
14	1.02	0.98	1.02	1.04	2.014394e-16
16	0.98	1.01	0.98	0.97	7.591540e-01
18	0.97	1.00	1.00	0.97	2.783289e-10
20	1.02	1.00	1.00	1.02	1.830804e-13
22	1.03	1.00	1.00	1.03	2.229667e-03
24	0.98	1.01	1.00	0.97	9.829766e-01

	NCBFreqCorOGT	NatContrastCorOGT	NCBContrastCorOGT	ShufflNatContrastCorOGT
2	4.282435e-03	2.278992e-03	1.300839e-02	2.459228e-03
4	1.644169e-02	3.604649e-04	5.508328e-03	3.679084e-04
6	1.839854e-02	1.486976e-03	6.466280e-03	1.535893e-03
8	6.560287e-01	2.640548e-04	2.968215e-03	2.588668e-04
10	3.201395e-11	9.811478e-07	1.643130e-14	8.798785e-07
12	1.107644e-02	3.995991e-07	1.162774e-14	3.569257e-07
14	4.656945e-04	4.940223e-08	2.069310e-15	4.115090e-08
16	1.936910e-01	1.656203e-08	1.332268e-15	1.386819e-08
18	1.099463e-10	4.856478e-06	4.017743e-07	9.085734e-01
20	2.818399e-02	5.176299e-07	1.556052e-07	1.361115e-01
22	8.361745e-02	7.441153e-07	3.593269e-07	8.341944e-01
24	6.839819e-02	3.922179e-08	6.499155e-08	1.266726e-01

	NatNCBContrastRatioCorOGT
2	6.809084e-04
4	5.948165e-06
6	1.510529e-04
8	2.316641e-05
10	3.028827e-04
12	1.715123e-04
14	5.803502e-05
16	6.071797e-05
18	1.498437e-05
20	1.244201e-06
22	1.304134e-06
24	1.227672e-07

```
Bacteria: Purine-Pyrimidine dinucleotide predictor

Natural R(ogt ~ fRR + fYY + fRY + fRY) =
Call:
lm(formula = ogt ~ fRR + fYY + fRY + fYR, data = data)

Residuals:
    Min      1Q  Median      3Q     Max
-32.366  -9.345  -1.505  13.724  30.919

Coefficients: (1 not defined because of singularities)
            Estimate Std. Error t value Pr(>|t|)
(Intercept)   -18079      31729  -0.570    0.570
fRR            18305      31748   0.577    0.566
fYY            18136      31732   0.572    0.569
fRY            36014      63445   0.568    0.572
fYR               NA         NA      NA       NA

Residual standard error: 15.31 on 77 degrees of freedom
Multiple R-squared:  0.3336,  Adjusted R-squared:  0.3076
F-statistic: 12.85 on 3 and 77 DF,  p-value: 6.851e-07

[1] 0.58
NCB R(ogt ~ fRR + fYY + fRY + fRY) =
Call:
lm(formula = ogt ~ fRR + fYY + fRY + fYR, data = data)

Residuals:
    Min      1Q  Median      3Q     Max
-29.635  -9.380  -2.908  11.441  35.921

Coefficients: (1 not defined because of singularities)
            Estimate Std. Error t value Pr(>|t|)
(Intercept)   -17505      32884  -0.532    0.596
fRR            17975      32934   0.546    0.587
fYY            17605      32872   0.536    0.594
fRY            34510      65733   0.525    0.601
fYR               NA         NA      NA       NA

Residual standard error: 15.65 on 77 degrees of freedom
Multiple R-squared:  0.303,   Adjusted R-squared:  0.2758
F-statistic: 11.16 on 3 and 77 DF,  p-value: 3.691e-06

[1] 0.55
```
