# Supplementary material for: Molecular mechanisms of adaptation emerging from the physics and evolution of nucleic acids and proteins
Source: Nucleic Acids Res. 2013 Dec 25;42(5):2879–92. doi: 10.1093/nar/gkt1336 (PMC3950714; doi:10.1093/nar/gkt1336)
Supplement: Supplementary Data [file supp_gkt1336_nar-02158-n-2013-File008.pdf]

Supplementary File 6

(G+C)3 in Archaea and Bacteria, in Aerobia and Anaerobic genomes, in Mesophiles, Thermophiles, and Hyperthermophiles. T-tests for pairwise comparisons.

Overall GC3 summary. Natural and NCB sequences.

	sk	oxygen	thermoclass	sequence	N	w_mean_f	sd_f
1	A	Aerobic	hyperthermophile	nat	4	36.96191	18.93989401
2	A	Aerobic	hyperthermophile	ncb	4	50.07183	0.46961786
3	A	Aerobic	mesophile	nat	2	77.37523	0.59249770
4	A	Aerobic	mesophile	ncb	2	50.71152	0.07739629
5	A	Aerobic	thermophile	nat	2	46.32041	12.19056904
6	A	Aerobic	thermophile	ncb	2	50.38832	0.47148211
7	A	Anaerobic	hyperthermophile	nat	12	51.74242	17.86303455
8	A	Anaerobic	hyperthermophile	ncb	12	50.26167	0.34611080
9	A	Anaerobic	mesophile	nat	13	42.00967	20.17090752
10	A	Anaerobic	mesophile	ncb	13	50.22882	0.32175409
11	A	Anaerobic	thermophile	nat	3	71.37586	16.48682490
12	A	Anaerobic	thermophile	ncb	3	50.69433	0.12166782
13	B	Aerobic	hyperthermophile	nat	1	48.07827	NA
14	B	Aerobic	hyperthermophile	ncb	1	50.14525	NA
15	B	Aerobic	mesophile	nat	62	70.97228	23.72179736
16	B	Aerobic	mesophile	ncb	62	50.86975	0.39532725
17	B	Aerobic	thermophile	nat	5	81.46551	12.68591217
18	B	Aerobic	thermophile	ncb	5	50.81508	0.08196935
19	B	Anaerobic	hyperthermophile	nat	2	52.43418	0.14263254
20	B	Anaerobic	hyperthermophile	ncb	2	50.42405	0.04182169
21	B	Anaerobic	mesophile	nat	24	50.51896	21.71066182
22	B	Anaerobic	mesophile	ncb	24	50.63654	0.41907935
23	B	Anaerobic	thermophile	nat	11	46.80690	17.66128754
24	B	Anaerobic	thermophile	ncb	11	50.34280	0.36521972

Natural sequences, GC3. Oxygen tolerance summary

	sk	oxygen	N	w_mean_f	sd_f
1	A	Aerobic	8	51.50434	21.40989
2	A	Anaerobic	29	47.91637	20.30903
3	B	Aerobic	71	71.42962	23.13256
4	B	Anaerobic	38	49.49695	19.58345

Domain of Life summary, GC3. Natural and NCB sequences.

	sk	sequence	N	w_mean_f	sd_f
1	A	nat	46	51.16033	20.1136173
2	A	ncb	46	50.35074	0.3542847
3	B	nat	197	59.38112	23.7438664
4	B	ncb	197	50.75624	0.5023026

Archaea GC3: Natural vs NCB

Difference	Mean.x	Mean.y	Std. Err	p.value
0.8400116	51.1603313	50.3203197	2.8324413	0.7681599

Bacteria GC3: Natural vs NCB

Difference	Mean.x	Mean.y	Std. Err	p.value
8.745222	59.381124	50.635902	1.613359	1.732839e-07

```
Oxygen tolerance summary: Natural vs NCB sequences
     oxygen sequence  N w_mean_f       sd_f
1   Aerobic      nat 79 70.13127 23.3136119
2   Aerobic      ncb 79 50.83610  0.4078330
3 Anaerobic      nat 67 48.93681 19.7579662
4 Anaerobic      ncb 67 50.45605  0.3999003


Difference between Aerobic Natural and NCB GC3 frequencies
Difference     Mean.x     Mean.y   Std. Err
 19.391657  70.131274  50.739617   2.377954
     p.value
4.695799e-12


Difference between Anaerobic Natural and NCB GC3 frequencies
Difference     Mean.x     Mean.y   Std. Err
 -1.479187  48.936815  50.416002   2.301082
  p.value
0.5225642


Difference between Aerobic and Anaerobic Natural GC3 frequencies
Difference     Mean.x     Mean.y   Std. Err
 21.194460  70.131274  48.936815   3.308336
     p.value
1.996515e-09
```

```
Mesophiles:
  sk sequence   N w_mean_f       sd_f
1  A      nat  18 50.61418 24.1823830
2  A      ncb  18 50.34244  0.3530842
3  B      nat 160 60.19478 24.6011403
4  B      ncb 160 50.77342  0.5309123


Archaea Mesophile GC3: Nat vs NCB
Difference     Mean.x     Mean.y   Std. Err
 0.3322471 50.6141784 50.2819312  5.0826202
  p.value
0.9486423
[1] "\n\nBacteria Mesophile GC3: Nat vs NCB\n"
Difference     Mean.x     Mean.y   Std. Err
  9.576355  60.194784  50.618429   1.840414
     p.value
5.955473e-07


Natural GC3 frequencies in Mesophiles: Archaea vs Bacteria
Difference     Mean.x     Mean.y   Std. Err
 -9.580606  50.614178  60.194784   5.404901
   p.value
0.09033335


Mesophiles:
     oxygen sequence  N w_mean_f       sd_f
1   Aerobic      nat 64 71.12517 23.4471079
2   Aerobic      ncb 64 50.86597  0.3892967
3 Anaerobic      nat 37 47.90950 21.4760139
4 Anaerobic      ncb 37 50.51151  0.4380579


Mesophiles GC3: Aerobic Natural vs NCB
Difference     Mean.x     Mean.y   Std. Err
 20.325163  71.125174  50.800012   2.601963
     p.value
7.595591e-11


Mesophiles GC3: Anaerobic Natural vs NCB
Difference     Mean.x     Mean.y   Std. Err
 -2.483662  47.909496  50.393158   3.312484
  p.value
0.4582498


Mesophiles GC3: Aerobic vs Anaerobic Natural
Difference     Mean.x     Mean.y   Std. Err
 23.215678  71.125174  47.909496   4.211342
     p.value
4.494256e-07
```

```
Thermophiles summary:
  sk sequence  N w_mean_f       sd_f
1  A      nat  7 57.96882 17.2635196
2  A      ncb  7 50.50073  0.2942904
3  B      nat 20 60.79974 22.9029027
4  B      ncb 20 50.58797  0.3898416


Archaea Thermophile GC3: Nat vs NCB
Difference     Mean.x     Mean.y   Std. Err
  7.508539  57.968824  50.460285   6.164588
 p.value
0.268913


Bacteria Thermophile GC3: Nat vs NCB
Difference     Mean.x     Mean.y   Std. Err
 10.261486  60.799741  50.538255   4.681193
   p.value
0.04102293


Natural GC3 frequencies in Thermophiles: Archaea vs Bacteria
Difference     Mean.x     Mean.y   Std. Err
 -2.830917  57.968824  60.799741   7.739311
  p.value
0.7201952


Thermophiles summary:
     oxygen sequence  N w_mean_f       sd_f
1   Aerobic      nat  7 74.30950 21.4826492
2   Aerobic      ncb  7 50.72818  0.3065179
3 Anaerobic      nat 14 50.59250 20.1553644
4 Anaerobic      ncb 14 50.39696  0.3680422


Thermophiles, Aerobic, GC3: Natural vs NCB
Difference     Mean.x     Mean.y   Std. Err
 23.610993  74.309496  50.698504   6.814015
   p.value
0.01337482


Thermophiles, Anaerobic, GC3: Natural vs NCB
Difference     Mean.x     Mean.y   Std. Err
 0.2431481 50.5925009 50.3493528  4.9214898
  p.value
0.9613468


Thermophiles, Natural GC3: Aerobic vs Anaerobic
Difference     Mean.x     Mean.y   Std. Err
 23.716995  74.309496  50.592501   8.404392
   p.value
0.01504285
```

```
HyperThermophiles summary:
  sk sequence  N w_mean_f       sd_f
1  A      nat 19 49.18978 17.6003542
2  A      ncb 19 50.27174  0.3700830
3  B      nat  3 51.15257  2.5163530
4  B      ncb  3 50.34202  0.1638139


Archaea HyperThermophile GC3: Nat VS NCB
Difference     Mean.x     Mean.y   Std. Err
 -1.032798  49.189778  50.222576   3.835568
  p.value
0.7907826


Bacteria HyperThermophile GC3: Nat VS NCB
Difference     Mean.x     Mean.y   Std. Err
 0.8276223 51.1525681 50.3249457  1.1496584
  p.value
0.5457704


Natural frequencies GC3 in HyperThermophiles: Archaea vs Bacteria
Difference     Mean.x     Mean.y   Std. Err
  -1.96279   49.18978   51.15257    4.00258
  p.value
0.6292196


HyperThermophiles:
     oxygen sequence  N w_mean_f       sd_f
1   Aerobic      nat  5 38.63596 16.8333520
2   Aerobic      ncb  5 50.08288  0.4067095
3 Anaerobic      nat 14 51.85280 16.4632045
4 Anaerobic      ncb 14 50.28758  0.3284151


HyperThermophiles GC3: Aerobic Natural vs NCB
Difference     Mean.x     Mean.y   Std. Err
 -11.47100   38.63596   50.10696    6.32398
  p.value
0.1438192


HyperThermophiles GC3: Anaerobic Natural vs NCB
Difference     Mean.x     Mean.y   Std. Err
  1.641097  51.852804  50.211708   3.927830
  p.value
0.6828964


HyperThermophiles GC3: Aerobic vs Anaerobic Natural
Difference     Mean.x     Mean.y   Std. Err
-13.216840  38.635965  51.852804   7.442578
  p.value
0.1170088
```

Differences in codon usage:

	aa	codon	Aero-Anaero diff	Aero-Anaero p-val	Bact-Arch diff	Bact-Arch p-val
1	A	GCT	-11.96	2.543097e-10	-5.60	3.670495e-03
2	A	GCC	10.12	4.186873e-05	8.51	7.990874e-05
3	A	GCA	-13.62	2.894147e-10	-11.83	3.218219e-06
4	A	GCG	15.46	0.000000e+00	8.92	3.919487e-05
5	L	TTA	-8.48	2.290187e-03	-1.93	4.792244e-01
6	L	TTG	-1.47	1.776823e-01	4.35	3.006180e-07
7	L	CTT	-11.19	3.996803e-15	-8.29	5.441127e-07
8	L	CTC	2.35	2.683872e-01	-5.85	1.141025e-02
9	L	CTA	-2.11	1.116711e-02	-4.27	9.892245e-05
10	L	CTG	20.91	4.304865e-10	16.00	4.600786e-10
11	R	CGT	-0.99	5.433620e-01	11.16	0.000000e+00
12	R	CGC	26.74	0.000000e+00	26.73	0.000000e+00
13	R	CGA	-0.62	2.927820e-01	1.30	1.442162e-01
14	R	CGG	5.04	1.299357e-02	4.41	5.054106e-02
15	R	AGA	-17.68	4.457862e-08	-17.35	1.649056e-07
16	R	AGG	-12.49	5.805334e-08	-26.25	1.141132e-11
17	K	AAA	-17.16	6.441416e-06	3.11	3.754940e-01
18	K	AAG	17.16	6.441416e-06	-3.11	3.754940e-01
19	N	AAT	-16.01	6.049569e-06	-0.35	9.161458e-01
20	N	AAC	16.01	6.049569e-06	0.35	9.161458e-01
21	M	ATG	0.00	NaN	0.00	NaN
22	D	GAT	-19.99	1.633181e-08	0.01	9.977292e-01
23	D	GAC	19.99	1.633181e-08	-0.01	9.977292e-01
24	F	TTT	-20.70	6.426050e-07	1.02	7.731542e-01
25	F	TTC	20.70	6.426050e-07	-1.02	7.731542e-01
26	C	TGT	-19.01	7.488240e-08	-8.64	8.332782e-03
27	C	TGC	19.01	7.488240e-08	8.64	8.332782e-03
28	P	CCT	-12.45	6.765561e-10	-5.29	7.832210e-03
29	P	CCC	3.62	5.409003e-02	0.54	7.613872e-01
30	P	CCA	-8.94	1.358421e-04	-8.58	7.424112e-04
31	P	CCG	17.76	1.097671e-10	13.33	2.892910e-06
32	Q	CAA	-7.50	4.645262e-02	9.38	1.077552e-02
33	Q	CAG	7.50	4.645262e-02	-9.38	1.077552e-02
34	S	TCT	-7.61	1.708663e-07	-2.12	8.652330e-02
35	S	TCC	2.02	1.612605e-01	0.00	9.974766e-01
36	S	TCA	-8.84	4.273902e-09	-7.47	2.194369e-05
37	S	TCG	11.81	7.795853e-11	5.51	1.163872e-03
38	S	AGT	-5.85	3.129974e-06	-0.45	7.106579e-01
39	S	AGC	8.46	4.756720e-08	4.52	2.156924e-03
40	E	GAA	-12.12	1.228387e-04	7.23	3.791790e-02
41	E	GAG	12.12	1.228387e-04	-7.23	3.791790e-02
42	T	ACT	-10.16	3.504844e-07	-5.97	2.352170e-03
43	T	ACC	14.28	1.474895e-06	12.11	2.332155e-07
44	T	ACA	-13.37	4.771509e-09	-9.72	2.213172e-05
45	T	ACG	9.25	6.187169e-07	3.59	7.045199e-02
46	G	GGT	-6.58	9.312825e-05	1.79	3.121469e-01
47	G	GGC	26.79	0.000000e+00	16.85	7.312656e-08
48	G	GGA	-18.12	3.197442e-14	-13.31	1.909090e-07
49	G	GGG	-2.09	7.493112e-02	-5.33	1.055364e-05
50	W	TGG	0.00	NaN	0.00	NaN
51	H	CAT	-12.18	1.142989e-04	5.17	1.298376e-01
52	H	CAC	12.18	1.142989e-04	-5.17	1.298376e-01
53	Y	TAT	-12.39	4.348973e-04	5.90	7.765163e-02
54	Y	TAC	12.39	4.348973e-04	-5.90	7.765163e-02
55	I	ATT	-7.33	5.234798e-03	7.08	3.428653e-03
56	I	ATC	25.65	1.739566e-08	16.90	1.516899e-05
57	I	ATA	-18.31	1.653516e-09	-23.98	2.287059e-12
58	V	GTT	-15.33	2.030154e-11	-8.06	8.546184e-04
59	V	GTC	12.34	2.203403e-07	2.84	2.701083e-01
60	V	GTA	-11.26	5.640161e-08	-6.82	4.409884e-04
61	V	GTG	14.26	4.097753e-10	12.04	5.593219e-09
62	*	TAG	0.82	5.790772e-01	-0.68	7.193295e-01
63	*	TGA	16.98	4.375765e-07	2.77	3.169199e-01
64	*	TAA	-17.81	9.546583e-08	-2.09	4.959709e-01
