# Supplementary material for: Molecular mechanisms of adaptation emerging from the physics and evolution of nucleic acids and proteins
Source: Nucleic Acids Res. 2013 Dec 25;42(5):2879–92. doi: 10.1093/nar/gkt1336 (PMC3950714; doi:10.1093/nar/gkt1336)
Supplement: Supplementary Data [file supp_gkt1336_nar-02158-n-2013-File009.pdf]

Supplementary File 7

One-sided t-tests for position-specific nucleic acid composition

Archaea: Nucleic composition (one-sided t-test vs 25%)

	sk	base	codon	NatFreq	NCBFreq
1	A	A	1	4.298026e-07	6.068036e-06
3	A	T	1	0.000000e+00	0.000000e+00
5	A	G	1	0.000000e+00	0.000000e+00
7	A	C	1	4.440892e-16	0.000000e+00
9	A	A	2	4.440892e-16	4.440892e-16
11	A	T	2	0.000000e+00	0.000000e+00
13	A	G	2	0.000000e+00	0.000000e+00
15	A	C	2	3.144240e-11	2.122391e-11
17	A	A	3	8.955521e-01	8.372381e-04
19	A	T	3	4.905122e-01	7.777708e-01
21	A	G	3	9.685444e-01	1.450904e-02
23	A	C	3	4.805761e-01	8.197232e-01

Archaea: Nucleic combination composition (one-sided t-test vs 50%)

	sk	bases	codon	NatFreq	NCBFreq
1	A	A+T	1	4.934865e-03	7.682741e-06
3	A	A+G	1	0.000000e+00	0.000000e+00
5	A	A+C	1	1.633883e-09	1.835874e-08
7	A	T+G	1	1.633883e-09	1.835874e-08
9	A	T+C	1	0.000000e+00	0.000000e+00
11	A	G+C	1	4.934865e-03	7.682741e-06
13	A	A+T	2	0.000000e+00	0.000000e+00
15	A	A+G	2	4.440892e-15	7.105427e-15
17	A	A+C	2	1.973488e-07	2.674242e-08
19	A	T+G	2	1.973488e-07	2.674242e-08
21	A	T+C	2	4.440892e-15	7.105427e-15
23	A	G+C	2	0.000000e+00	0.000000e+00
25	A	A+T	3	6.839515e-01	2.828453e-09
27	A	A+G	3	6.534510e-01	7.981999e-01
29	A	A+C	3	3.187196e-02	4.207549e-09
31	A	T+G	3	3.187196e-02	4.207549e-09
33	A	T+C	3	6.534510e-01	7.981999e-01
35	A	G+C	3	6.839515e-01	2.828453e-09

Archaea: Dinucleotide composition (one-sided t-test contrast vs 1.0)

	sk	base1	base2	positions	NatContrast	NCBContrast	ShufflNatContrast
1	A	A	A	12	1.262594e-01	2.392379e-03	1.422234e-01
3	A	T	A	12	3.685940e-14	0.000000e+00	3.108624e-14
5	A	G	A	12	0.000000e+00	0.000000e+00	0.000000e+00
7	A	C	A	12	0.000000e+00	0.000000e+00	0.000000e+00
9	A	A	T	12	1.598878e-03	1.682610e-11	1.388899e-03
11	A	T	T	12	9.687584e-12	0.000000e+00	9.361401e-12
13	A	G	T	12	0.000000e+00	0.000000e+00	0.000000e+00
15	A	C	T	12	7.216450e-14	0.000000e+00	5.728751e-14
17	A	A	G	12	7.857065e-01	1.001421e-13	7.759543e-01
19	A	T	G	12	9.325873e-15	0.000000e+00	1.110223e-14
21	A	G	G	12	4.086731e-12	5.089262e-13	4.875211e-12
23	A	C	G	12	2.443310e-07	1.305622e-13	2.664902e-07
25	A	A	C	12	7.006307e-06	4.581195e-07	7.994777e-06
27	A	T	C	12	7.710566e-08	4.025358e-06	7.304965e-08
29	A	G	C	12	8.891420e-01	7.832266e-01	9.308709e-01
31	A	C	C	12	7.630266e-05	1.433791e-07	7.488155e-05
33	A	A	A	23	7.800194e-01	1.441988e-05	8.314955e-01
35	A	T	A	23	2.204602e-03	7.091278e-01	2.273726e-03
37	A	G	A	23	9.031694e-01	0.000000e+00	9.285211e-01
39	A	C	A	23	9.939709e-07	4.132008e-04	8.657985e-07
41	A	A	T	23	2.054547e-01	2.306281e-03	1.903611e-01
43	A	T	T	23	3.719605e-06	2.297560e-06	4.529232e-06
45	A	G	T	23	8.181957e-03	2.474742e-01	8.815075e-03
47	A	C	T	23	2.779492e-01	9.625401e-01	2.888943e-01
49	A	A	G	23	7.533884e-03	1.886728e-01	7.821997e-03
51	A	T	G	23	8.490570e-02	2.205098e-08	9.339680e-02
53	A	G	G	23	1.444296e-08	6.683543e-14	1.450818e-08
55	A	C	G	23	4.736549e-06	1.816219e-02	4.991054e-06
57	A	A	C	23	6.203081e-01	2.144503e-03	6.259847e-01
59	A	T	C	23	4.204473e-01	1.390879e-06	3.982024e-01
61	A	G	C	23	3.411766e-01	2.591716e-01	3.268466e-01
63	A	C	C	23	5.484540e-01	9.916874e-01	5.243387e-01
65	A	A	A	31	4.062794e-05	1.750150e-02	6.589678e-01
67	A	T	A	31	9.882530e-07	7.519394e-09	4.226721e-01
69	A	G	A	31	6.131075e-02	2.646443e-05	4.033140e-01
71	A	C	A	31	1.532729e-01	3.812838e-09	5.353701e-01
73	A	A	T	31	5.213499e-01	1.161288e-04	2.371188e-01
75	A	T	T	31	5.652752e-01	1.256346e-05	1.778549e-01
77	A	G	T	31	6.087782e-04	1.293679e-02	1.334544e-01
79	A	C	T	31	2.707814e-05	1.405905e-05	2.371828e-02
81	A	A	G	31	2.071648e-03	8.343143e-02	3.663451e-02
83	A	T	G	31	4.192206e-07	1.907566e-02	4.748759e-01
85	A	G	G	31	5.526153e-01	4.249776e-01	9.456281e-01
87	A	C	G	31	3.068450e-04	2.307250e-02	3.714050e-01
89	A	A	C	31	3.671585e-02	1.511578e-06	2.806451e-01
91	A	T	C	31	3.841959e-01	2.381486e-05	3.729632e-01
93	A	G	C	31	1.168863e-02	7.329258e-02	1.931136e-02
95	A	C	C	31	1.871155e-04	9.691383e-06	4.698908e-01

Archaea: Dinucleotide combination composition (one-sided t-test contrast vs 1.0)

	sk	base1	base2	positions	NatContrast	NCBContrast	ShufflNatContrast
1	A	R	R	12	0.000000e+00	0.000000e+00	0.000000e+00
3	A	Y	R	12	0.000000e+00	0.000000e+00	0.000000e+00
5	A	R	Y	12	0.000000e+00	0.000000e+00	0.000000e+00
7	A	Y	Y	12	0.000000e+00	0.000000e+00	0.000000e+00
9	A	R	R	23	6.024540e-05	4.697591e-04	8.459725e-05
11	A	Y	R	23	5.995923e-05	3.611165e-04	7.340040e-05
13	A	R	Y	23	6.551935e-05	2.634306e-04	6.480516e-05
15	A	Y	Y	23	7.279326e-05	2.085869e-04	6.454060e-05
17	A	R	R	31	1.827251e-01	2.804966e-01	1.444874e-01
19	A	Y	R	31	2.440312e-02	9.150314e-03	3.858653e-01
21	A	R	Y	31	3.558078e-02	6.685324e-04	3.381458e-01
23	A	Y	Y	31	2.844905e-03	2.739928e-06	8.417425e-02

Bacteria: Nucleic composition (one-sided t-test vs 25%)

	sk	base	codon	NatFreq	NCBFreq
2	B	A	1	3.828463e-01	6.493143e-01
4	B	T	1	0.000000e+00	0.000000e+00
6	B	G	1	0.000000e+00	0.000000e+00
8	B	C	1	2.838840e-03	0.000000e+00
10	B	A	2	5.166316e-10	2.624689e-10
12	B	T	2	0.000000e+00	0.000000e+00
14	B	G	2	0.000000e+00	0.000000e+00
16	B	C	2	1.204423e-03	1.092797e-02
18	B	A	3	4.387815e-09	8.257395e-12
20	B	T	3	3.400452e-03	4.534426e-09
22	B	G	3	1.414372e-04	0.000000e+00
24	B	C	3	1.416577e-06	4.178053e-09

Bacteria: Nucleic combination composition (one-sided t-test vs 50%)

	sk	bases	codon	NatFreq	NCBFreq
2	B	A+T	1	0.000000e+00	0.000000e+00
4	B	A+G	1	0.000000e+00	0.000000e+00
6	B	A+C	1	0.000000e+00	0.000000e+00
8	B	T+G	1	0.000000e+00	0.000000e+00
10	B	T+C	1	0.000000e+00	0.000000e+00
12	B	G+C	1	0.000000e+00	0.000000e+00
14	B	A+T	2	0.000000e+00	0.000000e+00
16	B	A+G	2	0.000000e+00	0.000000e+00
18	B	A+C	2	0.000000e+00	0.000000e+00
20	B	T+G	2	0.000000e+00	0.000000e+00
22	B	T+C	2	0.000000e+00	0.000000e+00
24	B	G+C	2	0.000000e+00	0.000000e+00
26	B	A+T	3	8.215552e-06	0.000000e+00
28	B	A+G	3	0.000000e+00	4.044656e-09
30	B	A+C	3	7.234125e-01	0.000000e+00
32	B	T+G	3	7.234125e-01	0.000000e+00
34	B	T+C	3	0.000000e+00	4.044656e-09
36	B	G+C	3	8.215552e-06	0.000000e+00

Bacteria: Dinucleotide composition (one-sided t-test contrast vs 1.0)

	sk	base1	base2	positions	NatContrast	NCBContrast	ShufflNatContrast
2	B	A	A	12	0.000000e+00	4.688372e-07	0.000000e+00
4	B	T	A	12	0.000000e+00	0.000000e+00	0.000000e+00
6	B	G	A	12	0.000000e+00	0.000000e+00	0.000000e+00
8	B	C	A	12	1.961886e-06	3.534254e-03	1.831269e-06
10	B	A	T	12	0.000000e+00	3.976819e-13	0.000000e+00
12	B	T	T	12	0.000000e+00	0.000000e+00	0.000000e+00
14	B	G	T	12	0.000000e+00	0.000000e+00	0.000000e+00
16	B	C	T	12	4.722757e-05	0.000000e+00	5.010283e-05
18	B	A	G	12	0.000000e+00	2.819522e-12	0.000000e+00
20	B	T	G	12	8.881784e-16	0.000000e+00	8.881784e-16
22	B	G	G	12	0.000000e+00	0.000000e+00	0.000000e+00
24	B	C	G	12	4.822953e-05	1.077458e-02	4.628242e-05
26	B	A	C	12	5.073791e-04	0.000000e+00	5.737187e-04
28	B	T	C	12	2.766942e-01	3.827775e-01	2.889935e-01
30	B	G	C	12	0.000000e+00	0.000000e+00	0.000000e+00
32	B	C	C	12	9.882619e-06	1.887823e-12	1.227909e-05
34	B	A	A	23	0.000000e+00	1.177169e-11	0.000000e+00
36	B	T	A	23	0.000000e+00	3.277379e-02	0.000000e+00
38	B	G	A	23	3.315012e-04	0.000000e+00	3.830910e-04
40	B	C	A	23	3.937237e-01	9.439338e-12	3.932228e-01
42	B	A	T	23	8.236202e-05	5.151136e-02	7.638715e-05
44	B	T	T	23	6.485171e-01	5.446105e-04	6.422840e-01
46	B	G	T	23	3.318308e-03	2.604432e-02	3.304887e-03
48	B	C	T	23	0.000000e+00	3.370457e-09	2.220446e-16
50	B	A	G	23	7.772491e-07	2.120372e-02	7.606552e-07
52	B	T	G	23	0.000000e+00	3.442234e-04	0.000000e+00
54	B	G	G	23	3.403038e-10	0.000000e+00	2.480665e-10
56	B	C	G	23	3.712867e-01	0.000000e+00	3.630294e-01
58	B	A	C	23	0.000000e+00	4.224967e-02	0.000000e+00
60	B	T	C	23	3.924590e-05	5.901057e-04	3.611010e-05
62	B	G	C	23	0.000000e+00	5.463764e-02	0.000000e+00
64	B	C	C	23	6.522763e-04	4.722303e-09	6.744856e-04
66	B	A	A	31	6.507420e-01	6.531721e-01	4.073319e-03
68	B	T	A	31	0.000000e+00	9.103829e-14	7.493951e-01
70	B	G	A	31	4.736642e-04	2.146375e-05	4.117308e-01
72	B	C	A	31	1.953993e-14	4.107825e-14	4.888839e-01
74	B	A	T	31	4.337961e-01	0.000000e+00	5.897055e-02
76	B	T	T	31	2.761258e-11	0.000000e+00	9.645473e-01
78	B	G	T	31	1.598721e-14	5.091483e-12	4.497170e-01
80	B	C	T	31	2.369863e-05	0.000000e+00	8.595675e-01
82	B	A	G	31	1.311618e-07	7.495204e-01	2.814814e-01
84	B	T	G	31	8.814321e-04	2.370597e-06	5.317961e-01
86	B	G	G	31	5.018227e-03	3.160370e-06	5.556224e-01
88	B	C	G	31	1.867200e-01	1.325353e-06	5.103767e-01
90	B	A	C	31	5.046026e-01	2.710207e-01	3.246646e-01
92	B	T	C	31	1.565127e-01	1.417569e-01	6.305789e-02
94	B	G	C	31	2.664535e-15	7.669881e-08	1.065979e-01
96	B	C	C	31	3.997646e-07	1.020063e-01	5.373452e-01

Bacteria: Dinucleotide combination composition (one-sided t-test contrast vs 1.0)

	sk	base1	base2	positions	NatContrast	NCBContrast	ShufflNatContrast
2	B	R	R	12	0.000000e+00	0.000000e+00	0.000000e+00
4	B	Y	R	12	2.220446e-15	0.000000e+00	2.220446e-15
6	B	R	Y	12	0.000000e+00	0.000000e+00	0.000000e+00
8	B	Y	Y	12	1.998401e-14	0.000000e+00	2.575717e-14
10	B	R	R	23	8.736626e-03	1.915163e-09	8.318406e-03
12	B	Y	R	23	1.489969e-02	3.259722e-09	1.396736e-02
14	B	R	Y	23	1.463050e-02	3.053944e-09	1.461836e-02
16	B	Y	Y	23	2.601409e-02	4.986447e-09	2.548212e-02
18	B	R	R	31	5.104812e-04	1.246654e-01	4.960150e-01
20	B	Y	R	31	4.927463e-03	4.740847e-01	2.678716e-01
22	B	R	Y	31	1.645131e-02	3.843450e-02	5.384415e-01
24	B	Y	Y	31	1.064827e-01	5.042345e-05	2.501249e-01
