# Supplementary material for: Molecular mechanisms of adaptation emerging from the physics and evolution of nucleic acids and proteins
Source: Nucleic Acids Res. 2013 Dec 25;42(5):2879–92. doi: 10.1093/nar/gkt1336 (PMC3950714; doi:10.1093/nar/gkt1336)
Supplement: Supplementary Data [file supp_gkt1336_nar-02158-n-2013-File010.pdf]

Supplementary File 8

Position-independent nucleic acid composition, correlations with OGT, t-tests

Correlation between OGT and nucleic acid composition of DNA, RNA and ncDNA in Archaea

	sk	base	DNANat	DNANCB	tRNA	rRNA	ncDNA
1	A	A	0.9768928	0.51261033	7.850419e-10	9.215839e-14	0.5418530
3	A	T	0.8187515	0.06466334	7.027122e-17	4.900512e-16	0.5813254
5	A	G	0.2990355	0.49764063	4.087841e-13	0.000000e+00	0.5463846
7	A	C	0.3014059	0.24545690	2.096101e-13	1.248763e-09	0.5752836

Correlation between OGT and dinucleotide composition of DNA, RNA and ncDNA in Archaea

	sk	base1	base2	DNANat	DNANCB	DNAShuffledCodons	DNAShuffledCodonsNCB	DNANatContrast
1	A	A	A	5.104629e-01	9.471314e-01	5.804403e-01	8.927476e-01	6.585663e-02
3	A	T	A	7.121531e-02	1.836955e-01	1.934067e-01	2.970589e-01	1.172639e-06
5	A	G	A	2.877973e-01	3.082593e-02	5.784760e-02	6.808296e-02	1.259606e-01
7	A	C	A	2.681204e-04	9.117953e-10	9.214127e-05	1.226346e-11	8.520569e-03
9	A	A	T	3.100154e-01	2.253658e-01	4.166783e-01	2.526258e-01	1.097686e-04
11	A	T	T	9.672374e-01	2.985514e-02	8.849831e-01	3.059143e-02	7.238735e-01
13	A	G	T	2.045539e-02	2.767933e-03	6.515667e-04	1.273109e-03	9.367037e-01
15	A	C	T	1.202238e-02	4.625803e-03	1.751298e-01	1.467283e-02	5.803764e-07
17	A	A	G	1.036382e-10	3.633205e-04	2.865020e-09	4.263073e-03	3.420353e-11
19	A	T	G	1.229358e-01	4.639040e-01	7.682560e-01	8.380241e-01	1.977451e-05
21	A	G	G	9.408968e-02	2.393766e-01	2.081185e-01	3.831166e-01	6.624004e-01
23	A	C	G	2.181957e-01	6.644970e-01	2.770707e-01	8.965367e-01	5.430521e-02
25	A	A	C	4.745429e-02	3.823318e-07	4.228307e-02	2.659919e-07	2.255109e-01
27	A	T	C	3.824912e-02	7.247978e-01	2.570507e-02	6.573738e-01	4.980189e-02
29	A	G	C	7.376515e-01	9.405917e-01	7.468542e-01	9.457454e-01	9.363360e-01
31	A	C	C	6.805290e-01	9.790634e-01	5.723118e-01	9.610389e-01	3.180805e-02

	DNANCBContrast	DNAShufflContrast	DNAShufflNCBContrast	tRNA	rRNA	ncDNA
1	3.214075e-03	5.938989e-02	1.236036e-03	2.225567e-03	1.462637e-05	5.302653e-01
3	5.557688e-05	2.436041e-06	3.340970e-04	3.127925e-06	9.334995e-13	8.635661e-01
5	1.931562e-03	1.107030e-01	7.716097e-03	9.007908e-08	1.766293e-04	7.360319e-01
7	4.558475e-09	8.542999e-03	3.908562e-11	3.980484e-04	3.576055e-13	2.794794e-01
9	8.139079e-06	3.858728e-05	8.650850e-06	5.103282e-09	2.827619e-17	1.028559e-01
11	4.244390e-01	4.661724e-01	6.537325e-01	2.103232e-14	7.379248e-10	6.028416e-01
13	3.851448e-03	6.176145e-01	6.616718e-04	2.541081e-07	1.117713e-08	5.365893e-01
15	3.745785e-05	3.251837e-05	2.503934e-05	2.526286e-14	2.076206e-11	1.237966e-05
17	1.691788e-05	1.888432e-10	4.807844e-04	1.255028e-05	1.037018e-02	4.945179e-06
19	3.411232e-07	5.572190e-05	3.656181e-07	2.400798e-09	7.563835e-11	3.637666e-01
21	5.910374e-01	8.953674e-01	9.489587e-01	0.000000e+00	0.000000e+00	3.571576e-01
23	1.790529e-01	4.877980e-02	6.420683e-01	3.971170e-09	3.898482e-11	5.690241e-01
25	1.727557e-03	2.776355e-01	1.773748e-03	7.066907e-04	5.165532e-09	6.128928e-01
27	8.633744e-01	1.839442e-02	8.088337e-01	2.742259e-19	5.766187e-14	8.444337e-01
29	2.669572e-01	8.882607e-01	2.481888e-01	5.502487e-12	1.998401e-14	7.989705e-01
31	1.146847e-05	1.046177e-02	8.721221e-07	0.000000e+00	0.000000e+00	3.611098e-01

	tRNAContrast	rRNAContrast	ncDNAContrast
1	3.115286e-12	0.000000e+00	3.754489e-01
3	1.478836e-08	4.440892e-16	1.396026e-02
5	1.732016e-07	3.045972e-10	3.146471e-01
7	2.071265e-04	2.256028e-17	2.304914e-04
9	8.026640e-02	4.203047e-01	7.373518e-04
11	3.431078e-11	4.850813e-02	2.797421e-01
13	2.029342e-04	7.850981e-01	4.504017e-01
15	8.307883e-12	8.240780e-04	2.257787e-06
17	1.985257e-03	2.900141e-06	1.678292e-06
19	4.499909e-03	1.665569e-08	1.559645e-04
21	2.505547e-03	2.087908e-08	9.849944e-01
23	7.094266e-01	8.599977e-02	3.403784e-02
25	1.079163e-04	3.108858e-14	4.594499e-01
27	7.106424e-12	6.816048e-05	2.549871e-01
29	2.935892e-01	5.376915e-08	9.762280e-01
31	2.873657e-11	1.382006e-12	9.642523e-01

Correlation between OGT and nucleic acid composition of DNA, RNA and ncDNA in Bacteria

	sk	base	DNANat	DNANCB	tRNA	rRNA	ncDNA
2	B	A	0.2389380	0.221110253	4.384306e-05	5.839117e-07	0.8086705
4	B	T	0.8044612	0.207176665	1.122328e-06	5.144195e-17	0.8893945
6	B	G	0.8631355	0.879971957	4.192170e-07	3.197442e-14	0.8391709
8	B	C	0.2799994	0.002196292	3.844750e-04	4.501429e-09	0.8589468

Correlation between OGT and dinucleotide composition of DNA, RNA and ncDNA in Bacteria

	sk	base1	base2	DNANat	DNANCB	DNAShuffledCodons	DNAShuffledCodonsNCB	DNANatContrast
2	B	A	A	1.288774e-01	9.098009e-02	2.088845e-01	1.068251e-01	6.309651e-01
4	B	T	A	9.258460e-01	1.236997e-01	5.986421e-01	8.148167e-02	9.047734e-01
6	B	G	A	6.929348e-09	9.804471e-09	6.507039e-11	3.244154e-08	1.311695e-02
8	B	C	A	5.973944e-05	1.839312e-12	3.372642e-05	2.947969e-12	3.024141e-05
10	B	A	T	5.828502e-01	8.388291e-01	8.189045e-01	7.861537e-01	1.764149e-03
12	B	T	T	7.070029e-01	3.800722e-02	9.254197e-01	4.281989e-02	1.053985e-02
14	B	G	T	1.145087e-01	3.229258e-02	1.680596e-01	8.987065e-03	1.039960e-01
16	B	C	T	6.212355e-01	3.883475e-03	4.912522e-01	1.406572e-03	1.972994e-03
18	B	A	G	7.972932e-04	1.145264e-10	8.443731e-05	6.442114e-11	3.159727e-02
20	B	T	G	7.255524e-03	1.228312e-01	1.517063e-02	2.244161e-01	7.242761e-04
22	B	G	G	1.087723e-01	6.963199e-01	3.416003e-01	8.540609e-01	2.062467e-04
24	B	C	G	3.014042e-01	3.190664e-01	3.795844e-01	4.075656e-01	7.291748e-02
26	B	A	C	9.839235e-01	4.700975e-07	5.979826e-01	1.729999e-06	3.933598e-01
28	B	T	C	5.929617e-01	1.656711e-01	1.165062e-01	2.186517e-02	1.057273e-01
30	B	G	C	4.347739e-02	2.187252e-02	1.213831e-01	3.911473e-02	1.108723e-05
32	B	C	C	8.949194e-01	1.911637e-01	5.963681e-01	9.591056e-02	9.044732e-04

	DNANCBContrast	DNAShufflContrast	DNAShufflNCBContrast	tRNA	rRNA	ncDNA
2	1.082807e-02	2.510516e-01	2.792562e-02	3.969641e-01	1.021652e-03	0.45704385
4	6.311172e-01	4.844554e-01	2.594739e-01	4.873019e-08	2.717309e-08	0.76870123
6	4.994314e-06	1.640118e-02	1.933257e-05	5.046506e-02	5.492471e-01	0.19026927
8	1.554166e-15	2.950904e-05	2.418211e-15	4.326951e-01	1.720797e-03	0.04349543
10	5.840913e-06	3.323749e-03	7.440650e-06	2.567896e-05	3.457074e-15	0.69702646
12	3.408125e-04	6.434111e-02	6.847059e-04	2.857442e-05	2.875131e-13	0.54340271
14	2.265124e-02	1.569071e-01	4.961455e-04	1.013490e-01	1.784689e-06	0.12513703
16	2.689747e-02	2.916420e-04	1.115649e-01	7.374928e-04	1.371177e-08	0.31396245
18	1.963424e-02	3.425275e-03	3.529447e-02	3.266237e-01	1.355153e-01	0.25533926
20	1.415716e-07	2.121502e-03	1.241424e-07	8.663553e-02	3.630880e-08	0.01297856
22	4.310957e-01	3.481160e-03	9.757070e-01	2.573415e-08	0.000000e+00	0.29794155
24	6.300758e-03	4.628607e-02	2.600917e-04	1.158657e-04	1.378138e-08	0.38378331
26	7.189454e-03	7.671928e-02	3.415938e-02	4.789952e-03	1.220633e-06	0.23603065
28	3.751332e-02	4.315045e-01	1.263913e-01	1.268653e-01	1.371700e-05	0.17429672
30	1.079053e-08	3.108755e-05	4.531783e-07	1.164747e-05	1.884226e-11	0.14627947
32	0.000000e+00	1.060502e-03	0.000000e+00	1.887347e-04	2.220446e-15	0.30876415

	tRNAContrast	rRNAContrast	ncDNAContrast
2	2.243247e-05	1.161737e-11	1.657111e-01
4	2.929890e-04	1.525686e-02	7.962262e-01
6	1.353880e-01	7.061758e-03	1.175852e-01
8	8.255289e-01	1.551412e-07	9.950798e-04
10	4.869668e-02	1.759083e-07	2.504995e-02
12	6.494346e-01	5.941685e-01	1.509447e-01
14	3.075272e-01	5.034010e-02	1.287298e-01
16	6.290221e-01	4.930305e-01	2.050469e-01
18	6.782729e-01	1.278875e-01	2.441247e-01
20	8.741252e-01	2.468562e-01	4.799879e-04
22	7.001866e-01	6.276559e-03	2.715224e-06
24	9.245854e-01	6.673869e-03	9.693277e-03
26	1.948665e-03	2.283439e-10	1.354998e-01
28	1.250145e-01	1.703297e-01	6.343713e-02
30	7.085675e-01	9.763395e-02	3.039667e-06
32	2.803002e-01	5.680553e-07	9.677785e-06
