# Supplementary material for: Molecular mechanisms of adaptation emerging from the physics and evolution of nucleic acids and proteins
Source: Nucleic Acids Res. 2013 Dec 25;42(5):2879–92. doi: 10.1093/nar/gkt1336 (PMC3950714; doi:10.1093/nar/gkt1336)
Supplement: Supplementary Data [file supp_gkt1336_nar-02158-n-2013-File011.docx]

**Supplementary File 9**

**Position-independent nucleic acid composition in ncDNA and RNA, t-tests**

**Nucleic composition comparison of DNA, RNA and ncDNA in Archaea**

sk base DNANatFreq DNANCBFreq tRNAFreq rRNAFreq ncDNAFreq

1 A A 0.0003646696 5.927512e-10 0 7.747389e-04 3.078713e-09

3 A T 0.0946029887 8.662644e-04 0 2.331468e-13 2.255309e-09

5 A G 0.1495192534 1.025506e-03 0 0.000000e+00 2.547662e-09

7 A C 0.0004101233 0.000000e+00 0 9.853684e-01 2.624919e-09

**Dinucleotide composition comparison of DNA, RNA and ncDNA in Archaea**

sk base1 base2 DNANatContrast DNANCBContrast DNA31ShuffledContrast DNA31ShuffledNCBContrast

1 A A A 3.296417e-01 6.304431e-04 2.224894e-01 1.467400e-03

3 A T A 9.620250e-09 0.000000e+00 6.156314e-08 0.000000e+00

5 A G A 1.664465e-09 0.000000e+00 7.672529e-12 0.000000e+00

7 A C A 2.020666e-01 0.000000e+00 4.044179e-01 0.000000e+00

9 A A T 2.793122e-01 5.422081e-07 9.671220e-02 3.506032e-08

11 A T T 8.314678e-04 0.000000e+00 4.972778e-05 0.000000e+00

13 A G T 4.458656e-13 0.000000e+00 0.000000e+00 0.000000e+00

15 A C T 5.951266e-06 9.603293e-07 8.643374e-06 6.854541e-06

17 A A G 5.290955e-01 2.573497e-13 4.942846e-01 1.758593e-13

19 A T G 7.321504e-02 1.216122e-03 1.644121e-02 2.663018e-06

21 A G G 1.267562e-03 4.091380e-05 2.766678e-06 3.730419e-06

23 A C G 1.938876e-04 0.000000e+00 1.637772e-04 0.000000e+00

25 A A C 1.344634e-03 1.378377e-05 5.526922e-03 2.943452e-05

27 A T C 4.933713e-04 1.649639e-08 1.030857e-04 1.611536e-08

29 A G C 9.211547e-01 2.595287e-04 2.019973e-01 7.554536e-04

31 A C C 3.034540e-03 3.296318e-04 1.176073e-02 1.545687e-03

tRNAContrast rRNAContrast ncDNAContrast

1 1.398881e-14 1.598721e-14 3.017706e-10

3 1.202804e-04 9.468667e-02 3.230820e-08

5 2.437295e-11 6.661513e-04 5.621343e-02

7 4.621844e-03 0.000000e+00 2.213176e-04

9 2.408013e-02 0.000000e+00 2.902780e-01

11 6.580107e-04 6.407789e-02 1.870524e-10

13 1.690184e-07 1.129844e-08 1.574652e-11

15 8.711302e-06 2.580891e-11 3.188069e-01

17 1.953993e-14 5.093077e-01 3.396140e-01

19 0.000000e+00 6.933327e-01 1.977734e-04

21 0.000000e+00 1.733263e-07 4.849025e-09

23 0.000000e+00 3.774758e-14 5.238973e-05

25 0.000000e+00 5.222045e-12 1.123723e-11

27 0.000000e+00 9.592091e-01 4.773235e-02

29 0.000000e+00 0.000000e+00 7.052208e-01

31 0.000000e+00 0.000000e+00 6.115614e-09

**Nucleic composition comparison of DNA, RNA and ncDNA in Bacteria**

sk base DNANatFreq DNANCBFreq tRNAFreq rRNAFreq ncDNAFreq

2 B A 5.578136e-02 8.440804e-11 0 0 0.0005845965

4 B T 6.823775e-09 0.000000e+00 0 0 0.0004368072

6 B G 1.495684e-10 0.000000e+00 0 0 0.0005270684

8 B C 1.266939e-01 0.000000e+00 0 0 0.0004879509

**Dinucleotide composition comparison of DNA, RNA and ncDNA in Bacteria**

sk base1 base2 DNANatContrast DNANCBContrast DNA31ShuffledContrast DNA31ShuffledNCBContrast

2 B A A 0.000000e+00 0.000000e+00 0.000000e+00 0.000000e+00

4 B T A 0.000000e+00 0.000000e+00 0.000000e+00 0.000000e+00

6 B G A 9.325873e-15 0.000000e+00 0.000000e+00 0.000000e+00

8 B C A 8.377743e-12 1.559626e-03 9.462090e-01 5.326231e-01

10 B A T 0.000000e+00 0.000000e+00 0.000000e+00 0.000000e+00

12 B T T 0.000000e+00 0.000000e+00 4.114888e-08 0.000000e+00

14 B G T 0.000000e+00 0.000000e+00 0.000000e+00 0.000000e+00

16 B C T 4.542502e-02 0.000000e+00 5.801567e-06 0.000000e+00

18 B A G 0.000000e+00 0.000000e+00 0.000000e+00 0.000000e+00

20 B T G 0.000000e+00 3.313323e-01 0.000000e+00 2.226449e-02

22 B G G 1.065814e-14 0.000000e+00 8.320278e-11 0.000000e+00

24 B C G 2.902233e-03 3.551826e-12 4.081849e-05 3.235193e-05

26 B A C 0.000000e+00 0.000000e+00 0.000000e+00 0.000000e+00

28 B T C 2.295099e-01 7.650881e-02 3.937523e-02 1.836180e-02

30 B G C 0.000000e+00 0.000000e+00 0.000000e+00 0.000000e+00

32 B C C 5.484502e-13 0.000000e+00 2.498335e-05 0.000000e+00

tRNAContrast rRNAContrast ncDNAContrast

2 0.02562858 0.000000e+00 0.000000e+00

4 0.00000000 6.733725e-12 0.000000e+00

6 0.00000000 1.090354e-07 7.335116e-03

8 0.00000000 0.000000e+00 1.442489e-01

10 0.00000000 0.000000e+00 5.245333e-02

12 0.00000000 0.000000e+00 0.000000e+00

14 0.00000000 0.000000e+00 0.000000e+00

16 0.00000000 0.000000e+00 0.000000e+00

18 0.00000000 8.287125e-03 0.000000e+00

20 0.00000000 0.000000e+00 1.954418e-01

22 0.00000000 8.881784e-15 4.695410e-06

24 0.00000000 0.000000e+00 8.926321e-01

26 0.00000000 8.926193e-14 0.000000e+00

28 0.00000000 0.000000e+00 7.637044e-03

30 0.00000000 0.000000e+00 0.000000e+00

32 0.00000000 0.000000e+00 5.790471e-06
